# Universal mass spectrometric analysis of poly(ionic liquid)s†
†Electronic supplementary information (ESI) available: All synthetic procedures, additional characterization (SEC, NMR), and all additional mass spectrometric material. See DOI: 10.1039/c6sc01347c


**DOI:** 10.1039/c6sc01347c

**Published:** 2016-04-21

**Authors:** Martina M. Cecchini, Jan Steinkoenig, Samantha Reale, Leonie Barner, Jiayin Yuan, Anja S. Goldmann, Francesco De Angelis, Christopher Barner-Kowollik

**Affiliations:** a Dipartimento di Scienze Fisiche e Chimiche , Università degli Studi dell'Aquila , Via Vetoio , Coppito , 67100 , L'Aquila , Italy . Email: francesco.deangelis@univaq.it; b Preparative Macromolecular Chemistry , Institut für Technische Chemie und Polymerchemie , Karlsruhe Institute of Technology (KIT) , Engesserstr. 18 , 76128 Karlsruhe , Germany . Email: christopher.barner-kowollik@kit.edu; c Institut für Biologische Grenzflächen , Karlsruhe Institute of Technology (KIT) , Hermann-von-Helmholtz-Platz 1 , 76344 Eggenstein-Leopoldshafen , Germany; d Soft Matter Synthesis Laboratory , Institut für Biologische Grenzflächen , Karlsruhe Institute of Technology (KIT) , Hermann-von-Helmholtz-Platz 1 , 76344 Eggenstein-Leopoldshafen , Germany; e Max-Planck-Institute of Colloids and Interfaces , Research Campus Golm , 14424 Potsdam , Germany

## Abstract

We introduce a universal high resolution mass spectrometric method for the analysis of poly(ionic liquid)s (PILs), which belong to the most challenging polyelectrolytes from an analytical perspective, by fusing high resolution collision-induced dissociation (CID)-Orbitrap mass spectrometry (MS) with supercharging agents as well as quadrupole time-of-flight (QToF) MS. The study includes a wide array of hydrophilic halide-containing PILs, which were analyzed in negative mode. The influence of the core structures (based on imidazolium, triazolium, ammonium, phosphonium and pyridinium moieties), and variable styrene-, acrylate- and vinyl-type IL polymers on the ionization behavior is mapped in detail. Variable end group functionalities were introduced *via* functional chain transfer agents (CTA) in reversible addition-fragmentation chain transfer (RAFT) polymerization to study their behavior during the MS analysis. Furthermore, the demanding class of vinylimidazolium halide IL polymers was investigated. The current contribution thus introduces a new analytical technology platform for an entire polymer class.

## Abstract 2

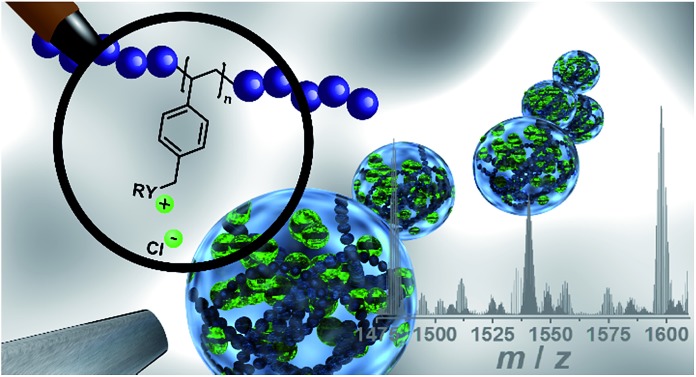
We introduce a universal tool for the mass spectrometric analysis of a wide range of various types of poly(ionic liquids).

## Introduction

Ionic liquids (ILs) are organic salts characterized by a melting point below 100 °C, which find significant applications in chemistry, physics and materials science.^1^ The polymerization of monomeric ILs results in poly(ionic liquid)s (PILs)^2^–^4^ either broadly dispersed *via* free radical polymerization (FRP)^5^ or narrowly dispersed *via* reversible deactivation radical polymerization (RDRP), such as reversible fragmentation-addition chain transfer polymerization (RAFT),^6^–^8^ atom transfer polymerization (ATRP)^9^,^10^ or organometallic-mediated radical polymerization (OMRP) for the polymerization of the demanding vinylimidazolium ILs.^11^,^12^ PILs combine the scope of ionic polymers by fusing the characteristics of the polymer with those of the IL. For instance, PILs display solubility in a wide range of polar and non-polar solvents,^13^,^14^ show structural diversity arising from a combination of core structures and counter ions,^8^,^15^ and possess widely tunable material characteristics.

The vast number of monomeric ILs is best classified according to their core, since the IL properties are altered by adjusting the core structure along with their attached moieties. The most prominent candidates are imidazolium, pyridinium, ammonium, phosphonium and sulfonium units. For instance, imidazolium and ammonium PILs find application as CO_2_ storage materials due to their specific interaction with CO_2_.^16^ Ammonium and phosphonium PILs act as (biodegradable) DNA delivery systems with lower critical solution temperature (LCST) characteristics.^17^,^18^ A further classification by the type of monomer attached to the core structure is recommended. Currently, the polymerization of monomeric ILs is dominated by either styrenic,^10^,^19^ (meth)acrylic^20^ or vinylic moieties.^11^,^12^,^21^ Independent of their classification, PILs are characterized by a wide structure diversity that addresses an unusually broad spectrum of properties and functions^6^,^22^ for material design.

Currently, the characterization of PILs is achieved by nuclear magnetic resonance (NMR) spectroscopy and size exclusion chromatography (SEC), which is subdivided into aqueous SEC – suitable for halogen-based PILs^6^,^22^ – and THF SEC using LiTf_2_N as additive – suitable for hydrophobic PILs.^9^ Recently, we reported the mass spectrometric (MS) investigation of PILs *via* electrospray ionization quadrupole (ESI-Q) MS, matrix assisted laser desorption ionization-time-of-flight (MALDI-ToF) MS, and surface-attached PILs *via* ToF-secondary ion mass spectrometry (SIMS).^6^ However, neither of these two approaches allows for an accurate mapping of chain structures, due to the low resolution of the ESI-Q (∼5000). Furthermore, ESI-Q MS is restricted to low masses (*i.e.* 4000 Th for single charged species).

As a powerful analytical tool, mass spectrometry gained increasing attention within the field of macromolecular chemistry, biochemistry and supramolecular chemistry. Especially the structural information afforded by tandem mass spectrometry *via* collision-induced dissociation (CID) or higher-energy collision dissociation (HCD) find significant application in polymeromics,^23^ and mechanistic (polymerization) studies.^24^ To obtain reliable mass spectra from polymer samples *via* ESI MS, the polymeric material needs to meet key criteria such as possessing Lewis acceptor or donor binding sites (*e.g.* for deprotonation in the negative mode or proton attachment in the positive mode), solubility in solvents commonly used for ESI MS measurements, and to obey the mass limitation, which is dictated by the employed mass analyzer.^25^

Nowadays, the development of methods to analyze biomacromolecules – especially proteins – has led to a wealth of ionization protocols. In particular, the well-studied, however yet not fully understood, supercharging of proteins beyond 20 kDa heralded the decade of proteomics, which has been recently hailed as the most significant innovation in mass spectrometry.^26^ In addition to supercharging, different auxiliary additives to the ESI solvents enabled the determination of poorly ionizable polymers.^27^

**Fig. 1:**
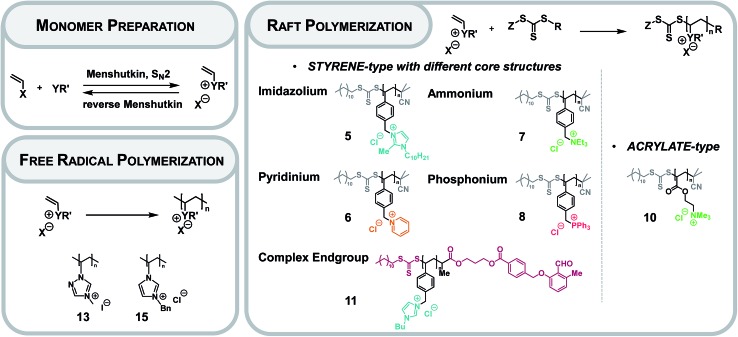
Overview over the IL monomer preparation (X: halide; Y: N or P or S; R′: alkyl or aryl) as well as the free radical and RAFT polymerization collating all investigated PILs in the present study (Z: stabilizing group, R: leaving group).

We herein introduce a technology platform to characterize PIL chain structures by fusing proteomics based supercharging technology with high-resolution ESI-Orbitrap MS and ESI-QToF MS. RAFT-prepared PILs bearing different types of core structures (pyridinium, ammonium, phosphonium, imidazolium) and non-controlled FRP-prepared PILs (based on imidazolium and triazolium core structures) form the analytical base library (Fig. 1). Furthermore, the technology was applied to acrylate-type PILs with variable side groups.

## Results and discussion

### Preparation of PILs

The preparation of monomeric ILs is achieved *via* a quaternization of the core with the desired (meth)acrylate- or styrene-type functionality (Fig. 1).^2^ A set of monomeric ILs is prepared ranging from imidazolium as core structure to ammonium, phosphonium and pyridinium compounds. As the RAFT process features a high tolerance towards functional monomers, excellent end group fidelity and control over the molecular weight, the RAFT polymerization of monomeric ILs was performed with 2-cyano-2-propyl dodecyl trithiocarbonate (CPDT) as chain transfer agent (CTA) or photo reactive functionalized (dodecylthiocarbonothioylthio)propionic acid (DoPAT).

### ESI MS characterization of styrenic PILs bearing variable core structures

We initially focus on an in-depth ESI MS investigation of four PILs with variable core structures: imidazolium, pyridinium, ammonium and phosphonium. The ESI MS spectra have been recorded in negative ion mode, employing the in-source Collision Induced Dissociation (CID) fragmentation technique. CID fragmentation is a technique of tandem mass spectrometry to induce collisions between ions and neutral gas molecules. The collision energy employed for tandem MS in the Q-Exactive Orbitrap MS ranges from 5 up to 100 eV. The in-source CID fragmentation technique allows to induce collisions without the selection of the precursor ions, causing the collisional activation of all ions emitted by the electrospray source. The resulting spectrum is thus a collection of the precursor ions and the product ions.^28^ Applying this strategy to PILs resulted in a sensitive polyelectrolyte detection, without significant dissociative events (refer to Fig. S28†).^29^ Since PILs feature strong intra- and intermolecular ionic interactions,^30^ the in-source CID fragmentation might induce a declustering of polymeric chains. As a result, single PIL chains ionized by the halide in the negative mode can be detected rather than polymer clusters that are nearly undetectable. Importantly and in addition to CID, we took advantage of the supercharging effect. Since the repeating unit of PILs can separate corresponding peaks by several hundred Da, a division by two (for double charged) or three (for triple charged) has a significant impact on MS spectra of PILs.

Donald and colleagues^31^ have reported the use of propylene carbonate (PC) and ethylene carbonate (EC) as supercharging additives to reach higher protonation states of proteins such as ubiquitin, cytochrome c, and carbonic anhydrase II. Currently, the mechanism of supercharging is debated.^32^ The Berkeley mechanism^33^ proposes that the high surface tension of a non-volatile supercharging reagent causes a late formation of the droplets in the electrospray source and a higher charge density in the ionic droplets. As a result, the Coulomb fission is delayed and a high number of charges are transferred to analytes released from these droplets. Douglass and Venter^32^ have refuted the Berkeley mechanism stating that supercharging additives with a high dipole moment can interact with proteins by specific ion–dipole interactions, protecting the ionic sites and reducing the repulsive forces between the ion charge states. Indeed, in our investigations, propylene carbonate is shown to play a key-role in the extent of charging PILs in the negative electrospray ionization mode, allowing for the detection of double and triple charged species (refer to Fig. S29†). The proposed PIL supercharging may be attributed to a high halide concentration at the moment of ion formation. In addition, the ESI droplet disintegration may occur at a later stage allowing the small, highly charged droplets to penetrate deeper into the spectrometer before releasing gas phase ions. The combination of the CID fragmentation technique with a supercharging additive led us to the development of a novel mass spectrometric method for the in-depth structural elucidation of PILs.

### Imidazolium as core structure

**Fig. 2:**
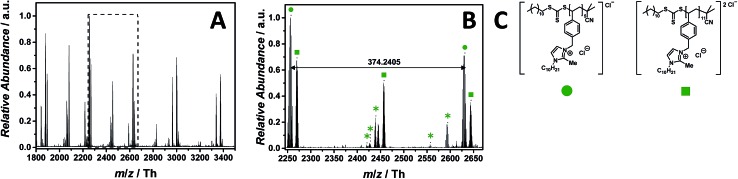
ESI-CID-Orbitrap MS spectra of p([DeMVBIM]Cl) (**5**) (*M*_n_ = 4900 g mol^–1^, *Ð* = 1.49) depicting (A) the overview spectrum from 1800 Th to 3500 Th; (B) zoom into one repeating unit (*m*/*z*(exp) = 374.2405 Th, *m*/*z*(theo) = 347.2489 Th); (C) proposed structures of the most abundant species labelled with 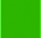 and 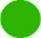. For all peak assignments refer to Table S1.† Species labelled with 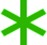 derive from (multiple) loss(es) of gaseous HCl.

PILs consisting of an imidazolium core belong to the most widely employed systems. The ESI-CID-Orbitrap measurement of poly(1-decyl-2-methyl-3-(4-vinylbenzyl)-1*H*-imidazol-3-ium chloride) (p([DMVBIM]Cl), **5**) was performed in the negative mode, doping the solvent (water/acetonitrile (1 : 1, v/v)) with 2.0% (v/v) propylene carbonate. The ESI MS profile (ranging from *m*/*z* = 1800 Th up to *m*/*z* = 3500 Th) (Fig. 2A) clearly depicts a main distribution of single charged ions, whose proposed structure is reported in Fig. 2C. A second single charged distribution associated with the neutral loss of HCl (labelled with 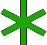) and a less abundant distribution of double charged species (labelled with 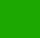) are also observed (Fig. 2B). The propylene carbonate has a critical effect in the supercharging of **5**, enabling the detection of the double charged ions. The zoom (Fig. 2B) depicts the repeating unit *m*/*z* = 374.2405 Th (*m*/*z*(theo) = 347.2489 Th) of the single charged species (labelled with 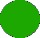). Furthermore, the double charged species (labelled with 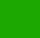) is separated by *m*/*z* = 187.1175 Th (*m*/*z*(theo) = 187.1203 Th). The ions arising from several neutral losses of HCl (labelled with 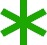) are the only observed product ions. Schubert^34^ and Stevens^35^ showed that *N*-benzyl substituted ionic liquids with chloride as counter ion have an increased thermal stability in comparison to their alkyl analogues, supporting the finding that no ionic species derived from imidazolium degradation are observed. Ohtani *et al.* performed a pyrolysis-gas chromatography (Py-GC) study regarding the thermal degradation of imidazolium-based ILs, stating that the main pathway of thermal decomposition occurs *via* a reverse Menshutkin reaction.^36^ During the tandem MS experiment performed on the ion at *m*/*z* = 2270 ± 10 Th (refer to Fig. S35†), a high stability of the imidazolium moiety is evident since no fragments of a reverse Menshutkin reaction were detected.

### Pyridinium as core structure

**Fig. 3:**
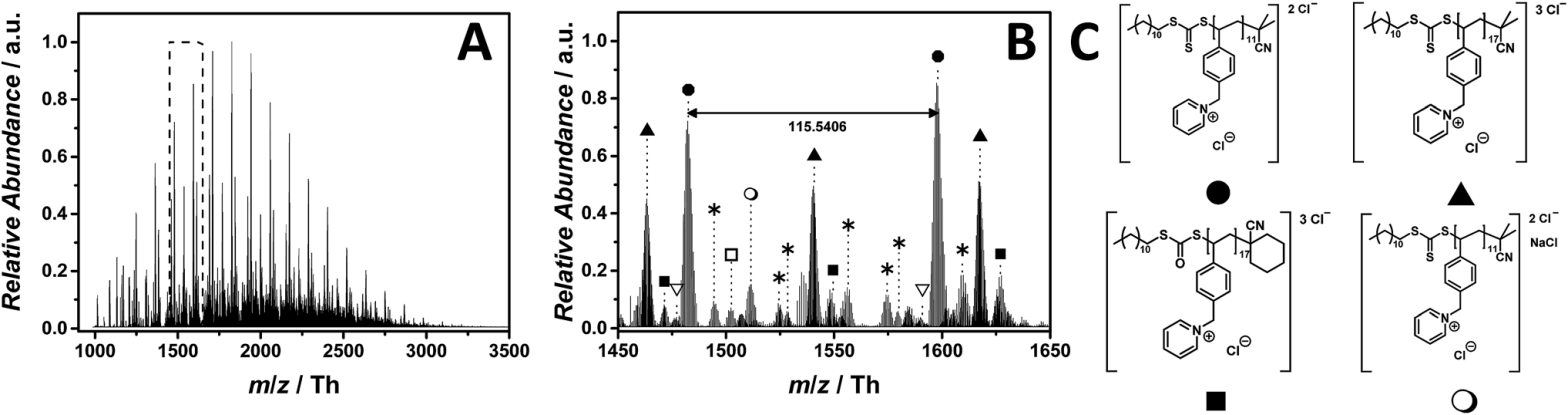
ESI-CID-Orbitrap MS spectra of p([VBPy]Cl) (**6**) (*M*_n_ = 1800 g mol^–1^, *Ð* = 1.8) depicting (A) the overview spectrum from 1000 Th to 3500 Th; (B) zoom into one repeating unit (*m*/*z*(exp) = 115.5406 Th, *m*/*z*(theo) = 115.5412 Th); (C) proposed structure of the most abundant species. For all peak assignments refer to Table S2.† Species labelled with 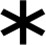 derive from (multiple) loss(es) of gaseous HCl.

Pyridinium-based PILs have not attracted much attention in the literature. The chemical difference of pyridinium in comparison with other well-known core structures are significant, making poly(1-(4-vinylbenzyl)pyridin-1-ium chloride) (p([VBPy]Cl), **6**) an interesting candidate for a mass spectrometric investigation. As observed for the imidazolium core structure **5**, the ESI-CID-Orbitrap measurements of p([VBPy]Cl) (**6**) were performed in negative mode in water/acetonitrile (1 : 1, v/v). During the analysis of p([VBPy]Cl) (**6**), a CID fragmentation of 10 eV is critical for the detection of the polymer (Fig. S28†). Without the addition of a supercharging agent, the ionization in the electrospray source provides double and triple negatively charged species (Fig. 3C). The ESI MS profile has the typical Gaussian shape of a polymer (Fig. 3A) in a mass range from *m*/*z* = 1000 Th to *m*/*z* = 3500 Th. The zoom (Fig. 3B) highlights the repeating peaks of the double (labelled with 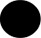) and triple (labelled with 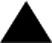) charged species. Interestingly, without the addition of a supercharging agent, p([VBPy]Cl) (**6**) was multiple charged. The repeating unit of the double charged species is *m*/*z* = 115.5406 Th (*m*/*z*(theo) = 115.5412 Th), while the triple charged species are separated by *m*/*z* = 77.0283 Th (*m*/*z*(theo) = 77.0277 Th). The zoom (Fig. 3B) indicates a cyclohexanecarbonitrile moiety stemming from the initiator 1,1′-azobis-(cyclohexanecarbonitrile) rather than the expected 2-cyano-2-propyl moiety. The corresponding species at *m*/*z* = 1476.8310 Th (labelled with 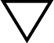 for the triple charged ion) and *m*/*z* = 1502.5176 Th (labelled with 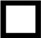 for the double charged ion) clearly identify these end groups. Furthermore, the trithiocarbonate group undergoes a partial oxidation, producing the triple charged ions at *m*/*z* = 1471.4959 Th (labelled with 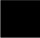). Further species of minor abundance can be assigned to multiple neutral losses of HCl (labelled with 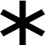) (refer to Table S2† for the complete list of assignments). A significant contribution to the detected end group variety of p([VBPy]Cl) (**6**) can be attributed to the employed CID fragmentation during the acquisition of the mass spectrum. Crosthwaite *et al.* investigated the thermal stability of pyridinium and showed a significant difference between a pyridinium and an imidazolium halide,^37^ supporting the findings that – during tandem MS analysis of p([VBPy]Cl) (**6**) performed on the ion at *m*/*z* = 1482 ± 10 Th – a fragmentation according to the reverse Menshutkin mechanism (Fig. S41 and Scheme S1†) is operational.

### Ammonium as core structure

**Fig. 4:**
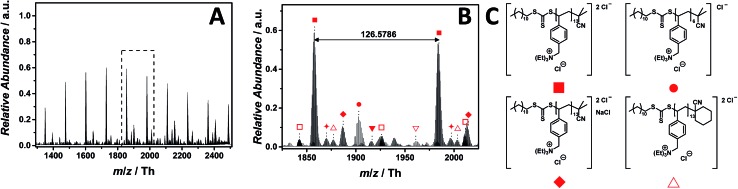
ESI-CID-Orbitrap MS spectra of p([TEVBA]Cl) (**7**) (*M*_n_ = 2100 g mol^–1^, *Ð* = 1.8) depicting (A) overview spectrum from 1300 Th to 2500 Th; (B) zoom into one repeating unit (*m*/*z*(exp) = 126.5786 Th, *m*/*z*(theo) = 126.5799 Th); (C) proposed structure of the most abundant species. For all peak assignments refer to Table S4.†

Ammonium-based PILs are frequently applied for DNA delivery and as temperature-responsive polymers.^17^,^38^ The ESI-CID-Orbitrap measurements of poly(*N*,*N*,*N*-triethyl-*N*-(4-vinylbenzyl)ammonium chloride) (p([TEVBA]Cl), **7**) were performed similar to those of p([VBPy]Cl) (**6**). A CID fragmentation energy of 14 eV has been used as well as 0.5% (v/v) of propylene carbonate as supercharging additive to achieve a Gaussian distribution of double charged species ranging from *m*/*z* = 1300 Th to *m*/*z* = 2500 Th (Fig. 4A), whose proposed structure is depicted in Fig. 4C. Again, propylene carbonate played a key role in detecting the double charged species rather than the low abundant single charged species (Fig. S29†). The zoom (Fig. 4B) illustrates the double charged species with the repeating unit of *m*/*z* = 126.5786 Th (*m*/*z*(theo) = 126.5799 Th) labelled with 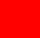. Furthermore, a less abundant distribution of single charged ions (labelled with 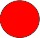) is observed. Additional species were observed during the MS experiment having the cyclohexanecarbonitrile moiety (labelled with 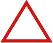) of the initiator as end group. With a rather dominant abundance, NaCl adducts were detected labelled with 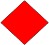, 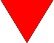, and 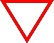 (Table S4† for the complete list of assignment). Interestingly, no species stemming from a loss of HCl were observed. In comparison to the imidazolium candidates, ammonium-based ILs are less used due to their reduced thermal stability. Since the physical properties of PILs significantly arise from that of ILs, ammonium-based PILs show a similar trend as observed for the ILs.^39^ As evidenced by Long and colleagues,^39^ they decompose *via* two pathways: (1) Hofmann elimination, in which the halide counter ion provokes the abstraction of a β-hydrogen producing a neutral tertiary amine with an alkene and hydrogen halide as by-products, and (2) the most common pathway, the Menshutkin reaction. Nevertheless, no species related to the nucleophilic attack of the chloride at the electrophilic benzyl moiety were observed during the full MS experiment employing 14 eV CID energy. During tandem MS experiments performed on the ion at *m*/*z* = 1731 ± 10 Th, the reverse Menshutkin reaction is the main fragmentation pathway, confirming the structure of the PIL proposed in Fig. 4C (Fig. S47 and Scheme S2†).

### Phosphonium as core structure

In phosphonium-based PILs, the electrophilic phosphonium group can function as homogenous polymer-supported reagent in a Wittig reaction,^40^ have significant gene delivery characteristics and phosphonium-decorated surfaces are potentially antimicrobial.^41^

**Fig. 5:**
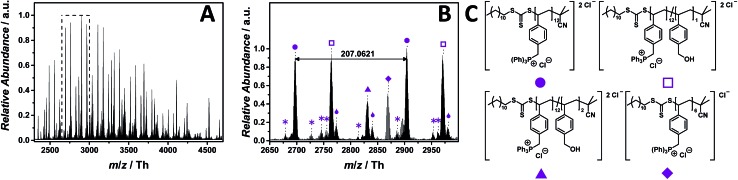
ESI-CID-Orbitrap MS spectra of p([TPVBP]Cl) (**8**) (*M*_n_ = 4200 g mol^–1^, *Ð* = 1.6) depicting (A) the overview spectrum from 2250 Th to 4750 Th; (B) zoom into one repeating unit (*m*/*z*(exp) = 207.0621 Th, *m*/*z*(theo) = 207.0658 Th); (C) proposed structure of the most abundant species. For all peak assignments refer to Table S5.† Species labelled with 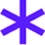 derive from (multiple) loss(es) of gaseous HCl.

ESI-CID-Orbitrap mass spectra of poly(triphenyl(4-vinylbenzyl)phosphonium chloride) (p([TPVBP]Cl), **8**) were obtained in negative mode utilizing water/acetonitrile (1 : 1, v/v) and 1.0% (v/v) propylene carbonate. Employing 25 eV as CID fragmentation energy facilitated the ionization of the phosphonium-based PIL. The ESI MS profile ranges from *m*/*z* = 2250 Th to *m*/*z* = 4750 Th (Fig. 5A). The zoom (Fig. 5B) depicts the repeating unit of the p([TPVBP]Cl) (**8**) with *m*/*z* = 207.0621 Th (*m*/*z*(theo) = 207.0658 Th) (labelled with 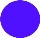) and, further, a fragmented species with similar abundance labelled with 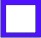 (Table S5†). Since the phosphonium group is highly electrophilic and oxygen attracting, the species (labelled with 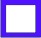) were assigned to a product where one triphenylphosphonium of the repeating unit is replaced with a hydroxyl group. The species at *m*/*z* = 2831.8759 Th (*m*/*z*(theo) = 2831.9073 Th) (labelled with 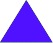) corresponds to a polymer chain, in which two repeating units fragment *via* the same nucleophilic attack. Interestingly, these species (labelled with 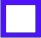 and 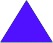) apparently bind water *via* strong hydrogen bonds (labelled with 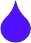).

During tandem MS experiments (performed on double charged species at *m*/*z* = 2282 ± 10 Th), a high stability of the bulky triphenylphosphine group is evident (Fig. S54†). Tandem MS performed on a single charged species at *m*/*z* = 1758 ± 5 Th indicates a multiple loss of HCl (Fig. S53†). Species deriving from the reverse Menshutkin reaction are not observed. However, it is known in the literature that phosphonium-based PILs have a decomposition temperature approx. 200 °C higher than their ammonium analogues.^39^

### ESI MS characterization of an acrylic PIL

The material diversity of PILs is – besides the core structure/counter ion – based on various monomer types (*e.g.* (meth)acrylate, functionalized vinyl moieties, styrene). To demonstrate the efficiency of the described mass spectrometric method, a further investigation focused on an acrylate-type PIL is reported. The in-depth ESI MS characterization of poly(2-(acryloyloxy)-*N*,*N*,*N*-trimethylethan-1-ammonium chloride) (p([ATMEA]Cl), **10**) was performed *via* the combination of a CID fragmentation energy of 25 eV and 2.0% (v/v) propylene carbonate in water/acetonitrile (1 : 1, v/v). Since the ester group has a sodium coordination site, we investigated if the acrylate-type chain allows to detect the PIL in positive ion mode. However, no sodium adducts were observed neither in positive nor in negative polarity.

**Fig. 6:**
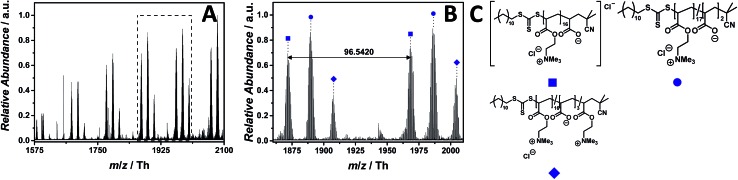
ESI-CID-Orbitrap MS spectra of p([ATMEA]Cl) (**10**) depicting (A) the overview spectrum from 1575 Th to 2100 Th; (B) zoom into one repeating unit (*m*/*z*(exp) = 96.5420 Th, *m*/*z*(theo) = 96.5440 Th); (C) proposed structure of the most abundant species. For all peak assignments refer to Table S7.†

The ESI MS profile (ranging from *m*/*z* = 1575 Th up to *m*/*z* = 2100 Th) (Fig. 6A) depicts different distributions of double charged ions. The proposed structure of one of these distributions is reported in Fig. 6C. The zoom (Fig. 6B) depicts the repeating unit of the species labelled with 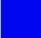 having a repeating unit of *m*/*z* = 96.5420 (*m*/*z*(theo) = 96.5440 Th). The most abundant species as part of a polymeric distribution (labelled with 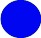) is composed of double charged ions containing two hydrolyzed repeating units (refer to Table S7† for a structural determination).

Thus, replacing the stable styrenic group against vulnerable acrylates results in charged PILs – for the first time observed – entirely *via* the main chain rather than *via* the counter ion. The work of Elabd and colleagues demonstrated for an imidazolium-based PIL that the hydrolysis of the carboxylate ester linkage takes place under alkaline conditions, normally after the preliminary ring-opening degradation mechanism occurring on the imidazolium moiety.^42^ An alternative hydrolysis pathway might convert the methacrylate-based homopolymer into a random copolymer, without any degradation of the imidazolium ring. The work of Elabd and colleagues underpins the copolymeric distributions (Fig. 6A). Furthermore, Baines and Bevington reported that poly(methyl acrylate) (p(MA)) hydrolyzes more rapidly than poly(methyl methacrylate) (p(MMA)) under the same alkaline conditions.^43^ However, we ascribe the hydrolysis of the acrylate backbone to both aqueous conditions used during the measurements and the employed CID fragmentation energy. A further, less abundant, double charged distribution was assigned to a different statistical copolymer (labelled with 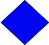). The proposed structure (Table S7†) consists of three cleaved ester bonds (with the negatively charged acid moiety) and one repeating unit deprived of the chloride counter ion.

The CID fragmentation energy of 25 eV was sufficient to afford the spectrum of p([ATMEA]Cl) (**10**) and a cleavage of the vulnerable ester bond. As an indirect confirmation of the proposed structure, the identified species produced directly in the full mass spectrum are considered as product ions of the p([ATMEA]Cl) (**10**).

### ESI MS characterization of a DoPAT-photo-enol end group functionalized imidazolium-based PIL

RAFT polymerization enables the advanced macromolecular design by block copolymer formation^44^ or modular ligation *via* functionalized CTAs. Especially, ‘click’ chemistry allows for a rapid preparation of sophisticated macromolecular architectures.^45^,^46^ Light-triggered modular ligations establish a spatial and temporal resolution under very mild and efficient conditions.^47^,^48^ Since functionalized CTAs for modular ligation need to induce excellent end group fidelity, mass spectrometric characterization plays a key role.

The following assesses if a cleavage of labile end group moieties such as aldehydes, ethers or esters – which were proven to be very labile for acrylate PILs – takes place. In addition, due to its diverse functionalities, the photo-enol end group could potentially coordinate to alkali metal cations (*e.g.* sodium) to enable the detection in positive mode. Considering the mass spectra of the p([DeMVBIM])Cl (**5**) (Fig. 2) we compare if the proposed mass spectrometric method can be applied for the structural elucidation of an imidazolium-based PIL bearing a more elaborate chain terminus.

**Fig. 7:**
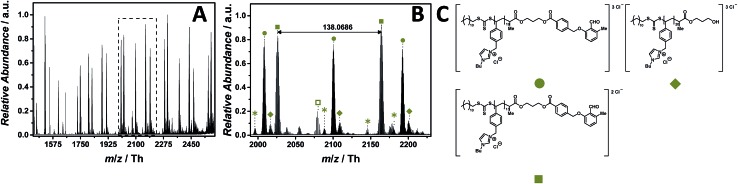
ESI-CID-Orbitrap MS spectra of p([BVBIM]Cl) (**11**) (*M*_n_ = 5900 g mol^–1^, *Ð* = 1.43) depicting (A) overview spectrum from 1450 Th to 2500 Th; (B) zoom into one repeating unit (*m*/*z*(exp) = 138.0686 Th, *m*/*z*(theo) = 138.0702 Th); (C) proposed structure of the most abundant species. For all peak assignments refer to Table S8.† Species labelled with 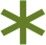 derive from (multiple) loss(es) of gaseous HCl.

The ESI-CID-Orbitrap measurements of poly(1-butyl-3-(4-vinylbenzyl)-1*H*-imidazol-3-ium chloride) (p([BVBIM]Cl), **11**) are performed in the negative mode, using a water/acetonitrile mixture (1 : 1, v/v) doped with 2.0% (v/v) propylene carbonate. 15 eV CID fragmentation energy was sufficient to provide a full mass spectrum (Fig. 7A) composed of single (labelled with 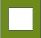), double (labelled with 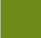) and triple (labelled with 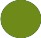) charged species ranging from *m*/*z* = 1450 Th to *m*/*z* = 2600 Th. The zoom (Fig. 7B) depicts the mainly double and triple charged species having a repeating unit of *m*/*z* = 138.0686 Th (*m*/*z*(theo) = 138.0702 Th) and *m*/*z* = 92.0444 Th, respectively. Based on the well-investigated thermal stability of the imidazolium-based PILs previously discussed, no by-products associated with the thermal decomposition of the 4-vinylbenzyl imidazolium scaffold were observed. The only by-product distribution observed in the mass spectrum results from the saponification of the ester linkage of the photo-enol moiety to the RAFT CTA (labelled with 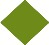). The hydrolysis can be explained by the water/acetonitrile solvent used to perform the ESI MS measurements in combination with the CID fragmentation energy that is sufficient to cleave ester bonds as observed for the acrylate-type PIL. In comparison with CPDT as CTA, neither does DoPAT-PE hamper the ionization of the entire polymer nor fragments rapidly due to the employed CID fragmentation energy. In addition, no sodium adducts were observed in both negative and positive mode.

### ESI MS characterization of PILs prepared *via* non-controlled free radical polymerization

RAFT polymerization is a comprehensive method for the preparation of well-defined polymers with a tunable molecular weight. During the polymerization of 1-vinylimidazolium ILs, the positive charge adjacent to the radical has a destabilizing effect and, hence, is difficult to polymerize in a controlled fashion. OMRP utilizing cobalt as mediating species affords the best control over these demanding monomers.^11^,^12^ However, poly(1-vinyl-3-alkyl-imidazolium) can be readily prepared *via* non-controlled FRP.^5^ The following section focuses on the mass spectrometric elucidation of the demanding 1-vinylimidazole-based PILs prepared *via* FRP. Herein, we report a successful approach for the ionization of poly(4-methyl-1-vinyl-1,2,4-triazolium iodide) (p([MVTr]I), **13**) and poly(1-benzyl-3-vinylimidazolium chloride) (p([BnVIM]Cl), **15**) in the negative mode without auxiliary supercharging agents.

Generally, triazolium-containing polyelectrolytes gained increasing attention due to their wide range of applications in the field of polymer chemistry.^49^ 1,2,3-Triazolium monomers obtained by the copper catalyzed azide alkyne cycloaddition (CuAAC) show a lower tendency to polymerize due to the proximity of the cationic ring to the vinyl backbone that confers rigidity to the polymeric chain, leading to an increase of the glass transition temperature (*T*_g_).

**Fig. 8:**
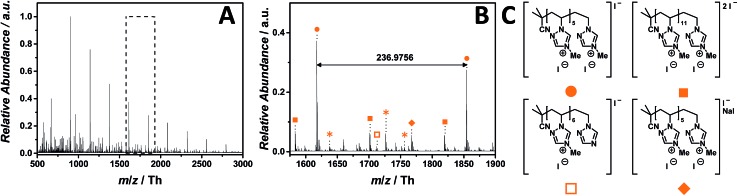
ESI-CID-Orbitrap MS spectra of p([MVTr]I) (**13**) (*M*_n_ = 47 000 g mol^–1^, *Ð* = 1.9) depicting (A) overview spectrum from 500 Th to 3000 Th; (B) zoom into one repeating unit (*m*/*z*(exp) = 236.9756 Th, *m*/*z*(theo) = 236.9763 Th); (C) proposed structure of the most abundant species. For all peak assignments refer to Table S9.† Species labelled with 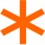 derive from (multiple) loss(es) of gaseous HI.

The ESI-CID-Orbitrap measurements on 1,2,4-triazolium-based p([MVTr]I) (**13**) have been performed in negative ion mode, using a water/acetonitrile mixture (1 : 1, v/v). The mass spectrum (ranging from *m*/*z* = 500 Th to *m*/*z* = 3000 Th) of p([MVTr]I) (**12**) (Fig. 8A) shows a single charged distribution (labelled with 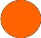), whose proposed structure is represented in Fig. 8C. The zoom (Fig. 8B) depicts the repeating unit with *m*/*z* = 236.9756 Th (*m*/*z*(theo) = 236.9763 Th). The proposed structure of p([MVTr]I) (**13**) is assigned to the saturated chain, since the unsaturated analogue expected from the disproportion reaction is far less abundant (<5%). A dominant side reaction may quench the radicals, and is responsible for both the absence of unsaturated chains and the low molecular mass observed in the full MS. In addition, a less abundant distribution (labelled with 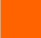) represents the double charged species. Further single charged species (labelled with 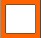 and 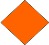) of minor abundance were detected, in which one repeating unit reacts according to the reverse Menshutkin reaction mechanism leading to demethylation and an uncharged moiety. The single charged distribution labelled with 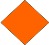 is assigned to the sodium iodide adduct. Species labelled with 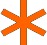 derive from (multiple) loss(es) of gaseous HI. The main structure of the polymeric chain is confirmed by the tandem MS experiment performed on the species at *m*/*z* = 906 ± 3 Th. Multiple reaction pathways were observed during the experiment (collated in Scheme S3†): the ion can undergo a demethylation, according to the reverse Menshutkin reaction, multiple losses of HI, and a stepwise loss of the entire 4-methyl-1-vinyl-1,2,4-triazolium iodide as repeating unit leading to short-chain analogues. The depolymerization mechanism was also proposed by Iván and coworkers^50^ who studied the thermal behavior of non-ionic poly(*N*-vinylimidazole) *via* thermogravimetry-mass spectrometry (TG-MS), and pyrolysis-gas chromatography/mass spectrometry (Py-GC/MS).

**Fig. 9:**
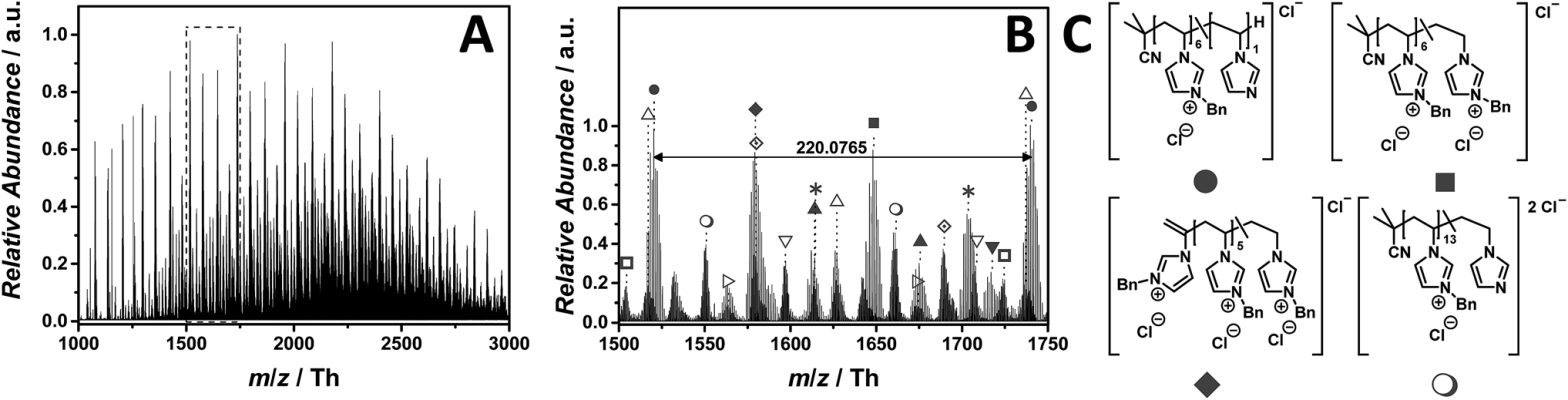
ESI-CID-Orbitrap MS spectra of p([BnVIM]Cl) (**15**) (*M*_n_ = 96 000 g mol^–1^, *Ð* = 2.55) depicting (A) overview spectrum from 1000 Th to 3000 Th; (B) zoom into one repeating unit (*m*/*z*(exp) = 220.0765 Th, *m*/*z*(theo) = 220.0767 Th); (C) proposed structure of the most abundant species. For all peak assignments refer to Table S10.† Species labelled with 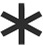 derive from (multiple) loss(es) of gaseous HCl.

The ESI-CID-Orbitrap measurements on p([BnVIM]Cl) (**15**) have been performed in negative ion mode, using a water/acetonitrile mixture (1 : 1, v/v). The ESI MS profile (Fig. 9A) ranges from *m*/*z* = 1000 to *m*/*z* = 3000 Th. As a consequence, the zoom (Fig. 9B) reveals numerous species. Since chloride is present in each repeating unit, the isotopic pattern of each peak overlaps, leading to a complex mass spectrum. Fig. 9B illustrates the most abundant single charged distribution (labelled with 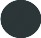), corresponding to a repeating unit of *m*/*z* = 220.0765 Th (*m*/*z*(theo) = 220.0767 Th). The proposed structure – always taking the saturated candidate as representative species – is depicted in Fig. 9C. Since the benzyl moiety is a considerable electrophile, the reverse Menshutkin reaction occurs, involving the chloride as nucleophile. The fully intact PIL is almost as abundant as the degraded one (labelled with 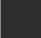). A third highly abundant single charged species is labelled with 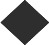 (refer to the structural determination in Table S10†). Two reaction pathways can lead to the species labelled with 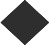. Either the structure derives from a transfer to monomer event, in which the monomer radical initiates the polymerization, or a backbiting event, in which the mid-chain radical undergoes a β-scission. Wegner and coworkers studied the homopolymerization of vinyl phosphonates where a proposed transfer to monomer event hampers the formation of high-molecular polymers in a FRP.^51^ However, since the majority of all detected species are initiated by AIBN, the β-scission after backbiting can also be taken into consideration.^52^

### PILs elucidation with ESI-QToF MS

In order to extend our structural investigation of PILs to a different mass spectrometer, we performed a parallel exploration with a Xevo G2 QToF (Waters) having a hybrid QToF mass analyzer. The reasons for using two independent mass spectrometers are manifold: (i) the QToF instrument is equipped with a Z-shaped ESI ion source (off-axis electrospray source in the case of the Orbitrap spectrometer), that allows for a completely different ionization protocol; (ii) the hybrid QToF analyzer has a wider mass range than the Orbitrap (limited to *m*/*z* = 6000 Th); (iii) QToF characterization of PILs represents a further advance in the analytical methods development for analyzing synthetic polymers. The analytical parameters of the ESI-QToF instrument were adjusted according to the MassLynx software, by varying the sample cone voltage for each PIL individually between 25 to 50 V and setting the capillary voltage to 2.5 kV. As reported by Derrick and co-workers,^53^ the sampling cone voltage is a key parameter of the ion source. The tuning of this parameter significantly affects the single or multiple charged distributions of synthetic polymers. Due to the collisional activation of the ions by ubiquitary neutral gas molecules associated with a declustering effect, relative changes in intensities of such multiple charged distributions are observed.^54^ In addition, Z-shaped trajectory is an important feature that influences the ionization process of PILs. As a result of the synergy of the two mentioned aspects, we were able to efficiently analyze the synthesized PILs without any supercharging agent, observing the same charge state distribution of the polymers as obtained *via* ESI-CID-Orbitrap MS. The ESI-QToF measurements were performed with negative polarity in the sensitivity mode. The polymers were dissolved in a water/acetonitrile mixture (1 : 1, v/v). Despite the lower resolution, the ESI-QToF spectra enabled the entire structural determination of all PILs and their potential by-products (Fig. S51†). P([TPVBP]Cl) (**8**) was successfully characterized *via* ESI-QToF MS, setting the sample cone voltage to 25 V. The ESI MS profile (Fig. S51†) reveals various double charged distributions. The zoom region between *m*/*z* = 2560 and *m*/*z* = 3000 Th depicts the most abundant distribution (labelled with 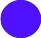) and one repeating unit with *m*/*z* = 207.0671 Th (*m*/*z*(theo) = 207.0658 Th). A further double charged distribution labelled with 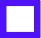 was assigned to the PIL having one hydrolyzed repeating unit, due to the electrophilic nature of the phosphonium group. Both distributions are equally abundant as revealed by the ESI-CID-Orbitrap spectrum of the p([TPVBP]Cl) (**8**). The single charged peak (labelled with 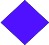) is the ion at *m*/*z* = 2869.9646 Th (*m*/*z*(theo) = 2869.9152 Th). Less abundant distributions (labelled with 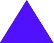, 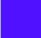, and 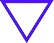) were attributed to double charged species having two, three and four hydrolyzed repeating units, respectively. Species labelled with 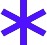 derive from multiple losses of hydrochloric acid. All charged species revealed by the ESI-CID-Orbitrap spectrum of the p([TPVBP]Cl) (**8**) were detected by the ESI-QToF spectrometer. In addition, further distributions arising from multiple hydrolyses of the phosphonium group along the polymer chain were observed. Therefore, the ESI-QToF MS measurements represent a further analytical protocol for characterizing PILs as well as a valuable confirmation of the structural investigation of this class of polyelectrolytes carried out with the ESI-CID-Orbitrap technique.

## Conclusions

We introduce a universal mass spectrometric platform for the complete and detailed structural elucidation of poly(ionic liquid)s efficiently synthesized *via* RAFT polymerization and non-controlled free radical polymerization strategies. ESI-CID-Orbitrap MS in combination with a supercharging agent as well as ESI-QToF MS are demonstrated to be powerful characterization access modes to structural information of complex polyelectrolytes. In addition, the structures of the investigated PILs have been explored by tandem mass spectrometry experiments. Our novel ESI MS protocol provides – for the first time – general access to electrolyte-type polymers and constitutes a technology platform for their analysis. In addition, we demonstrate that the analysis of complex synthetic polymers can profit significantly from the most recent developments in the mass spectrometric analysis of biomolecules.

## Supplementary Material

Supplementary information
