# Supplementary material for: Universal mass spectrometric analysis of poly(ionic liquid)s
Source: Chem Sci. 2016 Apr 21;7(8):4912–21. doi: 10.1039/c6sc01347c (PMC6018439; doi:10.1039/c6sc01347c)
Supplement: Supplementary file 1 [file SC-007-C6SC01347C-s001.pdf]

Electronic Supplementary Material (ESI) for Chemical Science.
This journal is © The Royal Society of Chemistry 2016

Supporting Information

# Universal Mass Spectrometric Analysis of Poly(Ionic Liquid)s

Martina M. Cecchini,[‡,a] Jan Steinkoenig,[‡,b,c] Samantha Reale,[a] Leonie Barner,[d] Jiayin Yuan,[e] Anja S. Goldmann,[b,c] Francesco De Angelis,*[a] Christopher Barner-Kowollik*[b,c,d]

[a] Dipartimento di Scienze Fisiche e Chimiche, Università degli Studi dell'Aquila, Via Vetoio, Coppito, 67100, L'Aquila, Italy.

[b] Preparative Macromolecular Chemistry, Institut für Technische Chemie und Polymerchemie, Karlsruhe Institute of Technology (KIT), Engesserstr. 18, 76128 Karlsruhe, Germany

[c] Institut für Biologische Grenzflächen, Karlsruhe Institute of Technology (KIT), Hermann-von-Helmholtz-Platz 1, 76344 Eggenstein-Leopoldshafen, Germany.

[d] Soft Matter Synthesis Laboratory, Institut für Biologische Grenzflächen, Karlsruhe Institute of Technology (KIT), Hermann-von-Helmholtz-Platz 1, 76344 Eggenstein-Leopoldshafen, Germany.

[e] Max-Planck-Institute of Colloids and Interfaces, Research Campus Golm, 14424 Potsdam, Germany.

‡ Both authors contributed equally to this study.

## Materials

All solvents for synthesis were obtained from Sigma-Aldrich, Acros Organics or Fischer and used without further purification. Absolute solvents were purchased from Acros Organics and stored under nitrogen and over molecular sieves. 2,2′-Azobis(2-methylpropionitril) (AIBN) was recrystallized twice from methanol. 2-(acryloyloxy)-*N*,*N*,*N*-trimethylethan-1-aminium chloride (**9**) was purchased from Sigma-Aldrich (80% water solution) and purified by two times precipitation in acetone, dried under high-vacuum and stored at -5 °C. All other reactants were used without further purification. The dialysis tubes were purchased from Spectra/Por (Spectra/Por 7 Dialysis RC Tubing).

All solvents for mass spectrometric analysis (Milli-Q water; UHPCL-grade acetonitrile (Thermo Fisher)) were used without further treatment.

1-(chloromethyl)-4-vinylbenzene (vinylbenzyl chloride) (90%, Sigma-Aldrich), 2-cyano-2-propyl dodecyl trithiocarbonate (97%, Sigma-Aldrich), 1,1'-azobiscyclohexanecarbonitrile (VAZO-88) (98%, Sigma-Aldrich), 1-butylimidazole (98%, Sigma-Aldrich), 1-decyl-2-methylimidazole (98%, Sigma-Aldrich), pyridine (>99%, Acros), trimethylamine (>99%, Merck), triphenylphosphine (>99%, Merck), propylene carbonate (98%, TCI) were used as received.

## Characterization Methods

**Electrospray ionization-Orbitrap mass spectrometry.** Mass spectra were recorded on a Q Exactive (Orbitrap) mass spectrometer (Thermo Fisher Scientific, San Jose, CA, USA) equipped with a HESI II probe. The instrument was calibrated in the *m*/*z* range 1000-6000 Th using ammonium hexafluorophosphate (Thermo Scientific). All spectra were recorded in the negative mode, using water/acetonitrile (1:1, *v*/*v*) in a concentration of 0.5 mg·mL$^{-1}$ as solvent. The FT resolution was set to 140 000 employing 3 microscans during an acquisition time between 2 and 3 min measuring with a capillary temperature of 320 °C. The aux gas flow was (dimensionless) 0.00, the sheath gas 10.00, and the spare gas 1.00. The flow rate was set to 10 µL·min$^{-1}$. The spray voltage was set between 2.0 eV and 4.5 eV depending on the *S*/*N* ratio. A CID fragmentation was employed depending on the analyzed PIL. The ion optics settings are as follows: the S-lens RF level was set to 68.0, the S-lens voltage to -25.0 V, the skimmer voltage to -15.0 V, the gate lens voltage to -6.00 V and the C-trap RF to 2400.0 V.

**Electrospray ionization-QToF mass spectrometry.** Electrospray ionization mass spectrometry measurements were performed on a Quadrupole Time-of-Flight mass spectrometer instrument (QToF) (Xevo® G2 QToF; Waters Corporation, Milford, USA), equipped with a high performance ZSpray™ source and operating with MassLynx™ Software package (Version 4.1, Waters Corporation). The instrument was calibrated using a 5 mM sodium formate solution in 90:10 2-propanol:water, in the *m*/*z* range 100-5000 Th. The spectra were recorded in negative polarity choosing the sensitivity mode,

according to the MassLynx™ software; the *m/z* range 1000-5000 Th was set to detect the polymeric distributions. The following setting parameters have been used: source temperature at 120 °C; desolvation temperature at 280 °C; capillary voltage of 2.5 kV; the cone voltage was adjusted, according to the MassLynx™ software, to 30 and 50 V and the extraction cone to 2.0 and 4.0 V. The collision energy was 6 eV. The desolvation gas flow rate was 800 $L \cdot h^{-1}$. Confirmation of the ion structure was obtained from exact mass calculations, isotopic simulations and tandem mass spectrometry experiments.

**Size exclusion chromatography (SEC)**. Water-based SEC was performed on a PSS WinGPC, comprising an autosampler, a PSS Novema Max precolumn, one PSS Novema Max (particle size 10 µm, dimension 8.00 × 300.00 mm, porosity 30 Å), two PSS Novema Max (particle size 10 µm, dimension 8.00 × 300.00 mm, porosity 1000 Å), and a differential refractive index detector (PSS SECcurity RI) as well as an UV detector (PSS SECcurity UV) using water, 0.5 $g \cdot L^{-1}$ NaCl and 0.3 M formic acid as the eluent at 30 °C with a flow rate of 1 $mL \cdot min^{-1}$. The SEC system was calibrated using linear poly(2-vinyl pyridine) standards ranging from 1100 to $1.06 \cdot 10^6$ $g \cdot mol^{-1}$. SEC calibration was carried out relative to poly(2-vinyl pyridine) calibrations (Mark Houwink parameters $K = 2.5 \cdot 10^{-5}$ $dL \cdot g^{-1}$; $\alpha = 0.93$).

**Nuclear magnetic resonance (NMR) spectroscopy.** Proton nuclear magnetic resonance ($^1H$ NMR) spectra were recorded on a Bruker AM 400 (400 MHz) spectrometer. Chemical shifts are expressed in parts per million (ppm) and calibrated on characteristic solvent signals as internal standards. All coupling constants are absolute values and *J* values are expressed in Hertz (Hz).The description of signals include: s = singlet, bs = broad singlet, d = doublet, dd = double doublet, t = triplet, q = quartet, m = multiplet. Carbon nuclear magnetic resonance ($^{13}C\{^1H\}$ NMR) spectra were recorded on a Bruker AM 400 (100 MHz) spectrometer. Phosphorus nuclear magnetic resonance ($^{31}P\{^1H\}$ NMR) spectra were recorded on a Bruker AM 400 (162 MHz) spectrometer.

## Experimental Part

**Synthesis of 1-decyl-2-methyl-3-(4-vinylbenzyl)-1*H*-imidazol-3-ium chloride ([DeMVBIM]Cl, 1).** A Schlenk tube was charged with 4.50 g 1-decyl-2-methylimidazole (20.2 mmol, 1.00 eq), dissolved in 100 mL $CHCl_3$ and cooled to 0 °C. Subsequently, 4.29 mL vinylbenzyl chloride (30.4 mmol, 1.50 eq) was added and the tube was heated from 0 °C to 50 °C. The mixture was stirred at 50 °C for 24 h. The mixture was extracted with water (1×80 mL) (phase separation overnight), evaporated at reduced pressure to get rid of the residual organic solvents, and freeze-dried. 3.27 g of the product (58%) was received as a colorless liquid.

$^1$H NMR (400 MHz, DMSO-d$_6$, 298 K): $\delta$ = 7.82 (d, $^3J$ = 2.1 Hz, 1H), 7.79 (d, $^3J$ = 2.1 Hz, 1H), 7.51 (d, $^3J$ = 8.1 Hz, 2H), 7.31 (d, $^3J$ = 8.1 Hz, 2H), 6.73 (dd, $^3J$(E) = 17.7 Hz, $^3J$(E) = 11.0 Hz, 1H), 5.86 (d, $^3J$ = 17.7 Hz, 1H), 5.43 (s, 2H), 5.29 (d, $^3J$ = 11.0 Hz, 1H), 4.12 (t, $^3J$ = 7.4 Hz, 3H), 2.63 (s, 3H), 1.78 – 1.62 (m, 2H), 1.32 – 1.15 (m, 14H), 0.85 (t, $^3J$ = 6.8 Hz, 4H) ppm.

$^{13}$C{$^1$H} NMR (100 MHz, DMSO-d$_6$, 298 K): $\delta$ = 144.02 (C), 137.28 (CH), 135.92 (C), 134.07 (C), 128.12 (CH), 126.64 (CH), 121.72 (CH), 121.57 (CH), 115.13 ($CH_2$), 50.32 ($CH_2$), 47.63 ($CH_2$), 31.24 ($CH_2$), 28.94 ($CH_2$), 28.84 ($CH_2$), 28.64 ($CH_2$), 28.43 ($CH_2$), 25.58 ($CH_2$), 22.07 ($CH_2$), 13.93 ($CH_3$), 9.50 ($CH_3$) ppm.

**Synthesis of 1-(4-vinylbenzyl)pyridin-1-ium chloride ([VBPy]Cl, 2).** A Schlenk tube was charged with 2.48 mL pyridine (8.00 g, 52.4 mmol, 1.00 eq), dissolved in 100 mL $CHCl_3$ and cooled to 0 °C. Subsequently, 7.41 mL vinylbenzyl chloride (2.44 g, 30.8 mmol, 1.50 eq) was added and the tube was heated from 0 °C to 50 °C. The mixture was stirred at 50 °C for 24 h. The mixture was extracted with water (3×50 mL), evaporated at reduced pressure to get rid of the residual organic solvents, and freeze-dried. 4.81 g of the product (40%) were obtained as a colorless, highly viscous liquid.

$^1$H NMR (400 MHz, DMSO-d$_6$, 298 K): $\delta$ = 9.37 (d, $^3J$ = 5.5 Hz, 2H), 8.68 – 8.59 (m, 1H), 8.23 – 8.15 (m, 2H), 7.59 (d, $^3J$ = 8.3 Hz, 2H), 7.53 (d, $^3J$ = 8.3 Hz, 2H), 6.73 (dd, $^3J$(E) = 17.7, $^3J$(Z) = 11.0 Hz, 1H), 5.96 (s, 1H), 5.88 (d, $^3J$ = 17.7, 1H), 5.30 (d, $^3J$ = 11.0, 1H) ppm.

$^{13}$C{$^1$H} NMR (100 MHz, DMSO-d$_6$, 298 K): $\delta$ = 145.94 (CH), 144.87 (CH), 138.09 (C), 135.82 (C), 133.83 (CH), 129.30 (CH), 128.43 (CH), 126.82 (CH), 115.68 ($CH_2$), 62.69 ($CH_2$) ppm.

**Synthesis of *N*,*N*,*N*-triethyl-*N*-(4-vinylbenzyl)ammonium chloride ([TEVBA]Cl, 3).** A Schlenk tube was charged with 3.50 g triethylamine (34.6 mmol, 1.00 eq), dissolved in 50 mL $CHCl_3$ and cooled to 0 °C. Subsequently, 7.33 mL vinylbenzyl chloride (7.92 g, 51.9 mmol, 1.50 eq)

was added and the tube was heated from 0 °C to 50 °C. The mixture was stirred at 50 °C for 24 h. The mixture was extracted with water (3×40 mL), evaporated at reduced pressure to get rid of the residual organic solvents, and freeze-dried. 8.22 g of the product (94%) were obtained as colorless needles.

$^{1}$H NMR (400 MHz, DMSO-d$_6$, 298 K): $\delta$ = 7.59 (d, $^{3}J$ = 8.2 Hz, 2H), 7.52 (d, $^{3}J$ = 8.2 Hz, 2H), 6.79 (dd, $^{3}J$(E) = 17.7 Hz, $^{3}J$(Z) = 11.0 Hz, 1H), 5.95 (d, $^{3}J$ = 17.7 Hz, 1H), 5.37 (d, $^{3}J$ = 11.0 Hz, 1H), 4.54 (s, 2H), 3.19 (q, $^{3}J$ = 7.1 Hz, 6H), 1.30 (t, $^{3}J$ = 7.1 Hz, 9H) ppm.

$^{13}$C{$^{1}$H} NMR (100 MHz, DMSO-d$_6$, 298 K): $\delta$ = 138.71 (CH), 135.76 (C), 132.88 (CH), 127.38 (C), 126.54 (CH), 116.18 ($CH_2$), 59.27 ($CH_2$), 52.01 ($CH_2$), 7.59 ($CH_3$) ppm.

**Synthesis of triphenyl(4-vinylbenzyl)phosphonium chloride ([TPVBP]Cl, 4).** 5.00 g vinylbenzyl chloride (29.5 mmol, 1.50 eq) was added dropwise to a stirred solution of 7.73 g triphenylphosphine (29.5 mmol, 1.00 eq) in 20 mL acetonitrile. Subsequently, the homogeneous solution was stirred at 50 °C for 24 hours. The reaction mixture was cooled to ambient temperature and the solvent removed under reduced pressure to afford a white solid. The product was thoroughly washed by stirring with three 30 mL portions of acetone. The solid was finally washed with 30 mL of diethyl ether and dried under vacuum overnight.

$^{1}$H NMR (400 MHz, DMSO-d$_6$, 298 K): $\delta$ = 7.95 – 7.86 (m, 3H), 7.80 – 7.67 (m, 12H), 7.33 (d, $^{3}J$ = 8.1 Hz, 2H), 6.98 (d, $^{3}J$ = 8.  Hz, 2H), 6.65 (dd, $^{3}J$(E) = 17.6 Hz, $^{3}J$(Z) = 11.0 Hz, 1H), 5.81 (d, $^{3}J$ = 17.6 Hz, 1H), 5.30 (d, $^{2}J$ = 15.9 Hz, 2H), 5.25 (d, $^{3}J$ = 11.0 Hz, 1H) ppm.

$^{13}$C{$^{1}$H} NMR (100 MHz, DMSO-d$_6$, 298 K): $\delta$ = 303.70 (d, $^{2}J$ = 4.2 Hz, C), 302.50 (C), 301.78 (d, $^{4}J$ = 2.7 Hz, CH), 300.79 (d, $^{3}J$ = 9.8 Hz, CH), 297.85 (d, $^{3}J$ = 5.7 Hz, CH), 296.80 (d, $^{2}J$ = 12.4 Hz, CH), 294.19 (CH), 293.11 (d, $^{4}J$ = 3.3 Hz, CH), 284.61 (d, $^{1}J$ = 85.5 Hz, C), 281.97 ($CH_2$), 194.70 (d, $^{1}J$ = 46.4 Hz, $CH_2$) ppm.

$^{31}$P{$^{1}$H} NMR (162 MHz, DMSO-d$_6$, 298 K): $\delta$ = 22.83 ppm.

**Synthesis of 1-butyl-3-(4-vinylbenzyl)-1*H*-imidazol-3-ium chloride ([BVBIM]Cl).** The synthesis of [BVBIM]Cl is described in literature.[1]

**Synthesis of DoPAT-PE.** The synthesis of the photo-enol RAFT agent is described in literature.[2]

**Synthesis of 4-methyl-1-vinyl-4*H*-1,2,4-triazol-1-ium iodide ([MVTr]I, 12).** A 100 mL flask was filled with a mixture of 5.40 mL 1-vinyl-1,2,4-triazole 1 (5.50 g, 57.8 mmol, 1.00 eq), 5.40 mL iodomethane (12.3 g, 86.7 mmol, 1.50 eq) and 50 mg 2,6-di-tert-butyl-4-methylphenol

(0.227 mol). After stirring and heating for 24 h at 50 °C, the crude product was filtered off and washed with THF for three times. A pale yellow solid (13.21 g, 96.4%) was obtained.
$^1$H NMR (400 MHz, DMSO-$d_6$, 298 K): $\delta$ = 10.35 (s, 1H), 9.28 (s, 1H), 7.54 (dd, $^3J$(E) = 15.3 Hz, $^3J$(Z) = 8.6 Hz, 1H), 6.03 (d, $^3J$(E) =15.3 Hz, 1H), 5.56 (d, $^3J$(Z) =8.6 Hz, 1H), 3.93 (s, 3H) ppm.
$^{13}$C{$^1$H} NMR (100 MHz, DMSO-$d_6$, 298 K): $\delta$ = 145.38 (CH), 142.03 (CH), 128.98 (CH), 110.21 ($CH_2$), 34.70 ($CH_3$) ppm.

**Synthesis of 1-benzyl-3-vinyl-1*H*-imidazol-3-ium chloride ([BnVIM]Cl, 14).** A mixture of 9.41 g 1-vinyl-imidazole (0.100 mol, 1.00 eq), 12.7 g benzyl chloride (0.100 mol, 1.00 eq) and 20 mL of ethanol with 100 mg 2,6-di-tert-butyl-4-methylphenol as the stabilizer were charged into a 100 mL Schlenk flask. The mixture was stirred at ambient temperature for 1 h and at 35 °C for another hour before it was kept at 60 °C for 24 h. After cooling down to ambient temperature, the mixture was poured into 1 L THF. The liquid oily phase was washed with THF 3 times and dried at ambient temperature under high vacuum for 4 h. A pale yellow product (17.7 g, 80%) was then stored in a freezer.
$^1$H NMR (400 MHz, $D_2O$, 298 K): $\delta$ = 9.06 (s, 1H), 7.70 (s, 1H), 7.46 (s, 1H), 7.44 – 7.26 (m, 5H), 7.05 (dd, $^3J$(E) = 15.6 Hz, $^3J$(Z) = 8.7 Hz, 1H), 5.73 (d, $^3J$(E) = 15.6 Hz, 1H), 5.37 (d, $^3J$(Z) = 8.7 Hz, 1H), 5.31 (s, 2H) ppm.
$^{13}$C{$^1$H} NMR (100 MHz, $D_2O$, 298 K): $\delta$ = 134.13 (CH), 133.01 (CH), 129.30 (CH), 129.28 (CH), 128.74 (CH), 127.93 (CH), 122.66 (CH), 119.52 (CH), 109.48 ($CH_2$), 53.02 ($CH_2$) ppm.

**Synthesis of poly(1-decyl-2-methyl-3-(4-vinylbenzyl)-1*H*-imidazol-3-ium chloride) (p([DeMVBIM]Cl), 5).** A Schlenk flask was charged with 3.85 g 1-decyl-2-methyl-3-(4-vinylbenzyl)-1H-imidazol-3-ium chloride (**1**) (10.26 mmol, 200.00 eq), 2.51 mg 1,1′-azobis(cyclohexanecarbonitrile) (0.0103 mmol, 0.20 eq) and 17.7 mg 2-cyano-2-propyldodecyl trithiocarbonate (0.0513 mmol, 1.00 eq). The mixture was dissolved in 20 mL DMF/EtOH (1:1, *v*/*v*), degassed by three consecutive freeze-pump-thaw cycles, filled with nitrogen and subsequently immersed in a preheated oil bath at 85 °C. The polymerization was stopped by sudden freezing in liquid nitrogen, diluted with water and purged with air. The polymer was purified by dialysis in pure water (3 d, a.t., 1 kDa MWCO).

**Synthesis of poly(1-(4-vinylbenzyl)pyridin-1-ium chloride) (p([VBPy]Cl), 6).** A Schlenk flask was charged with 4.81 g 1-(4-vinylbenzyl)pyridin-1-ium chloride (**2**) (20.8 mmol, 200.00 eq.), 5.1 mg 1,1′-azobis(cyclohexanecarbonitrile) (0.0208 mmol, 0.20 eq.) and 36.3 mg 2-cyano-2-

propyldodecyl trithiocarbonate (0.105 mmol, 1.00 eq.). The mixture was dissolved in 48 mL DMSO and 24 mL DMF, degassed by three consecutive freeze–pump–thaw cycles, filled with nitrogen, and subsequently immersed in a preheated oil bath at 85 °C. The polymerization was stopped by sudden freezing in liquid nitrogen, diluted with water and purged with air. The polymer was purified by dialysis in pure water (3 d, a.t., 1 kDa MWCO).

**Synthesis of poly(*N*,*N*,*N*-triethyl-*N*-(4-vinylbenzyl)ammonium chloride) (p([TEVBA]Cl), 7).** A Schlenk flask was charged with 2.70 g *N*,*N*,*N*-triethyl-*N*-(4-vinylbenzyl)ammonium chloride (**3**) (63.1 mmol, 200.00 eq), 1.86 mg 1,1′-azobis(cyclohexanecarbonitrile) (0.0076 mmol, 0.20 eq) and 13.1 mg 2-cyano-2-propyldodecyl trithiocarbonate (0.0380 mmol, 1.00 eq). The mixture was dissolved in DMSO (15 mL) and DMF (10 mL), degassed by three consecutive freeze-pump-thaw cycles, filled with nitrogen and subsequently immersed in a preheated oil bath at 85 °C. The polymerization was stopped by sudden freezing in liquid nitrogen, diluted with water and purged with air. The polymer was purified by dialysis in pure water (3 d, a.t., 1 kDa MWCO).

**Synthesis of poly(triphenyl(4-vinylbenzyl)phosphonium chloride) (p([TPVBP]Cl), 8).** A Schlenk flask was charged with 7.10 g triphenyl(4-vinylbenzyl)phosphonium chloride (**4**) (17.1 mmol, 200.00 eq), 4.2 mg 1,1′-azobis(cyclohexanecarbonitrile) (0.017 mmol, 0.20 eq) and 29.6 mg 2-cyano-2-propyldodecyl trithiocarbonate (0.086 mmol, 1.00 eq). The mixture was dissolved in DMSO (15 mL) and DMF (10 mL), degassed by three consecutive freeze-pump-thaw cycles, filled with nitrogen and subsequently immersed in a preheated oil bath at 85 °C. The polymerization was stopped by sudden freezing in liquid nitrogen, diluted with water and purged with air. The polymer was purified by dialysis in pure water (3 d, a.t., 1 kDa MWCO).

**Synthesis of poly(2-(acryloyloxy)-*N*,*N*,*N*-trimethylethan-1-aminium chloride) (p([ATMEA]Cl), 10).** A Schlenk flask was charged with 4.00 g 2-(acryloyloxy)-*N*,*N*,*N*-trimethylethan-1-aminium chloride (**9**) (20.7 mmol, 200.00 eq), 3.4 mg 2,2′-azobis(2-methylpropionitril) (0.0207 mmol, 0.20 eq) and 35.8 mg 2-cyano-2-propyldodecyl trithiocarbonate (0.104 mmol, 1.00 eq). The mixture was dissolved in 20 mL DMSO/$H_2O$ (1:1, *v*/*v*), degassed by three consecutive freeze-pump-thaw cycles, filled with nitrogen and subsequently immersed in a preheated oil bath at 60 °C. The polymerization was stopped by sudden freezing in liquid nitrogen, diluted with water and purged with air. The polymer was purified by dialysis in pure water (3 d, a.t., 1 kDa MWCO).

**Synthesis of poly(1-butyl-3-(4-vinylbenzyl)-1*H*-imidazol-3-ium chloride) (p([BVBIM]Cl), 11).** A Schlenk flask was charged with 13.9 g 1-butyl-3-(4-vinylbenzyl)-1*H*-imidazol-3-ium chloride (50.3 mmol, 200 eq), 12.3 mg 1,1'-azobis(cyclohexanecarbonitrile) (0.0503 mmol, 0.20 eq) and 166 mg DoPAT-PE (251 mmol, 1.00 eq). The mixture was dissolved in 75 mL DMSO/DMF (1:1, *v*/*v*), degassed by three consecutive freeze-pump-thaw cycles, filled with nitrogen and subsequently immersed in a preheated oil bath at 85 °C. The polymerization was stopped by sudden freezing in liquid nitrogen, diluted with water and purged with air. The polymer was purified by dialysis in pure water (3 d, a.t., 1 kDa MWCO).

**Synthesis of poly(4-methyl-1-vinyl-4*H*-1,2,4-triazol-1-ium iodide) (p([MVTr]I), 13).** A mixture of 5.00 g 4-methyl-1-vinyl-4*H*-1,2,4-triazol-1-ium iodide **(12)** (21.10 mmol), 50 mg AIBN (0.300 mmol), and 20 mL of anhydrous DMF was put inside a 50 mL Schlenk flask under argon protection. Three freeze-pump-thaw cycles were applied for oxygen removal. The reaction was stirred at 75 °C for 24 h and the crude product was dialyzed in water (2 d, a.t.). A yellow powder was received after removal of solvents, the yield was 3.6 g (72%).

**Synthesis of poly(1-benzyl-3-vinyl-1*H*-imidazol-3-ium chloride) (p([BnVIM]Cl), 15).** A 100 mL Schlenk flask was filled with a mixture of 5.00 g 1-benzyl-3-vinyl-1*H*-imidazol-3-ium chloride **(14).** (22.6 mol), 50 mg AIBN (0.300 mmol), and 50 mL DMSO. Then, three freeze-pump-thaw cycles were applied for oxygen removal and argon protection. The reaction was stirred at 90 °C for 12 h. After cooling down to ambient temperature, the crude product was precipitated in THF. The precipitate was re-dissolved in methanol and precipitated in THF again. 4.00 g of a white powder (80%) was received after removal of solvents.

**NMR data**

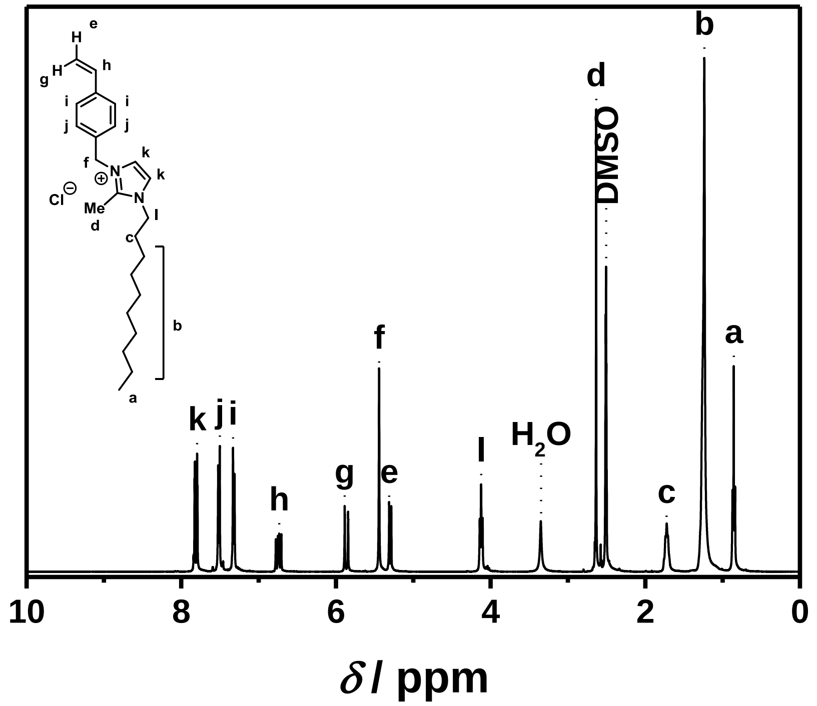


**Figure S1** $^{1}H$ NMR (400 MHz, 298 K) of [DeMVBIM]Cl (**1**) in DMSO-$d_6$.

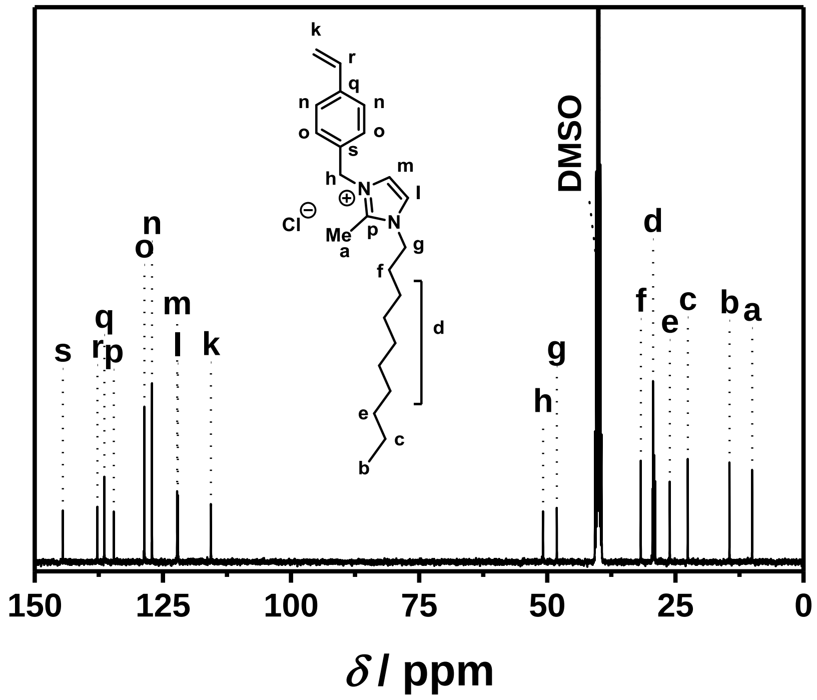


**Figure S2** $^{13}C\{^{1}H\}$ NMR (100 MHz, 298 K) of [DeMVBIM]Cl (**1**) in DMSO-$d_6$.

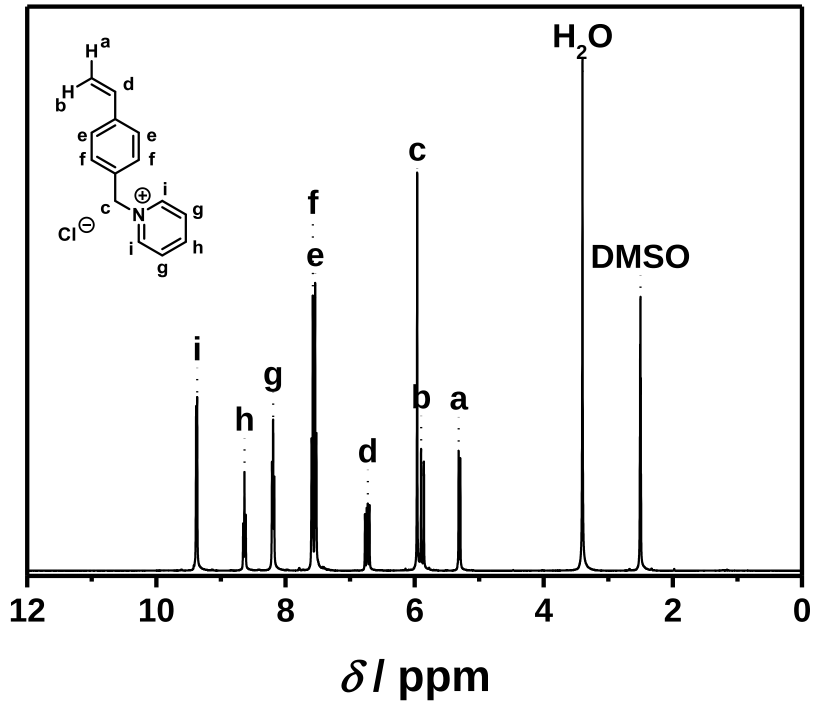


**Figure S3** $^{1}H$ NMR (400 MHz, 298 K) of [VBPy]Cl (**2**) in DMSO-$d_6$.

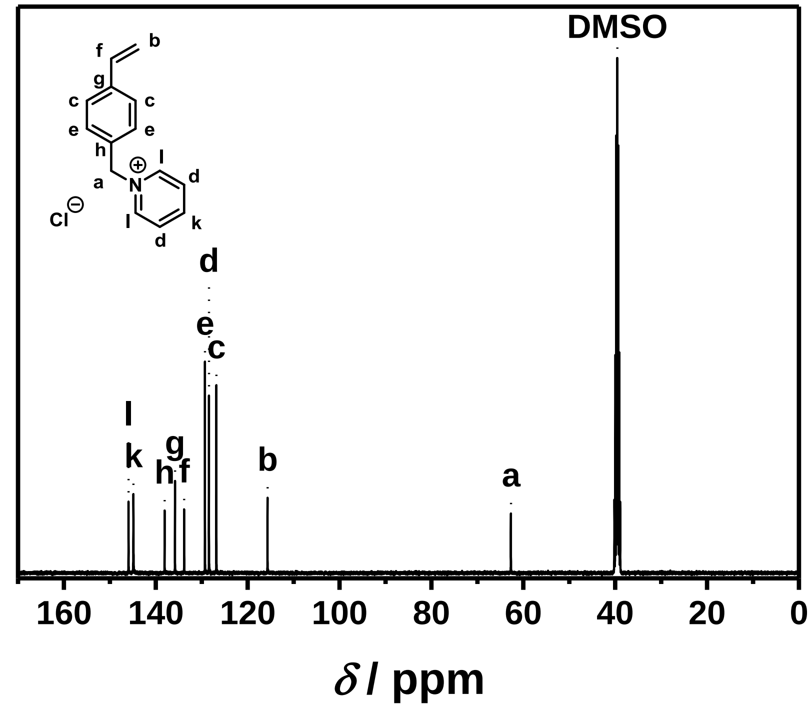


**Figure S4** $^{13}C\{^{1}H\}$ NMR (100 MHz, 298 K) of [VBPy]Cl (**2**) in DMSO-$d_6$.

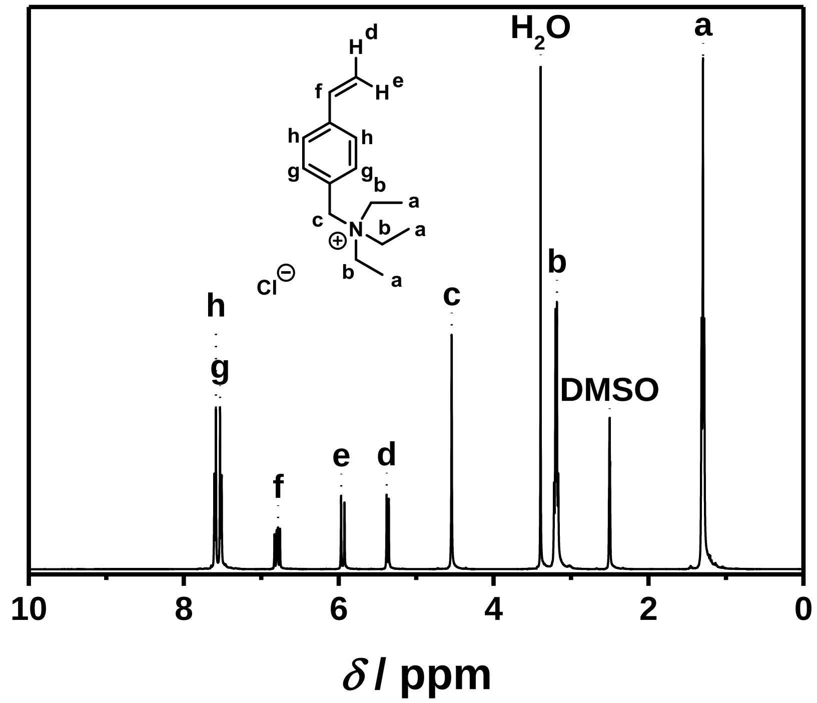


**Figure S5** $^{1}$H NMR (400 MHz, 298 K) of [TEVBA]Cl (**3**) in DMSO-$d_6$.

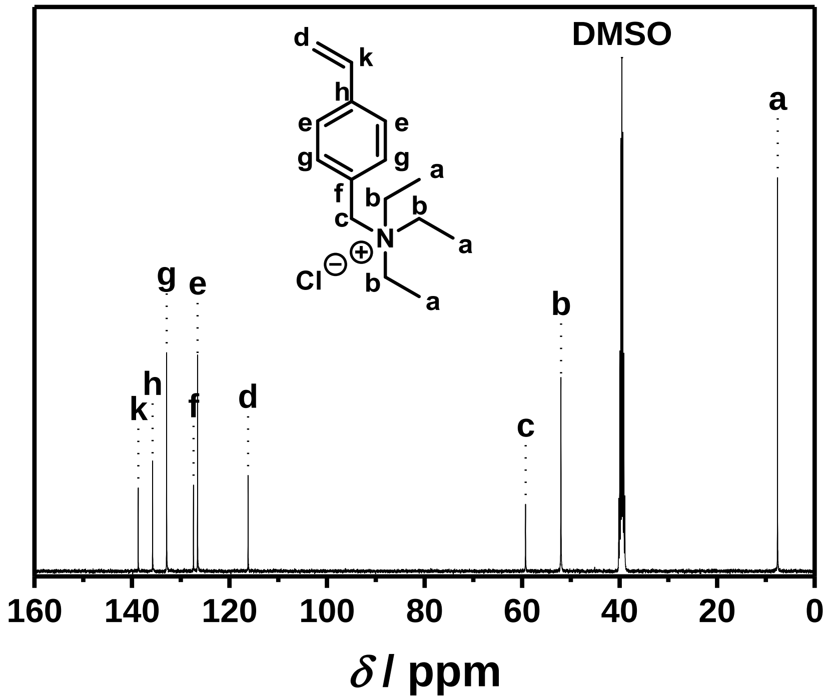


**Figure S6** $^{13}$C{$^{1}$H} NMR (100 MHz, 298 K) of [TEVBA]Cl (**3**) in DMSO-$d_6$.

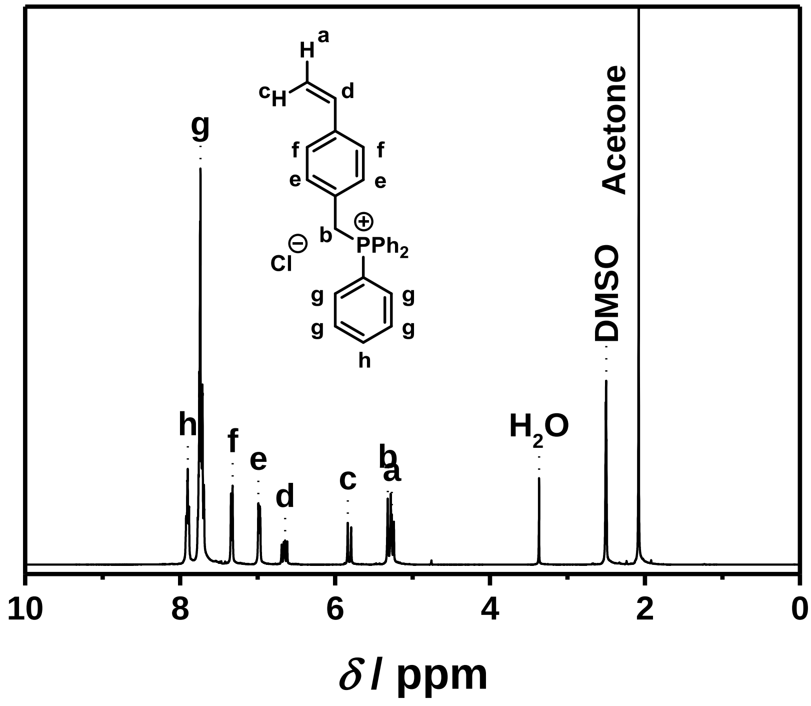


**Figure S7** $^{1}$H NMR (400 MHz, 298 K) of [TPVBP]Cl (**4**) in DMSO-d$_6$.

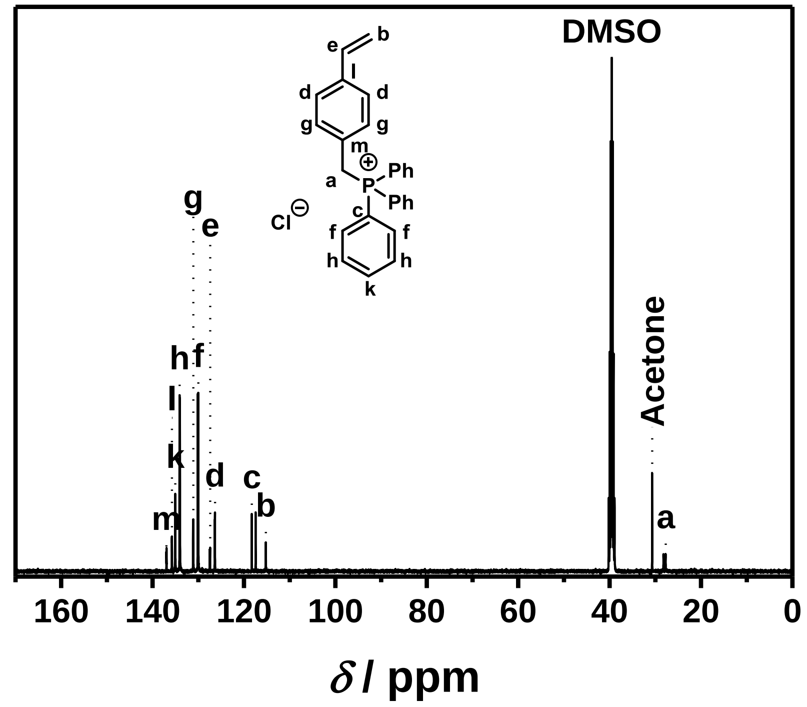


**Figure S8** $^{13}$C{$^{1}$H} NMR (100 MHz, 298 K) of [TPVBP]Cl (**4**) in DMSO-d$_6$.

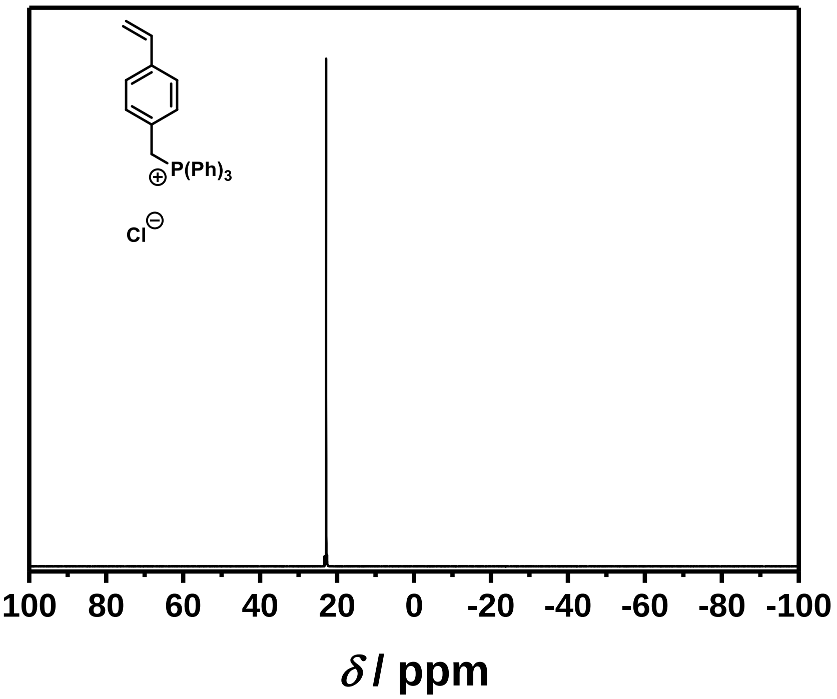


**Figure S9** $^{31}P\{^{1}H\}$ NMR (162 MHz, 298 K) of [TPVBP]Cl (**4**) in DMSO-d$_6$.

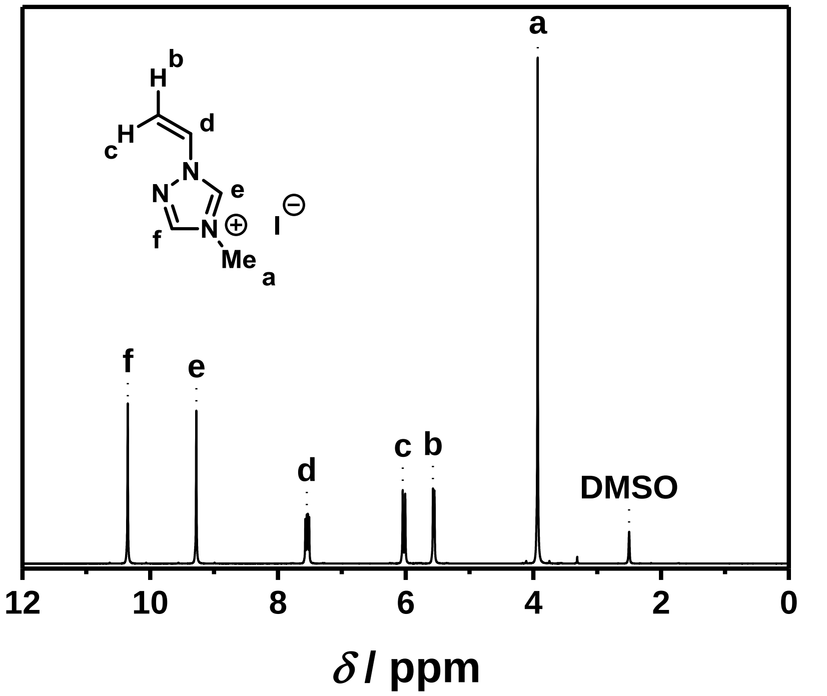


**Figure S10** $^{1}H$ NMR (400 MHz, 298 K) of [MVTr]I (**12**) in DMSO-d$_6$.

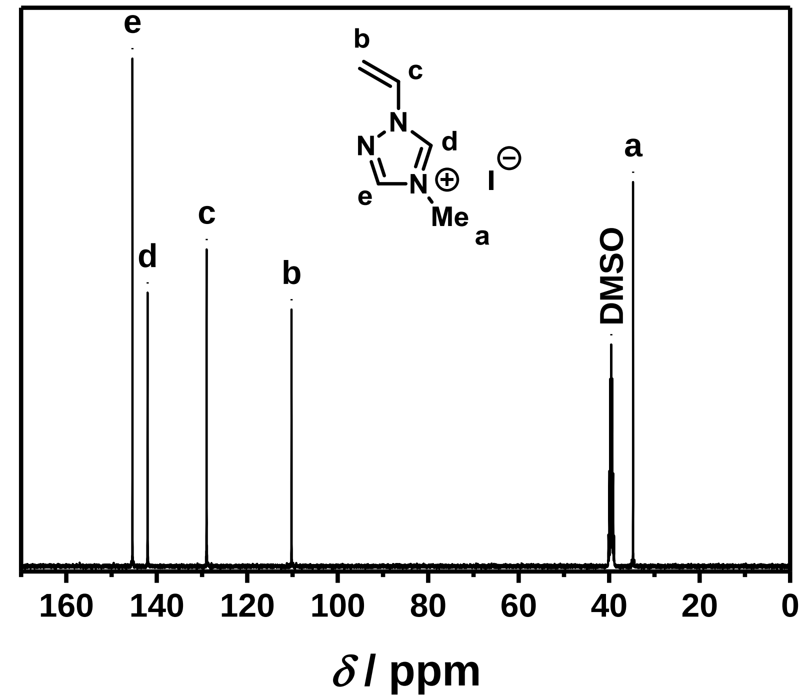


**Figure S11** $^{13}C\{^1H\}$ NMR (100 MHz, 298 K) of [MVTr]I (**12**) in DMSO-d$_6$.

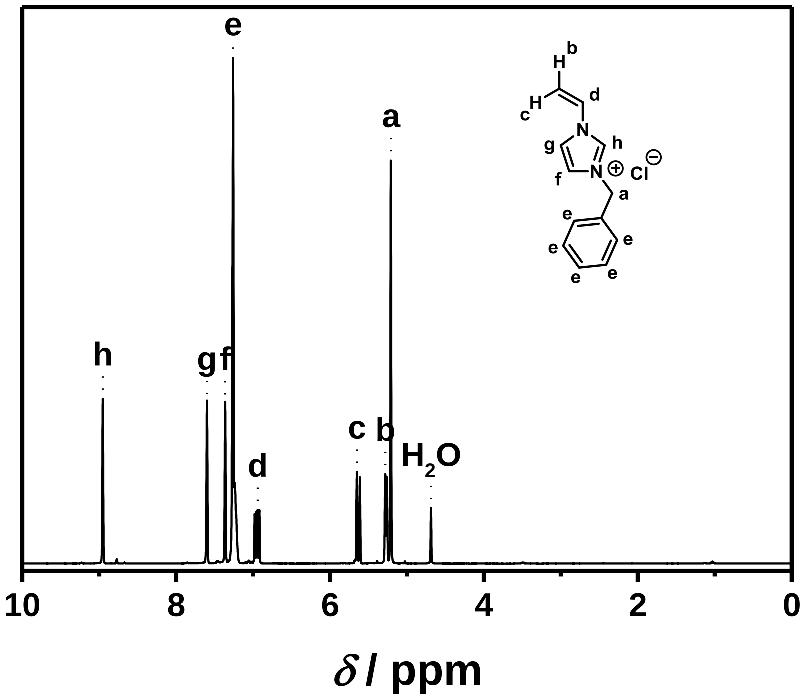


**Figure S12** $^1H$ NMR (400 MHz, 298 K) of [BnVIM]Cl (**14**) in $D_2O$.

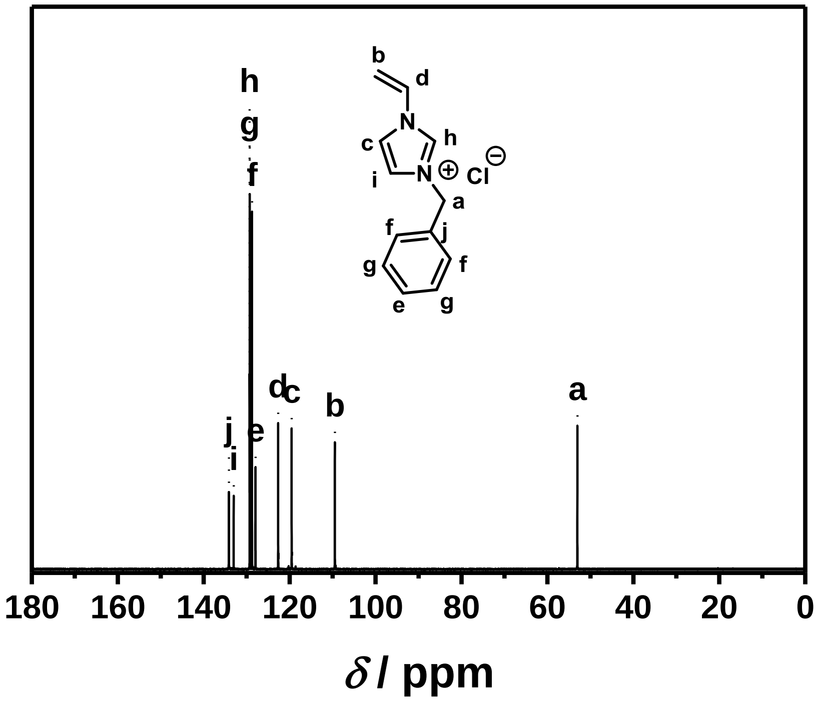


**Figure S13** $^{13}C\{^{1}H\}$ NMR (100 MHz, 298 K) of [BnVIM]Cl (**14**) in $D_2O$.

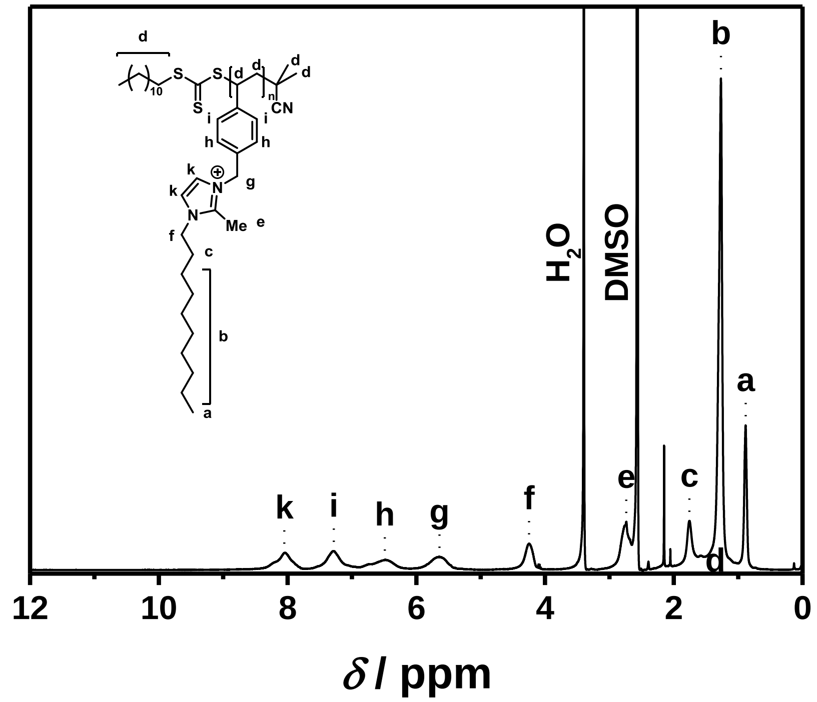


**Figure S14** $^{1}H$ NMR (400 MHz, 298 K) of p([DeMVBIM]Cl) (**5**) in DMSO-$d_6$.

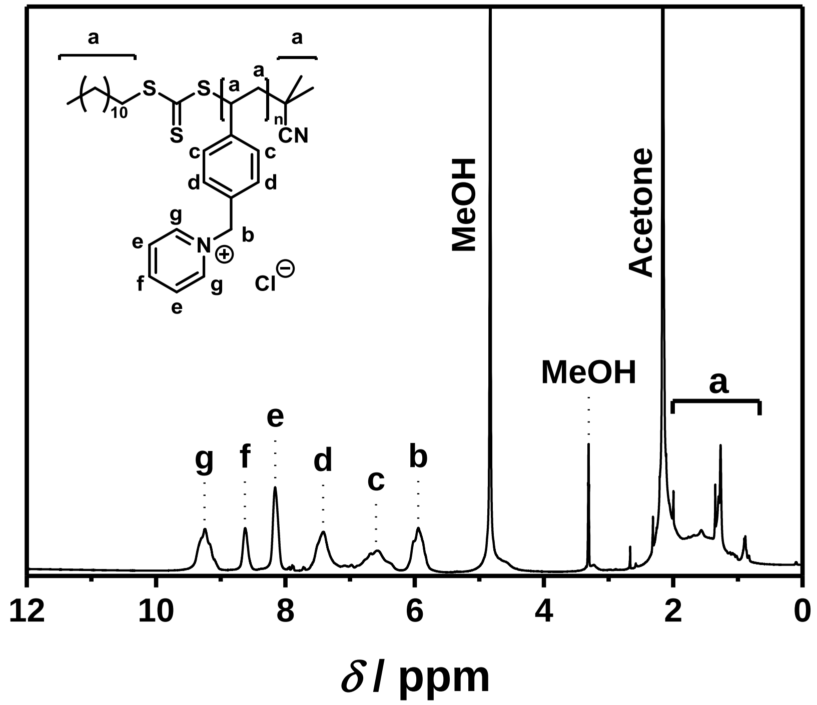


**Figure S15** $^{1}H$ NMR (400 MHz, 298 K) of p([VBPy]Cl) (**6**) in MeOD.

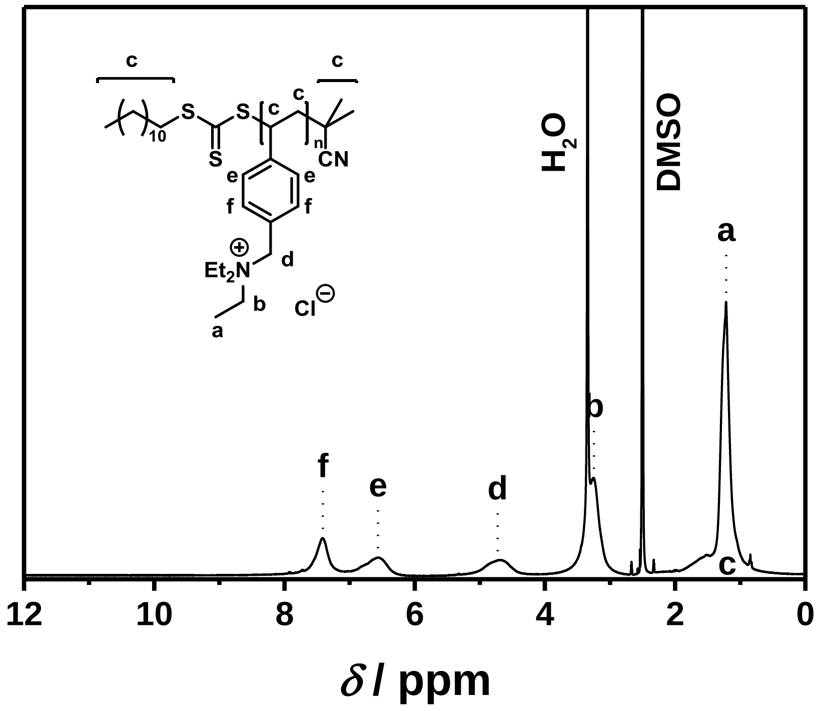


**Figure S16** $^{1}H$ NMR (400 MHz, 298 K) of p([TEVBA]Cl) (**7**) in DMSO-$d_6$.

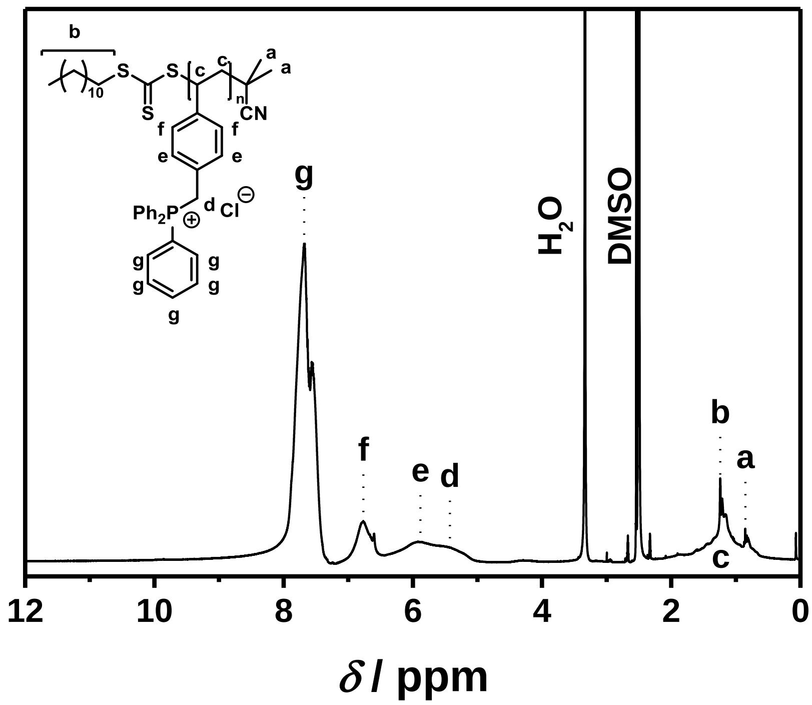


**Figure S17** $^{1}H$ NMR (400 MHz, 298 K) of p([TPVBP]Cl) (**8**) in DMSO-$d_6$.

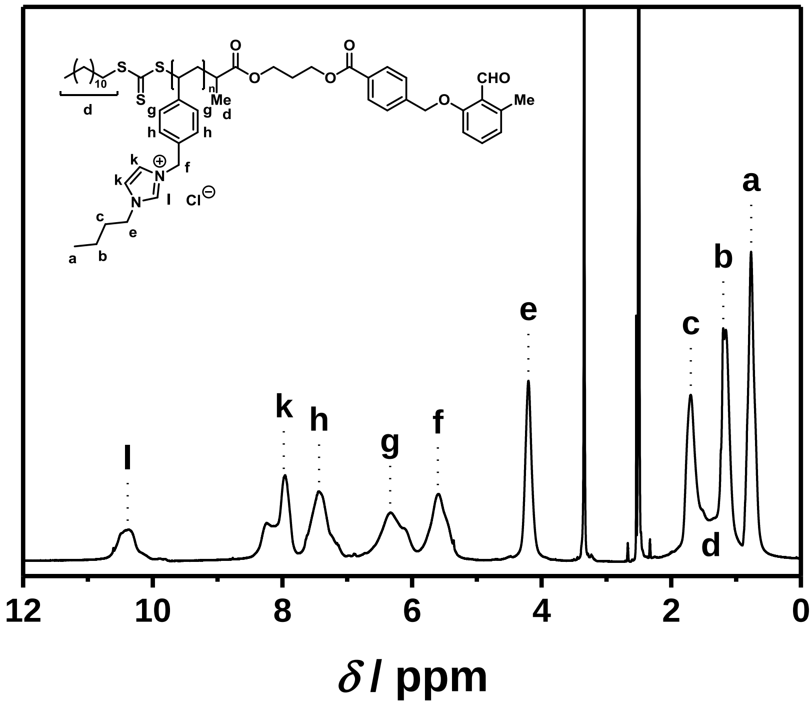


**Figure S18** $^{1}H$ NMR (400 MHz, 298 K) of p([BVBIM]Cl) (**11**) in DMSO-$d_6$.

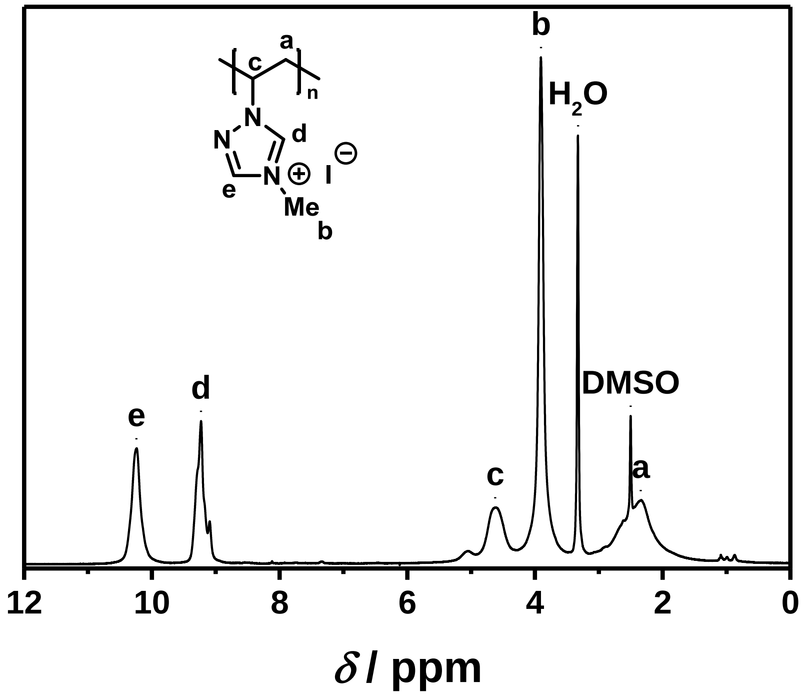


**Figure S19** $^{1}$H NMR (400 MHz, 298 K) of p([MVTr]I) (**13**) in DMSO-$d_6$.

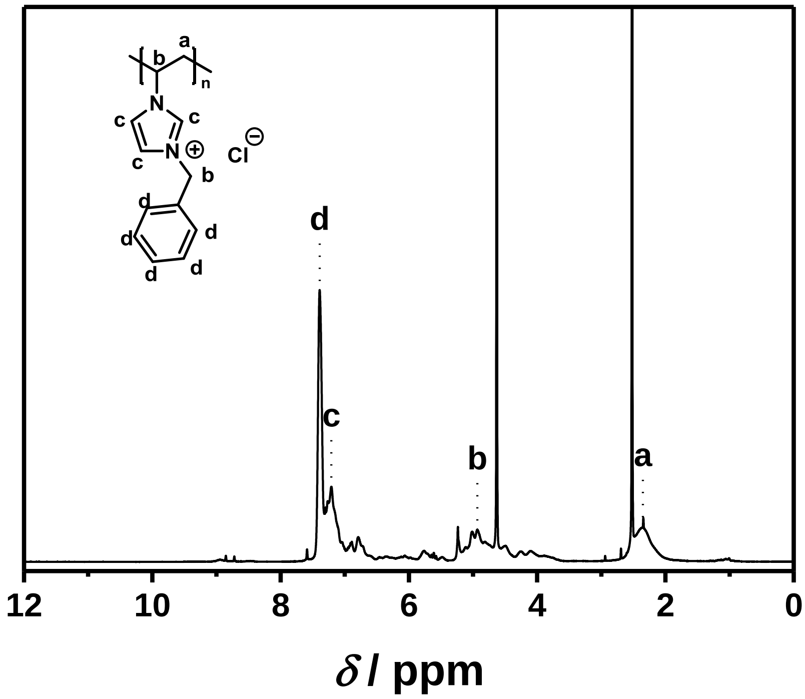


**Figure S20** $^{1}$H NMR (400 MHz, 298 K) of p([BnVIM]Cl) (**15**) in $D_2O$.

## SEC data

A detailed kinetic analysis of the polymerization of the employed ILs and a careful screening of the SEC conditions for the PILs were reported in a recent publication of our team.[1]

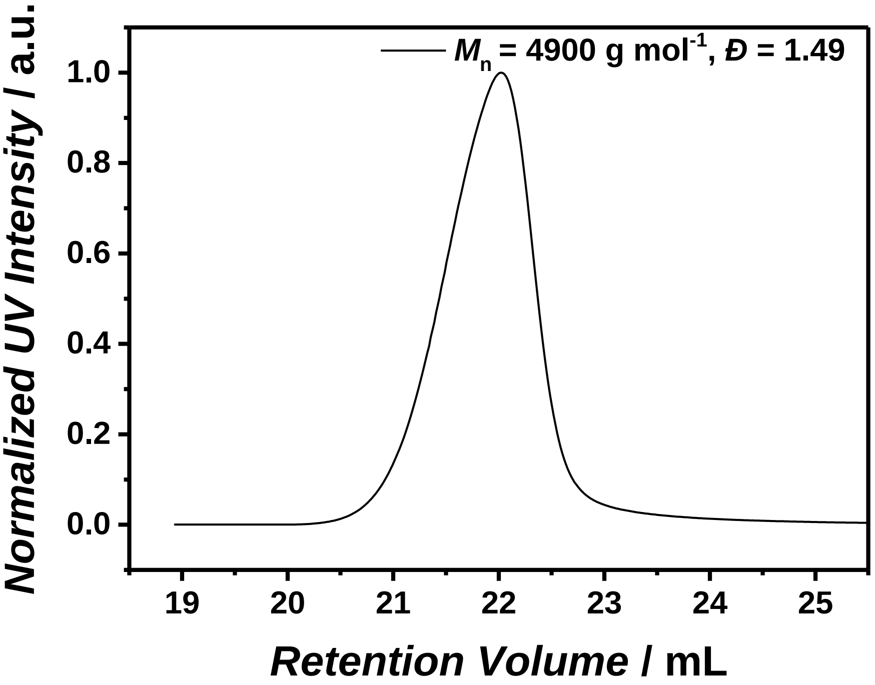


**Figure S21** SEC trace (eluent: water/0.3 M formic acid/0.5 g·L$^{-1}$ NaCl) of p([DeMVBIM]Cl) (**5**).

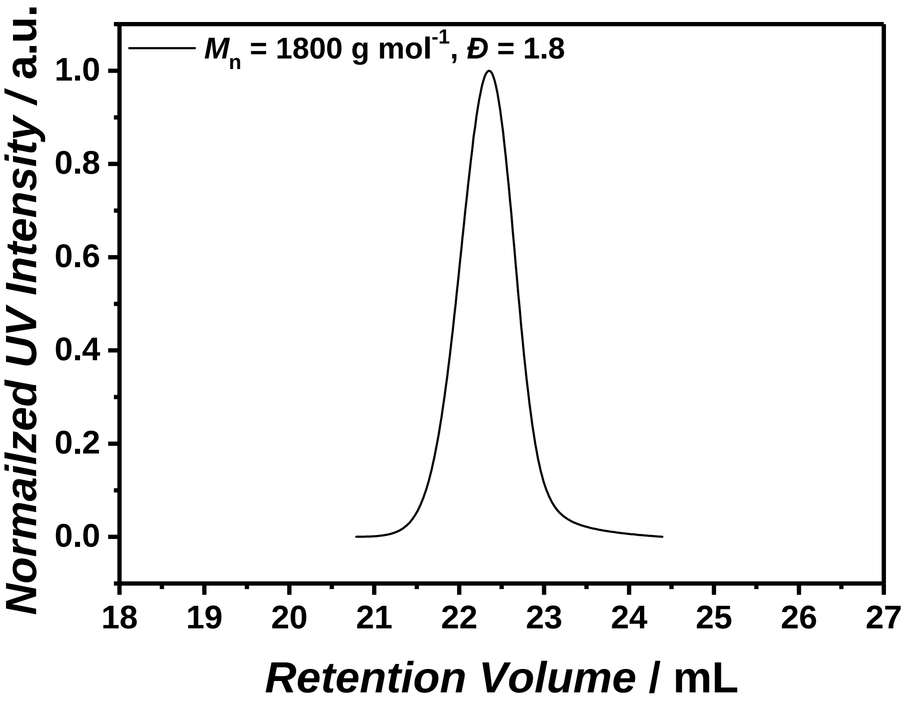


**Figure S22** SEC trace (eluent: water/0.3 M formic acid/0.5 g·L$^{-1}$ NaCl) of p([VBPy]Cl) (**6**).

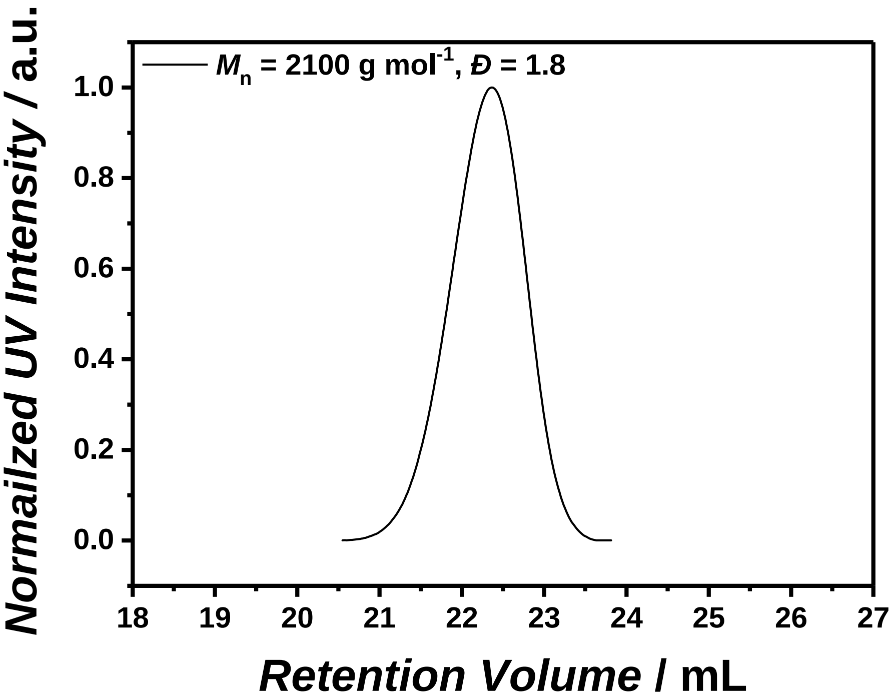


**Figure S23** SEC trace (eluent: water/0.3 M formic acid/0.5 g·L$^{-1}$ NaCl) of p([TEVBA]Cl) (**7**).

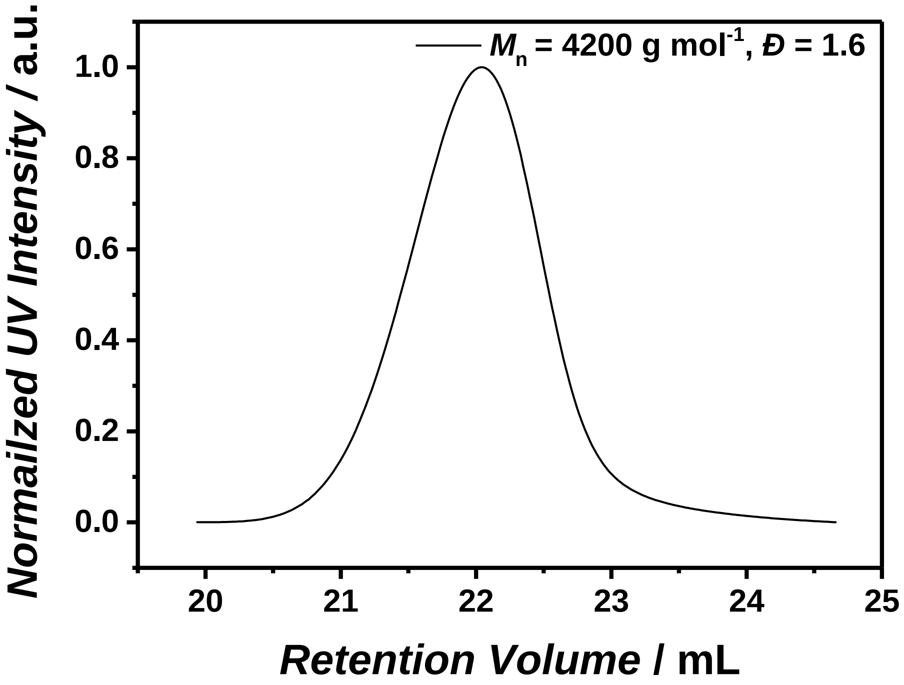


**Figure S24** SEC trace (eluent: water/0.3 M formic acid/0.5 g·L$^{-1}$ NaCl) of p([TPVBP]Cl) (**8**).

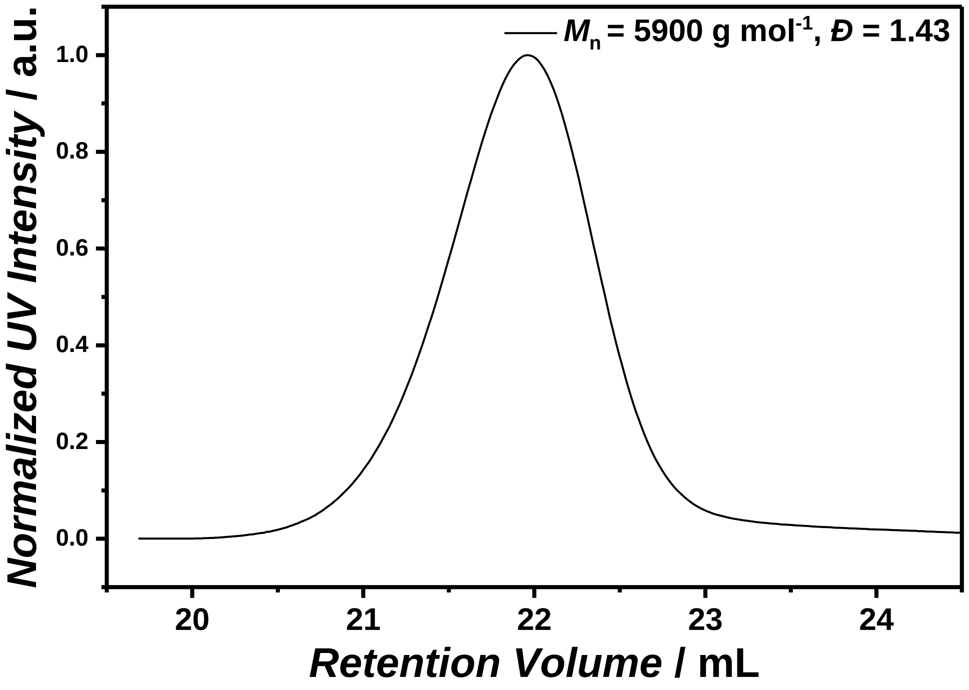


**Figure S25** SEC trace (eluent: water/0.3 M formic acid/0.5 g·L$^{-1}$ NaCl) of DoPAT-PE polymerized p([BVBIM]Cl) (**11**).

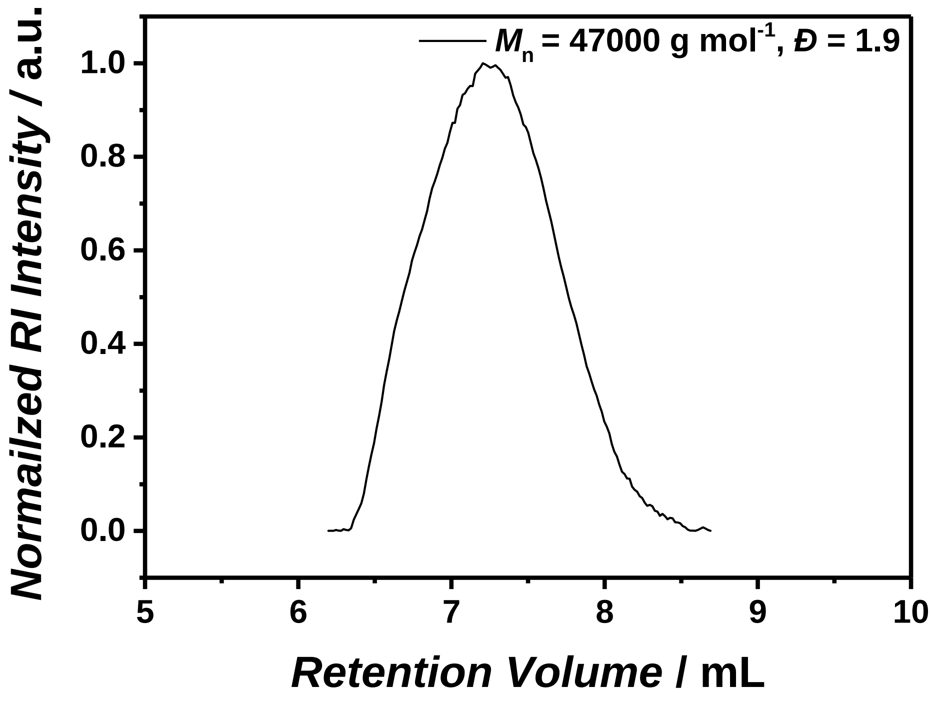


**Figure S26** SEC trace (eluent: aqueous acetate buffer/methanol (8:2, *v*/*v*), calibration standard pullulan) of p[([MVTr]I) (**13**). Please note that a preferential solvation of the PIL in a water/methanol mixture and the pullulan as calibration standard might lead to $M_n$ and $M_w$ values with a higher error.

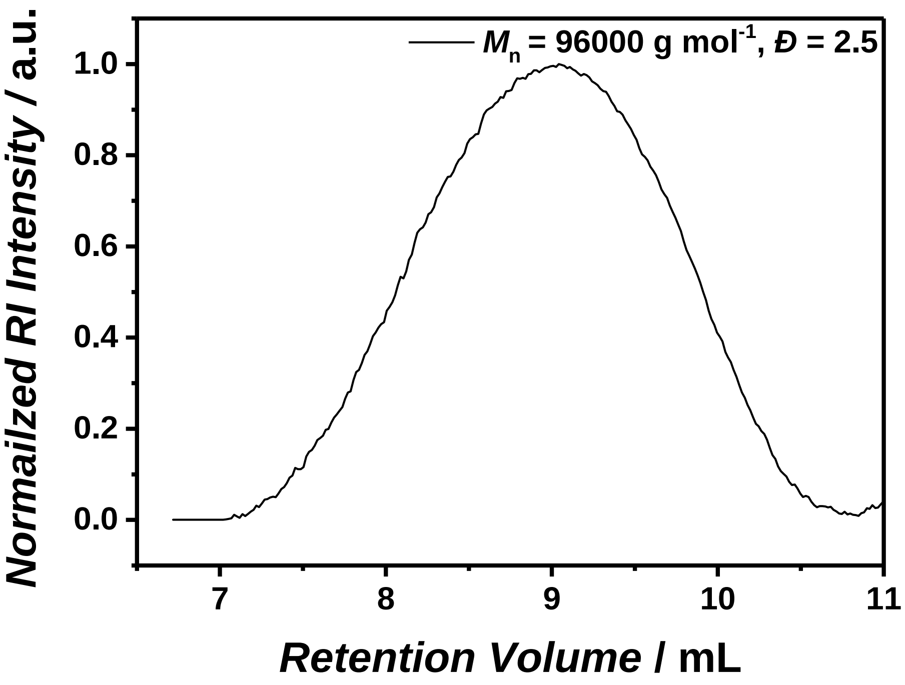


**Figure S27** SEC trace (eluent: aqueous acetate buffer/methanol (8:2, *v*/*v*), calibration standard pullulan) of p[([BnVIM]Cl) (**15**). Please note that a preferential solvation of the PIL in a water/methanol mixture and the pullulan as calibration standard may lead to $M_n$ and $M_w$ values with a higher error.

## MS Data

### Miscellaneous

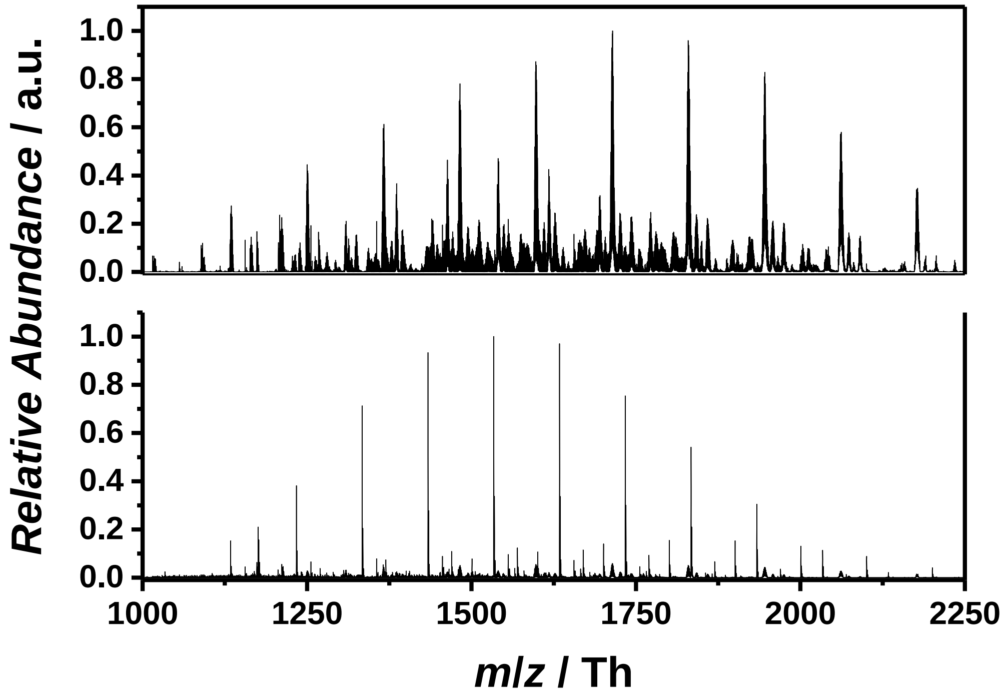


**Figure S28** (Top) ESI-CID-Orbitrap spectrum of p([VBPy]Cl) (**6**) employing 10 eV as collision energy. (Bottom) ESI-Orbitrap spectrum of p([VBPy]Cl) (**6**) without employing additional collision energy. Based on information of Thermo Scientific, the background signals stem from residual and hard to remove Ultramark® calibration markers with a characteristic repeating unit of 99.99 Th. These are always visible in negative ion mode.

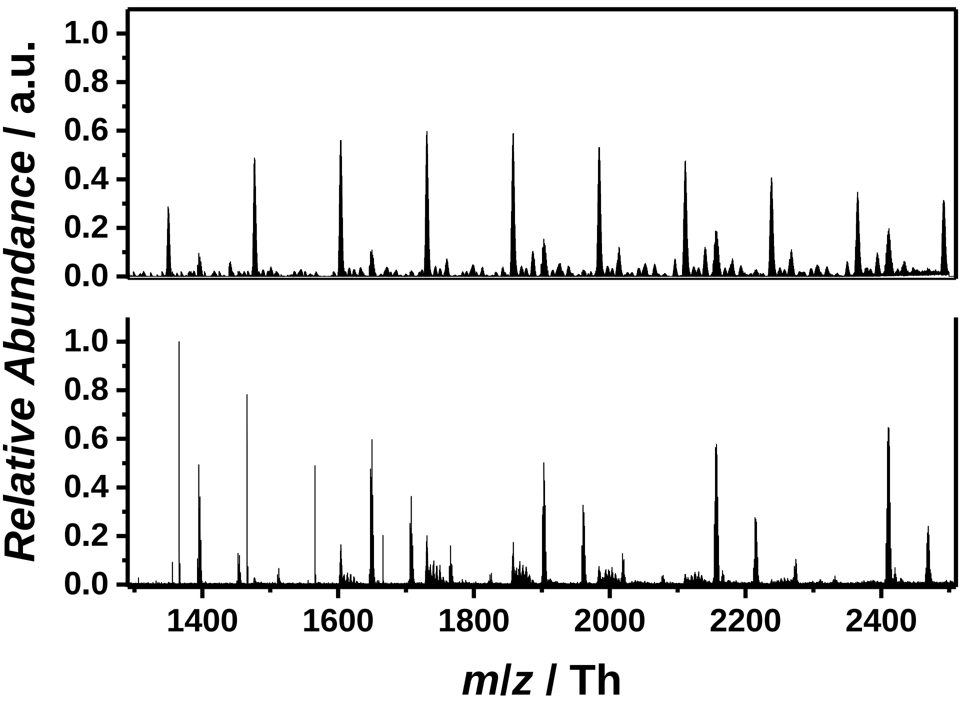


**Figure S29** (Top) ESI-CID-Orbitrap spectrum of p([TEVBA]Cl) (**7**) utilizing 1% (*v*/*v*) propylene carbonate (PC) as supercharging agent. (Bottom) ESI-CID-Orbitrap spectrum of p([TEVBA]Cl) (**7**) without any auxiliary additive to the ESI solvent (water/acetonitrile 50:50).

## Mass spectra of p([DeMVBIM]Cl) (5)

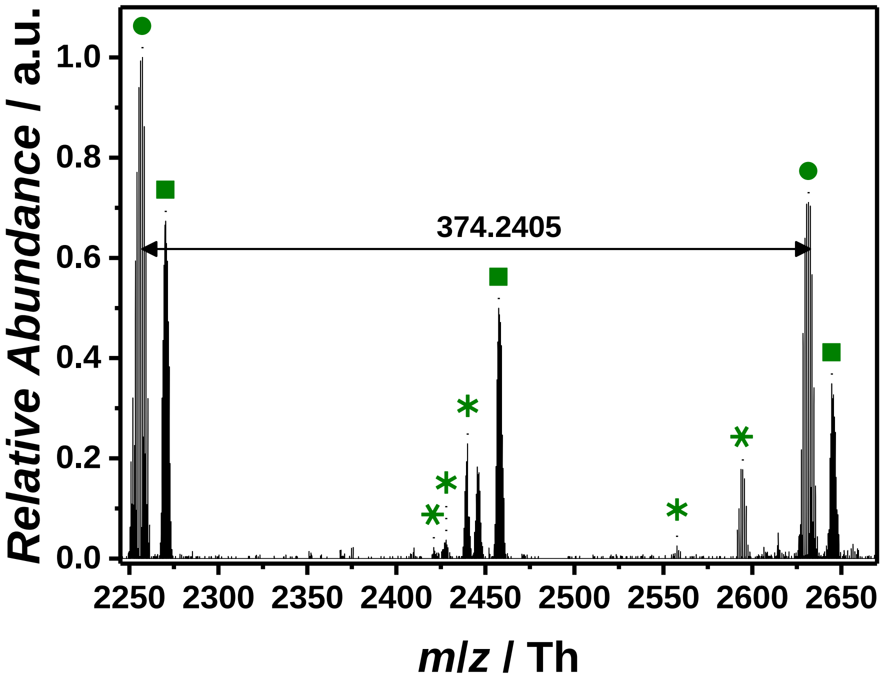


**Figure S30** Zoomed spectrum (negative mode) of p([DeMVBIM]Cl) (**5**) obtained *via* ESI-CID-Orbitrap MS doped with 2.0% (*v*/*v*) propylene carbonate depicting the repeating unit of *m*/*z* = 374.2405 Th (*m*/z(theo) = 347.2489 Th) of the most abundant species (labelled with ●). Species labelled with ✱ derive from (multiple) loss(es) of gaseous HCl.

**Table S1** Peak assignment of the ESI-CID-Orbitrap spectrum of p([DeMVBIM])Cl (**5**) from *m*/*z* = 2690 Th to *m*/*z* = 2870 Th showing the label (in correspondence to the species in **Figure S30**), the experimental *m*/*z* and theoretical *m*/*z* values (determined by the most abundant isotope of the isotopic pattern), Δ*m*/*z*, the resolution (obtained by the Xcalibur software), the number of repeating units *n*, and the structure determination. Due to the deprotonation process, no structure was determined for species labelled with ✱.

| Label | ***m*/*z*** **(exp) [Th]** | ***m*/*z*** **(theo) [Th]** | **Δ*m*/*z*** **[Th]** | **Resolution** | ***n*** | **Structure** |
|---|---|---|---|---|---|---|
| ● | 2256.3679 | 2256.3758 | 0.0078 | 43600 | 5 | 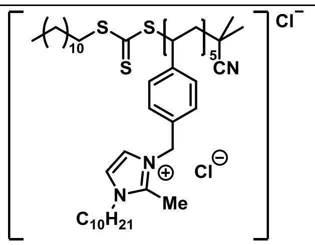 |
| ■ | 2270.4142 | 2270.4196 | 0.0053 | 44200 | 11 | 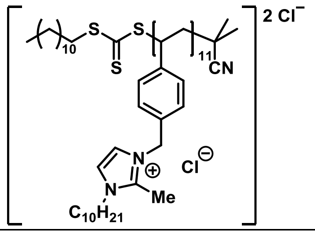 |

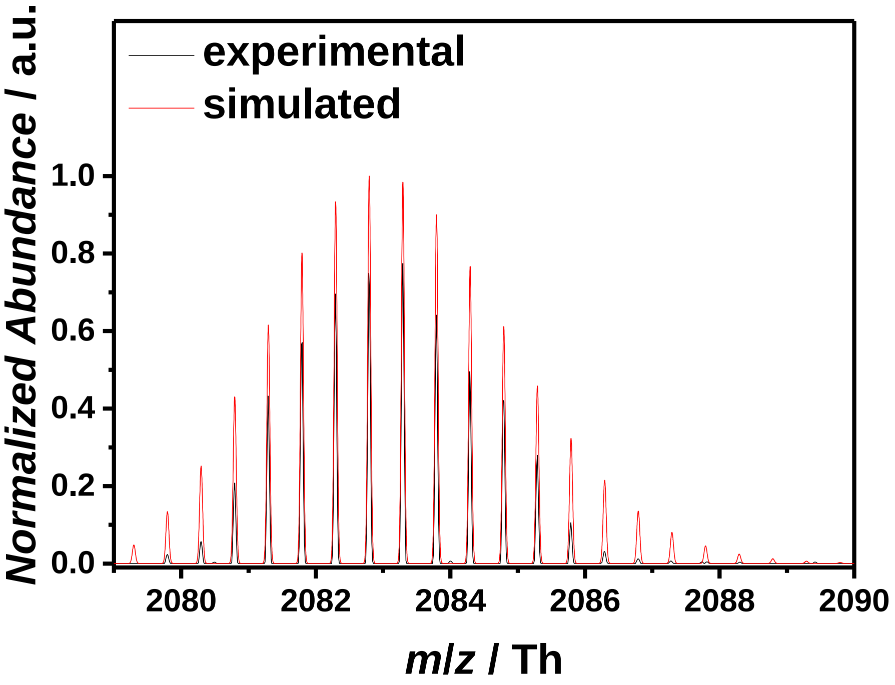


**Figure S31** Isotopic pattern of one selected peak at 2083 Th comparing the experiment (black line) and the simulation (red line) with a resolution of 45900.

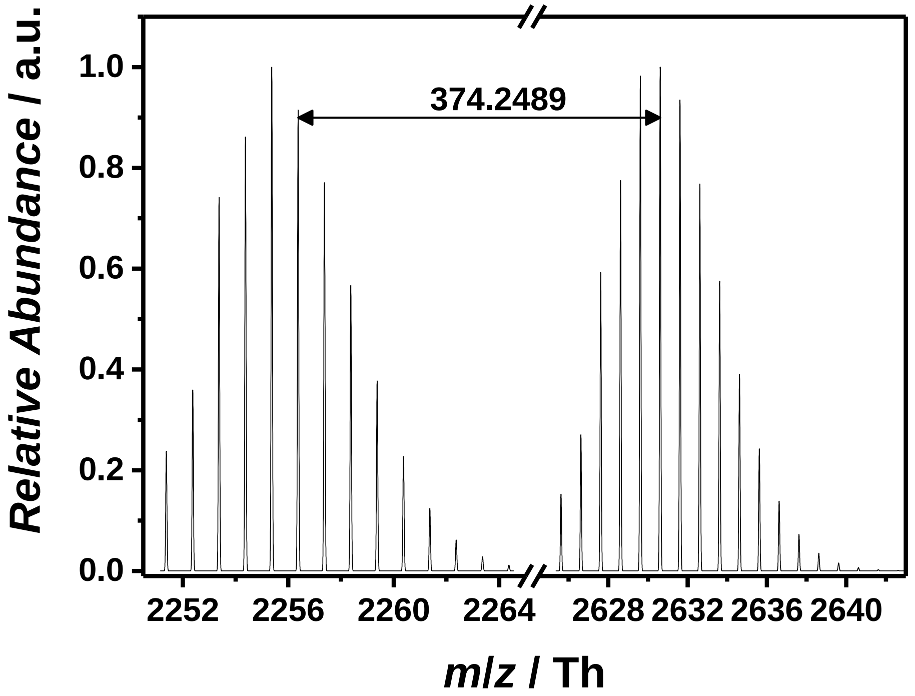


**Figure S32** Illustration of two simulated isotopic pattern representing the species at 2256 Th and 2631 Th. The difference of the 91% intensity peak and the 100% intensity peak corresponds to the theoretical value of the repeating unit.

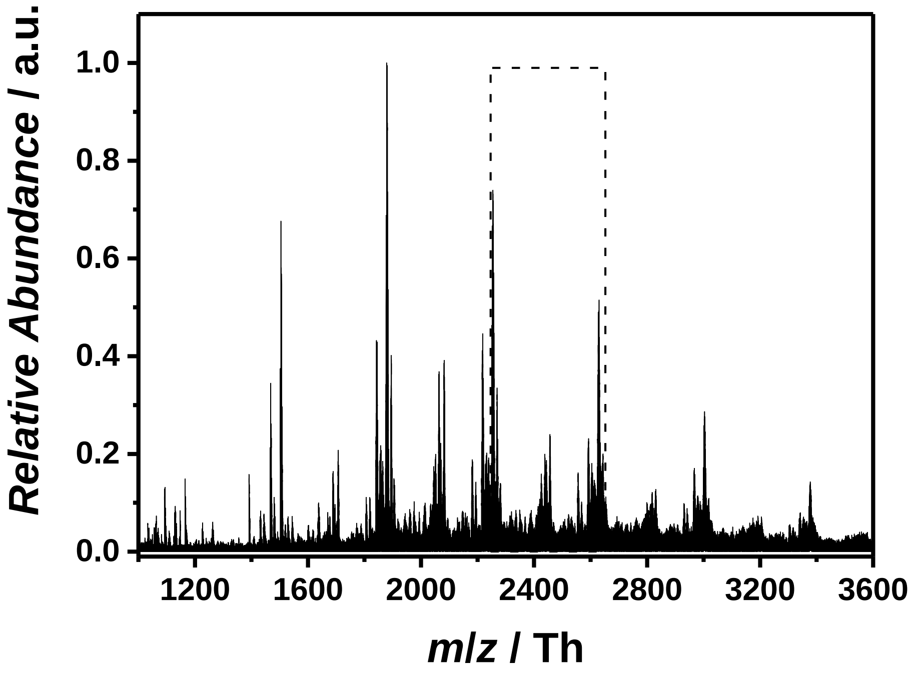


**Figure S33** Overview spectrum (negative mode) of p([DeMVBIM]Cl) (**5**) obtained *via* ESI-QToF MS utilizing $H_2O$/acetonitrile (1:1, *v*/*v*) as solvent.

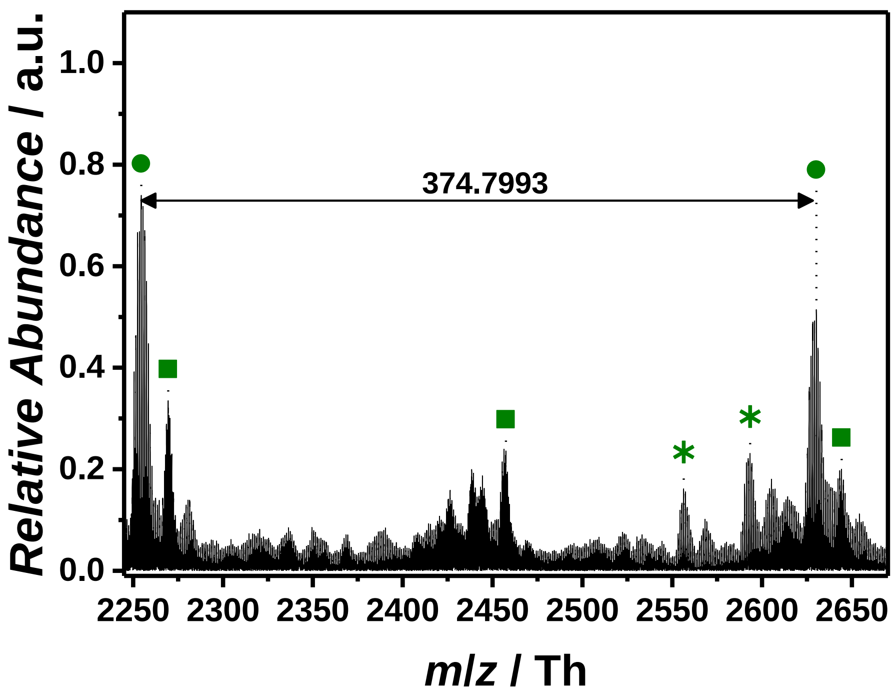


**Figure S34** Zoomed spectrum (negative mode) of p([DeMVBIM]Cl) (**5**) obtained *via* ESI-QToF MS depicting the repeating unit of *m*/*z* = 374.7993 Th (*m*/*z*(theo) = 347.2489 Th) of the most abundant species (labelled with ●). Species labelled with * derive from (multiple) loss(es) of gaseous HCl.

**Tandem MS experiment of p([DeMVBIM]Cl) (5)**

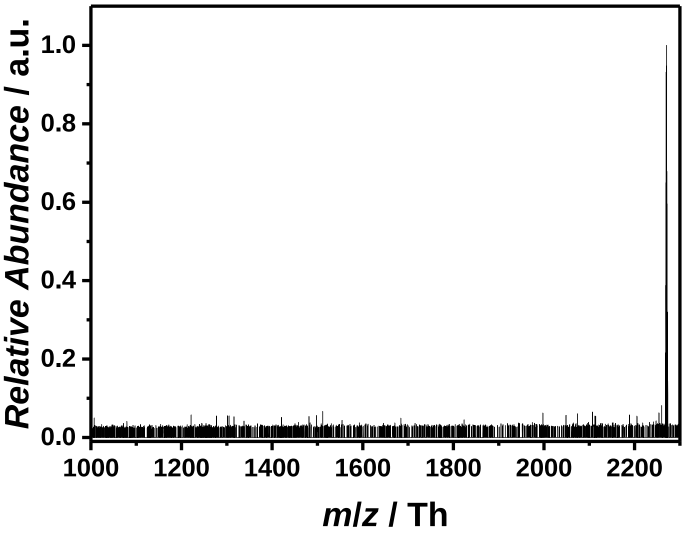


**Figure S35** Tandem MS experiment (negative mode) of a double charged species at 2270 Th under harsh conditions employing a higher-energy collision dissociation (HCD) of 40 eV and a collision induced dissociation (CID) energy of 25 eV. No apparent reverse Menshutkin fragmentation was observed although a feasible cleavage of the imidazolium moiety was expected due to a – for a nucleophilic attack favored – planar geometry of the imidazolium.

**Mass spectrum of p([VBPy]Cl) (6)**

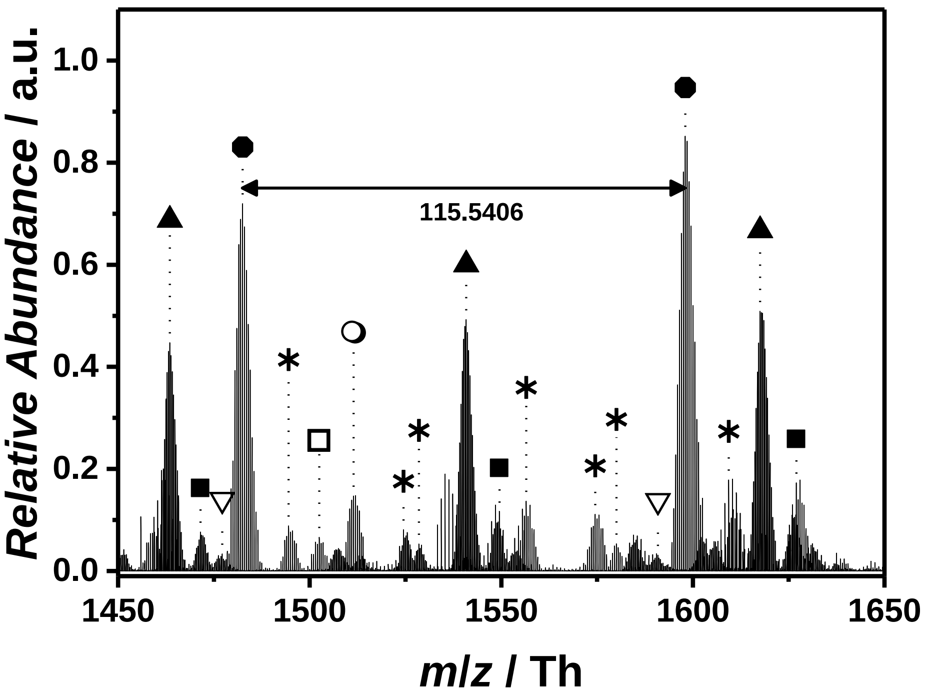


**Figure S36** Zoomed spectrum (negative mode) of p([VBPy])Cl (**6**) obtained *via* ESI-CID-Orbitrap MS depicting the repeating unit of *m/z* = 115.5406 Th (*m/z*(theo) = 115.5413 Th) of the most abundant species (labelled with ●). Species labelled with ✱ derive from (multiple) loss(es) of gaseous HCl.

**Table S2** Peak assignment of the ESI-CID-Orbitrap spectrum of p([VBPy])Cl (**6**) from *m/z* = 1460 Th to *m/z* = 1500 Th showing the label (in correspondence to the species in **Figure S36**), the experimental *m/z* and theoretical *m/z* values (determined by the most abundant isotope of the isotopic pattern), Δ*m/z*, the resolution (obtained by the Xcalibur software), the number of repeating units *n*, and the structure determination. Due to the deprotonation process, no structure was determined for species labelled with ✱.

| Label | *m/z* (exp) [Th] | *m/z* (theo) [Th] | Δ*m/z* [Th] | Resolution | *n* | Structure |
|---|---|---|---|---|---|---|
| ▲ | 1463.4843 | 1463.4834 | 0.0009 | 53500 | 17 | 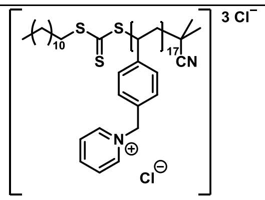  |
| ■ | 1471.4959 | 1471.5015 | 0.0056 | 49500 | 17 | 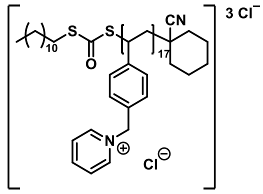  |

| | | | | | | |
|---|---|---|---|---|---|---|
| ▽ | 1476.8310 | 1476.8272 | 0.0039 | 54600 | 17 | 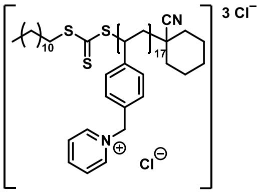 |
| ● | 1482.4991 | 1482.4967 | 0.0024 | 53800 | 11 | 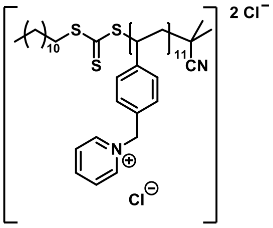 |
| □ | 1502.5176 | 1502.5124 | 0.0052 | 52600 | 11 | 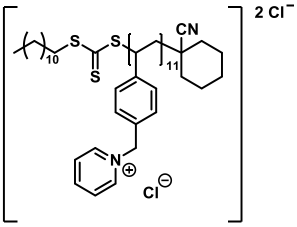 |
| ❍ | 1511.4758 | 1511.4758 | 0.0000 | 53300 | 11 | 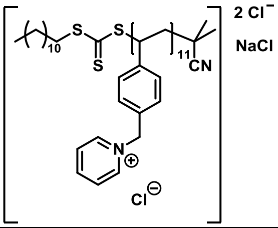 |

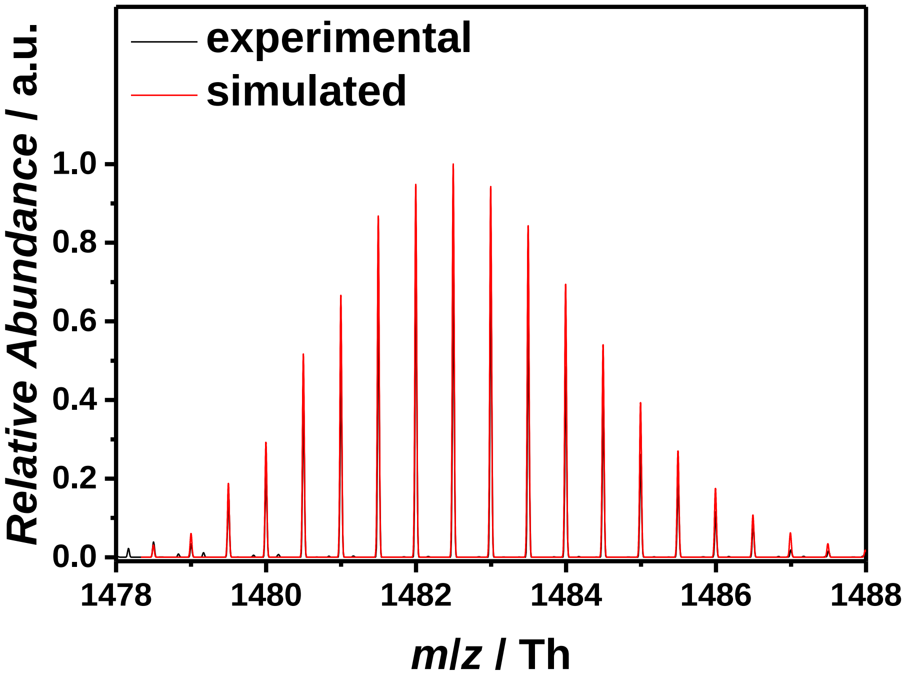


**Figure S37** Isotopic pattern of one selected peak at 1482 Th comparing the experiment (black line) and the simulation (red line) with a resolution of 53800.

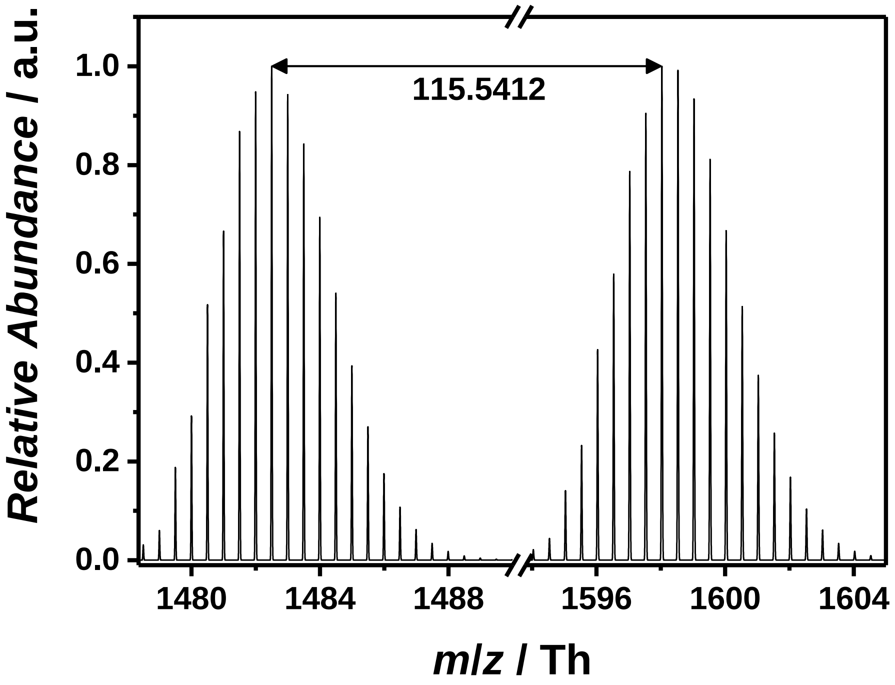


**Figure S38** Illustration of two simulated isotopic pattern representing the species at 1482 Th and 1598 Th. The difference of each highest peaks corresponds to the theoretical value of the repeating unit.

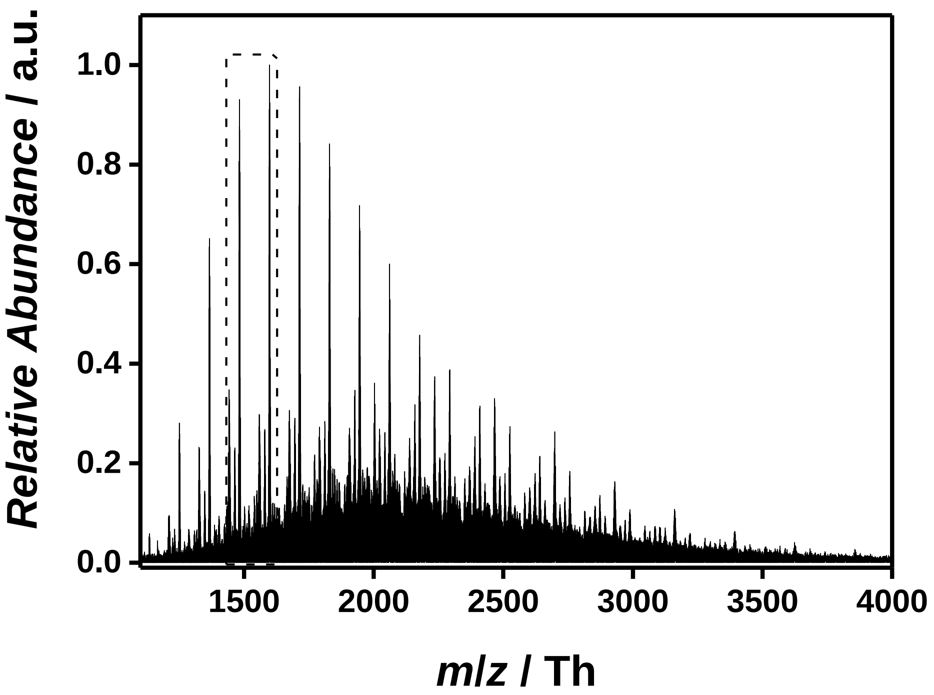


**Figure S39** Overview spectrum (negative mode) of p([VBPy])Cl (**6**) obtained *via* ESI-QToF MS utilizing $H_2O$/acetonitrile (1:1, *v*/*v*) as solvent.

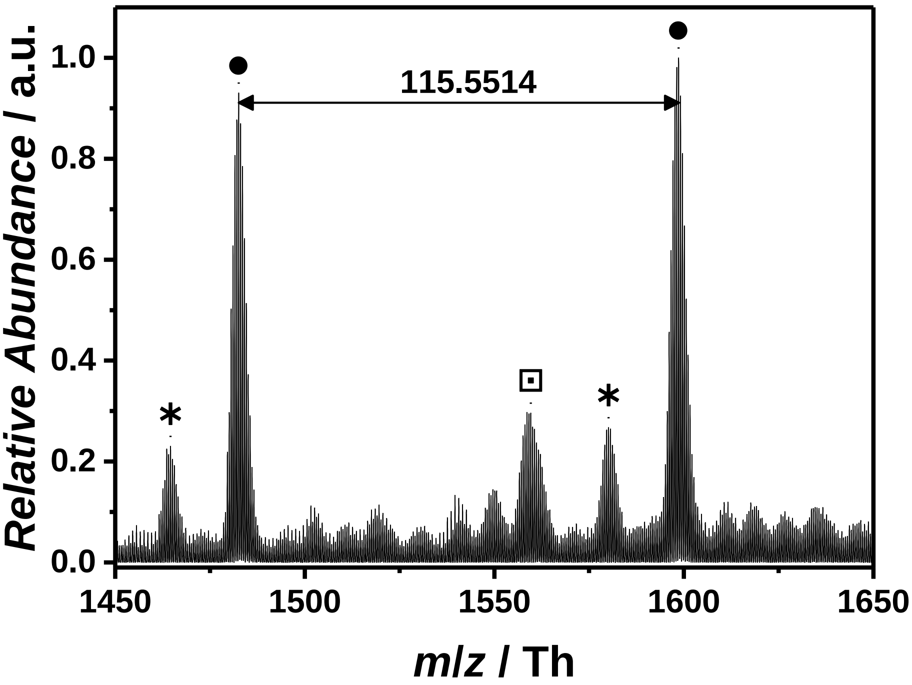


**Figure S40** Zoomed spectrum (negative mode) of p([VBPy])Cl (**6**) obtained *via* ESI-QToF MS depicting the repeating unit of *m/z* = 115.5514 Th (*m/z*(theo) = 115.5413 Th) of the most abundant species (labelled with ●) and the labelling of the most abundant species above 20%. Species labelled with ✱ derive from (multiple) loss(es) of gaseous HCl.

**Table S3** Peak assignment of the ESI-QToF spectrum of p([VBPy])Cl (**6**) of *m/z* = 1558 Th showing the label, the experimental *m/z* and theoretical *m/z* values (determined by the most abundant isotope of the isotopic pattern), Δ*m/z*, the resolution (obtained by the MassLynx software), the number of repeating units *n*, and the structure determination. The remaining labels are in correspondence to **Table S2**.

| Label | *m/z* (exp) [Th] | *m/z* (theo) [Th] | Δ*m/z* [Th] | Resolution | *n* | Structure |
|---|---|---|---|---|---|---|
| ⊡ | 1558.6033 | 1558.5162 | 0.0871 | 11000 | 12 | 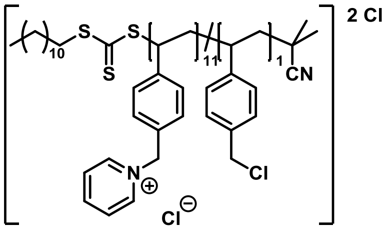 |

**Tandem MS experiment of p([VBPy]Cl) (5)**

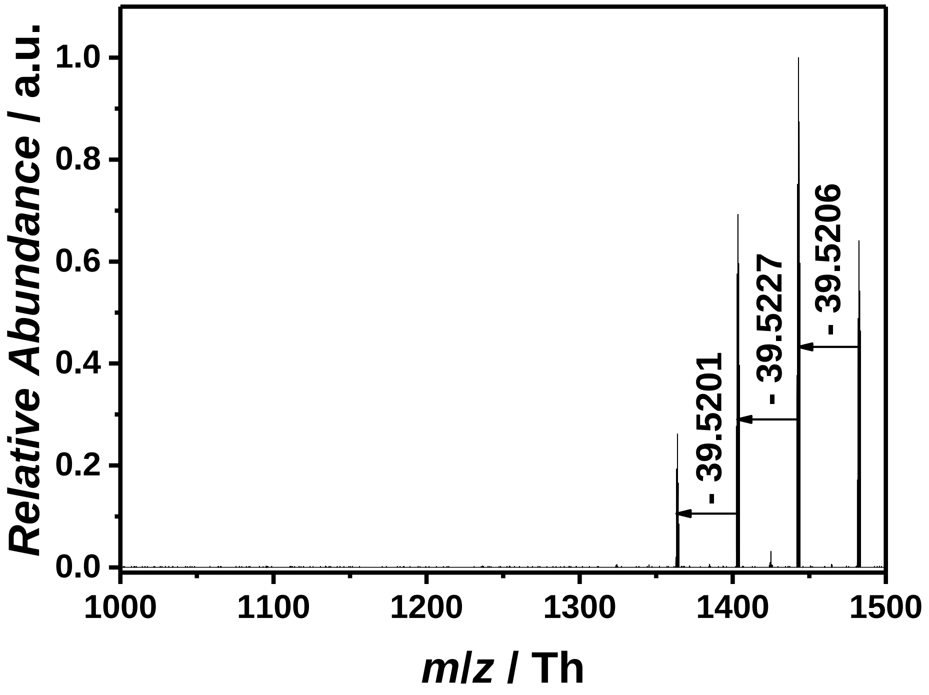


**Figure S41** Tandem MS experiment (negative mode) of a double charged species at 1482 Th with a higher-energy collision dissociation (HCD) of 10 eV. The identification of three double charged species depicting the loss of a pyridine unit (*m/z*(theo) = 39.5216 Th).

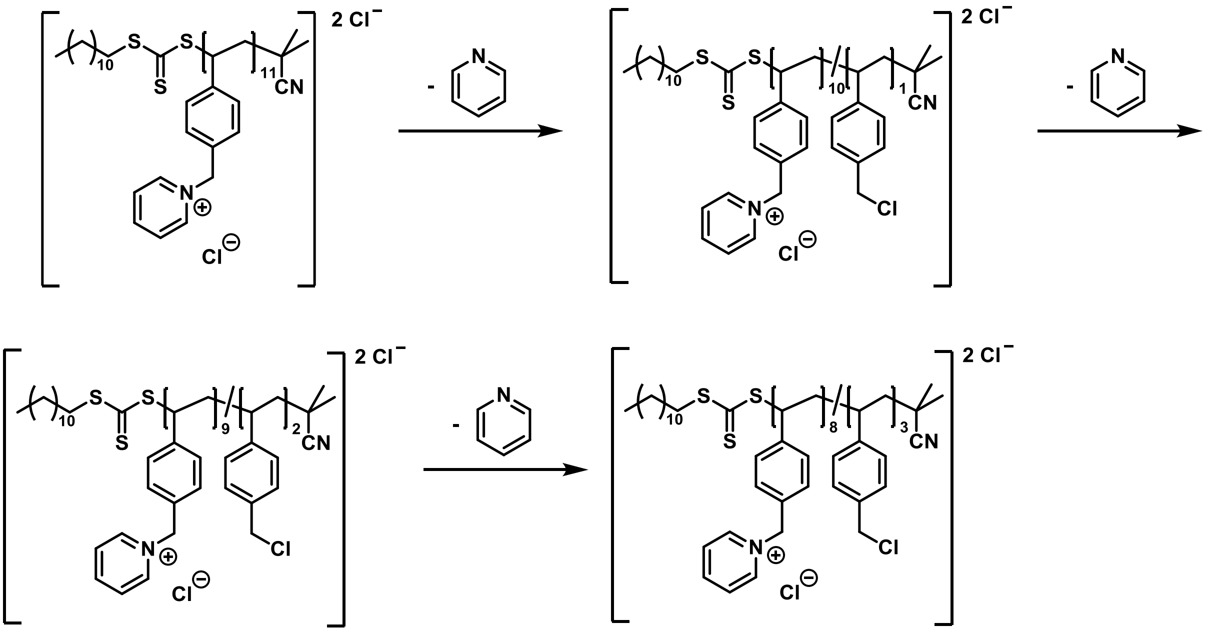


**Scheme S1** Proposed fragmentation of p([VBPy]Cl) (**6**) *via* a reverse Menshutkin mechanism including the stepwise nucleophilic attack of the chloride anion at the electrophilic benzylic moiety.

**Mass spectrum of p([TEVBA]Cl) (7)**

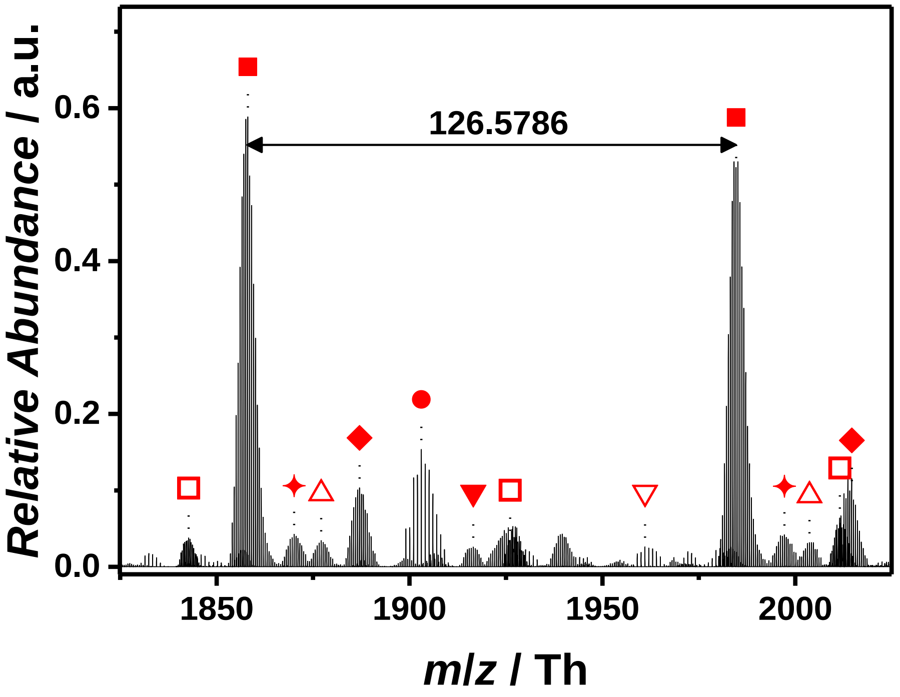


**Figure S42** Zoomed spectrum (negative mode) of p([TEVBA])Cl (**7**) obtained *via* ESI-CID-Orbitrap MS doped with 0.5% (*v*/*v*) propylene carbonate depicting the repeating unit of *m*/*z* = 126.5786 Th (*m/z(theo)* = 126.5799 Th) of the most abundant species (labelled with ■).

**Table S4** Peak assignment of the ESI-CID-Orbitrap spectrum of p([TEVBA])Cl (**7**) from *m*/*z* = 1840 Th to *m*/*z* = 1960 Th showing the label (in correspondence to the species in **Figure S42**), the experimental *m*/*z* and theoretical *m*/*z* values (determined by the most abundant isotope of the isotopic pattern), Δ*m*/*z*, the resolution (obtained by the Xcalibur software), the number of repeating units *n*, and the structure determination.

| Label | ***m*/*z*** **(exp) [Th]** | ***m*/*z*** **(theo) [Th]** | **Δ*m*/*z*** **[Th]** | **Resolution** | ***n*** | **Structure** |
|---|---|---|---|---|---|---|
| □ | 1842.7522 | 1842.7530 | 0.0008 | 50300 | 20 | 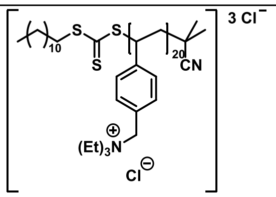 |
| ■ | 1858.0859 | 1858.0867 | 0.0008 | 49900 | 13 | 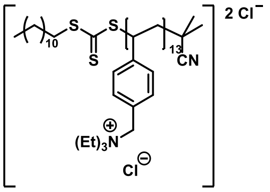 |

| | | | | | | |
|---|---|---|---|---|---|---|
| ✦ | 1870.5965 | 1870.5950 | 0.0015 | 46000 | 13 | 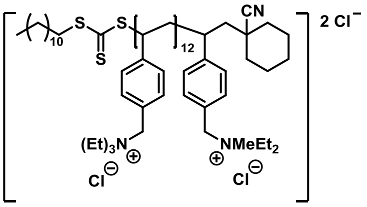 |
| △ | 1877.1090 | 1877.1031 | 0.0059 | 48500 | 13 | 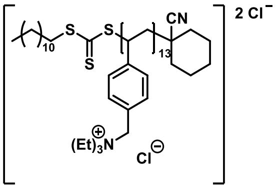 |
| ◆ | 1887.0626 | 1887.0659 | 0.0033 | 48000 | 13 | 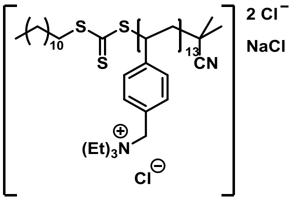 |
| ● | 1903.0852 | 1903.0837 | 0.0014 | 48127 | 6 | 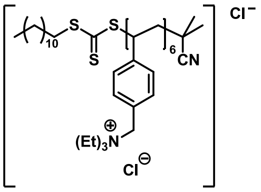 |
| ▼ | 1916.5422 | 1916.5447 | 0.0025 | 48500 | 13 | 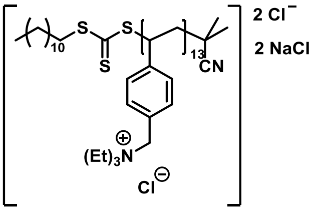 |
| ▽ | 1961.0461 | 1961.0461 | 0.0000 | 45100 | 6 | 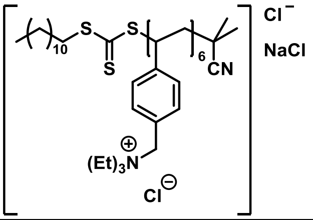 |

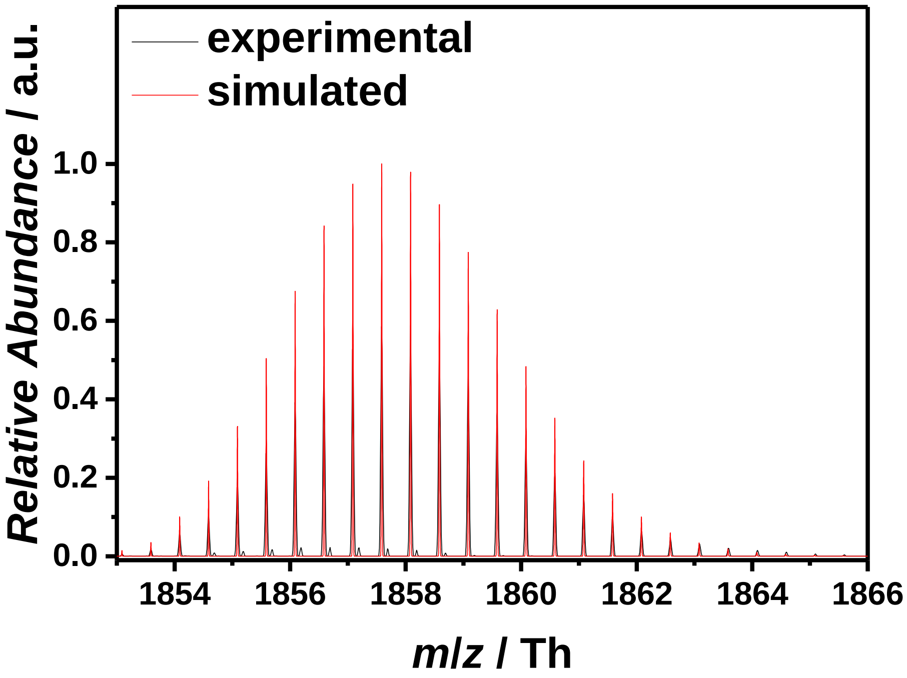


**Figure S43** Isotopic pattern of one peak at 1858 Th comparing the experiment (black line) and the simulation (red line) with a resolution of 49900.

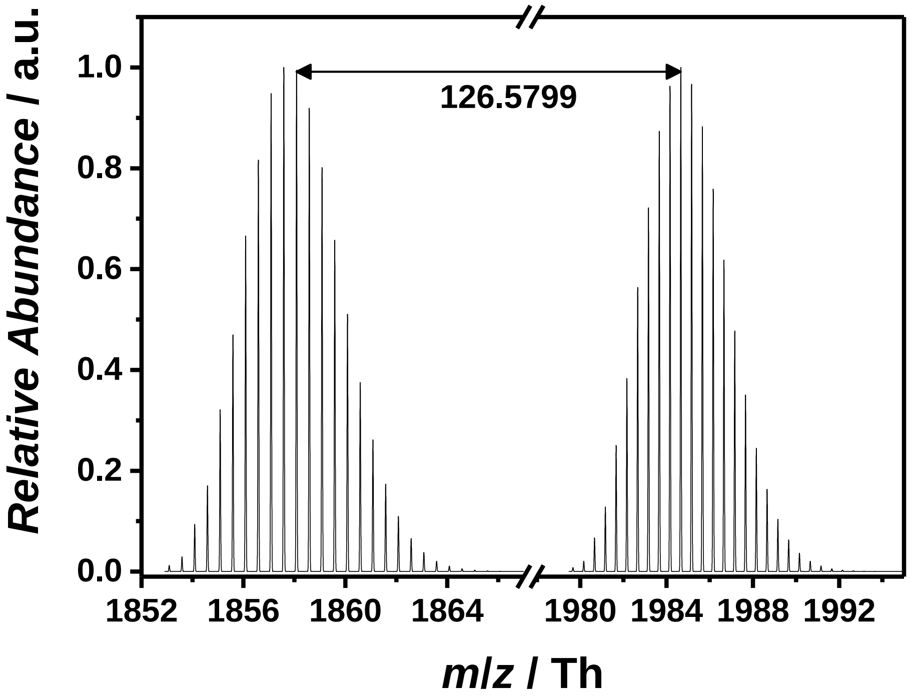


**Figure S44** Illustration of two simulated isotopic pattern representing the species at 1858 Th and 1984 Th. The difference of the 98% intensity peak and the 100% intensity peak corresponds to the theoretical value of the repeating unit.

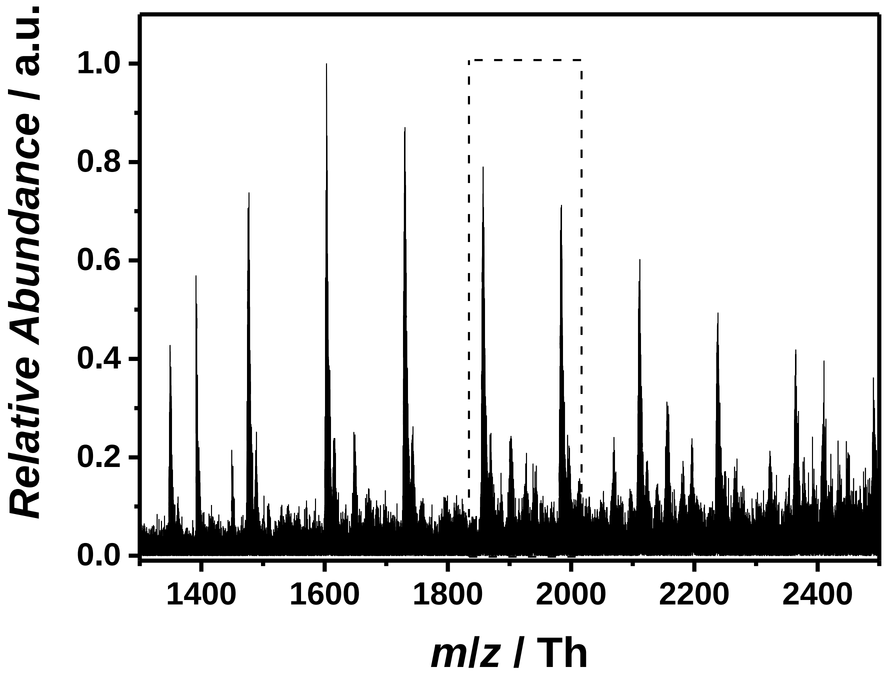


**Figure S45** Overview spectrum (negative mode) of p([TEVBA])Cl (**7**) obtained *via* ESI-QToF MS utilizing $H_2O$/acetonitrile (1:1, *v*/*v*) as solvent.

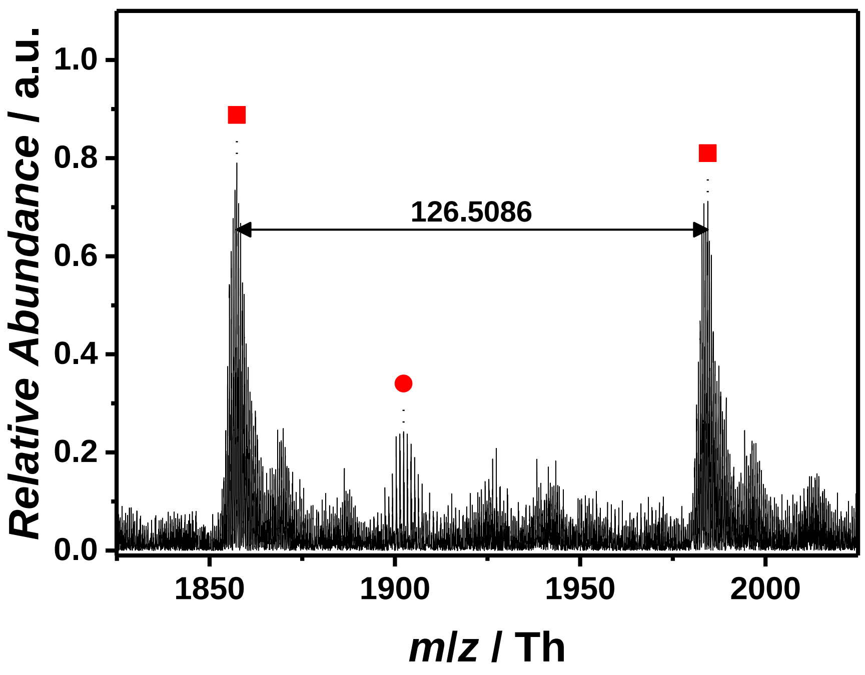


**Figure S46** Zoomed spectrum (negative mode) of p([TEVBA])Cl (**7**) obtained *via* ESI-QToF MS depicting the repeating unit of *m*/*z* = 126.5086 Th (*m*/*z*(theo) = 126.5799 Th) of the most abundant species (labelled with ■).

**Tandem MS experiment of p([TEVBA])Cl (7)**

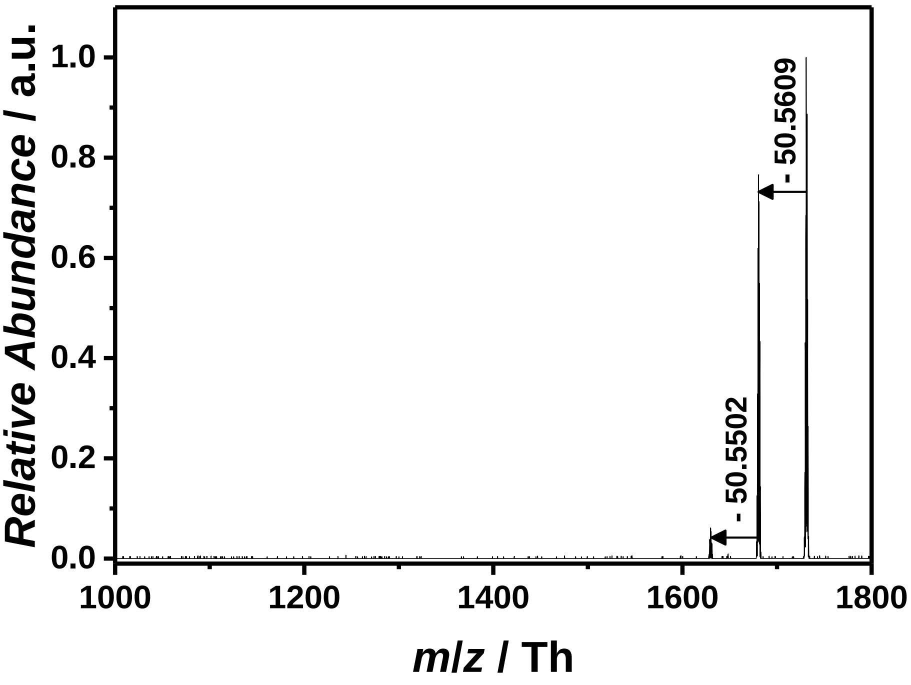


**Figure S47** Tandem MS experiment (negative mode) of a double charged species at 1731 Th with a higher-energy collision dissociation (HCD) of 35 eV. The identification of two double charged species depicting the loss of a triethylamine unit (*m/z*(theo) = 50.5608 Th).

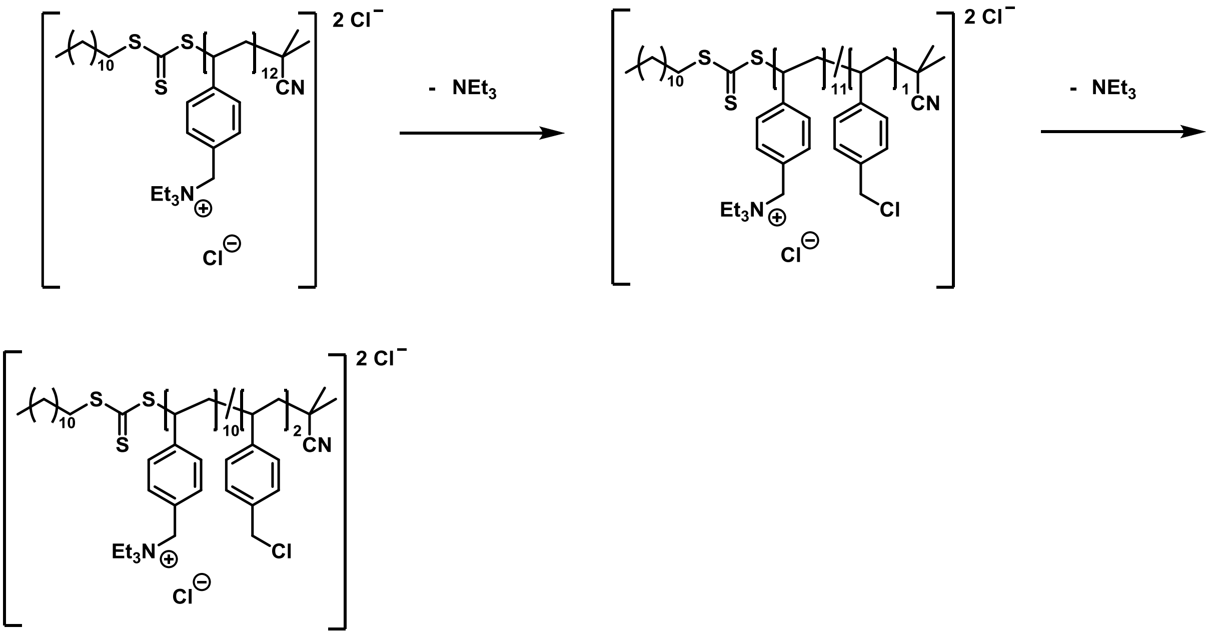


**Scheme S2** Proposed fragmentation process of p([TEVBA])Cl (**7**) *via* a reverse Menshutkin mechanism including the stepwise nucleophilic attack of the chloride anion at the electrophilic benzylic moiety.

**Mass spectra of p([TPVBP]Cl) (8)**

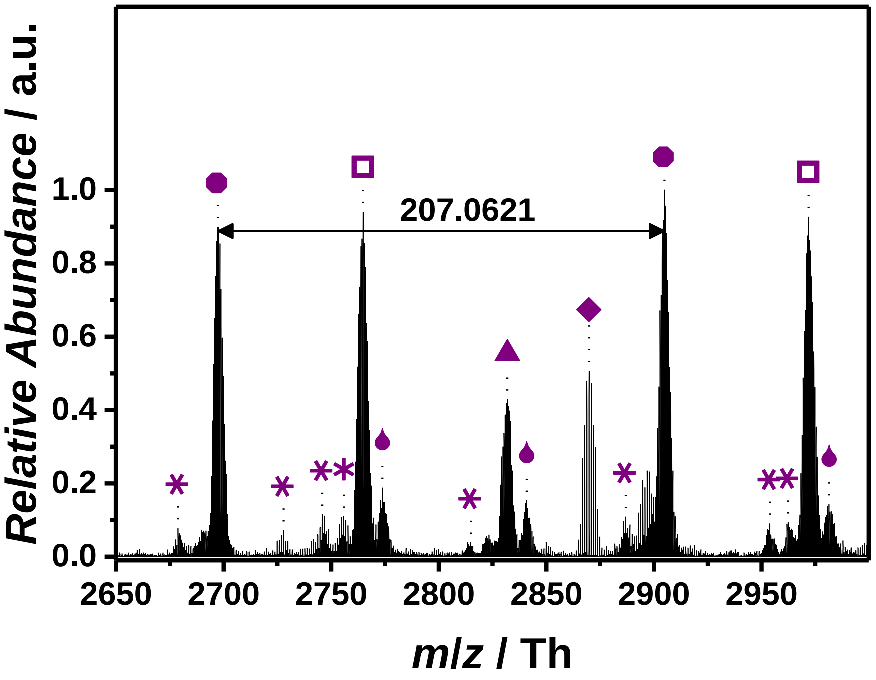


**Figure S48** Zoomed spectrum (negative mode) of p([TPVBP]Cl) (**8**) obtained *via* ESI-CID-Orbitrap MS doped with 1.0% (*v*/*v*) propylene carbonate depicting the repeating unit of *m/z* = 207.0621 Th (*m/z(*theo) = 207.0658 Th) of the most abundant species (labelled with ●). Species labelled with ✳ derive from (multiple) loss(es) of gaseous HCl.

**Table S5** Peak assignment of the ESI-CID-Orbitrap spectrum of p([TPVBP])Cl (**8**) from *m/z* = 2690 Th to *m/z* = 2870 Th showing the label (in correspondence to the species in **Figure S48**), the experimental *m/z* and theoretical *m/z* values (determined by the most abundant isotope of the isotopic pattern), Δ*m/z*, the resolution (obtained by the Xcalibur software), the number of repeating units *n*, and the structure determination. Due to the deprotonation process, no structure was determined for species labelled with ✳. Species labelled with 💧 are $H_2O$ adducts.

| Label | *m/z* (exp) [Th] | *m/z* (theo) [Th] | Δ*m/z* [Th] | Resolution | *N* | Structure |
|---|---|---|---|---|---|---|
| ● | 2697.3183 | 2697.3339 | 0.0156 | 40000 | 12 | 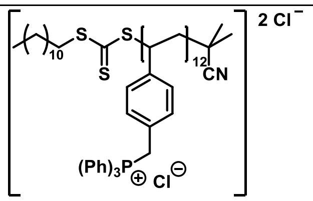 |
| □ | 2764.8487 | 2764.8705 | 0.0218 | 38600 | 13 | 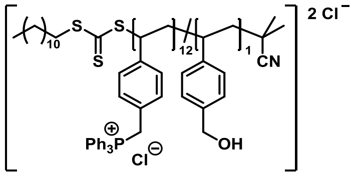 |

| | | | | | | |
|---|---|---|---|---|---|---|
| ▲ | 2831.8759 | 2831.9073 | 0.0314 | 35000 | 14 | 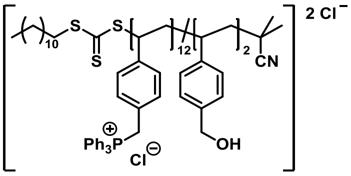 |
| ◆ | 2869.8960 | 2869.9152 | 0.0192 | 39000 | 6 | 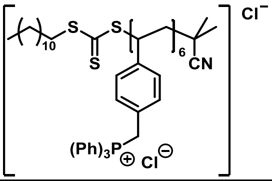 |

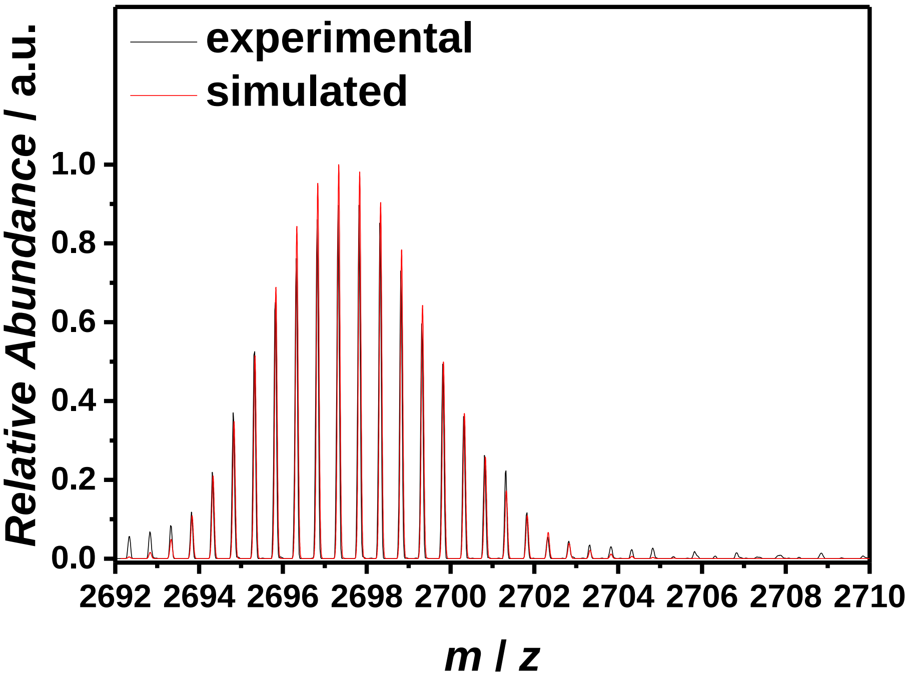


**Figure S49** Isotopic pattern of one selected peak at 2697 Th comparing the experiment (black line) and the simulation (red line) with a resolution of 40000.

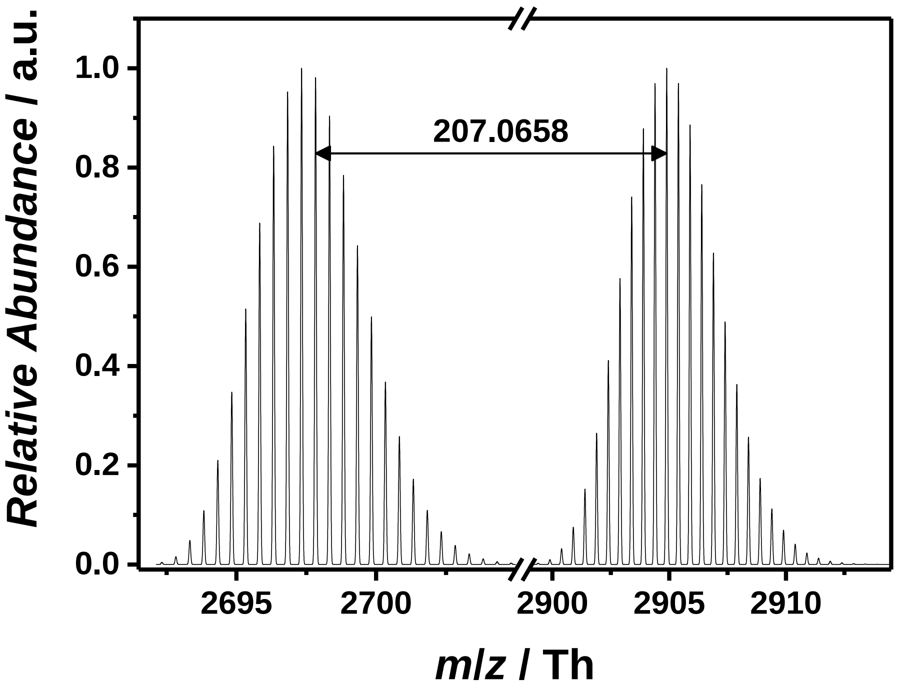


**Figure S50** Illustration of two simulated isotopic pattern representing the species at 2697 Th and 2904 Th. The difference of the 98% intensity peak and the 100% intensity peak corresponds to the theoretical value of the repeating unit.

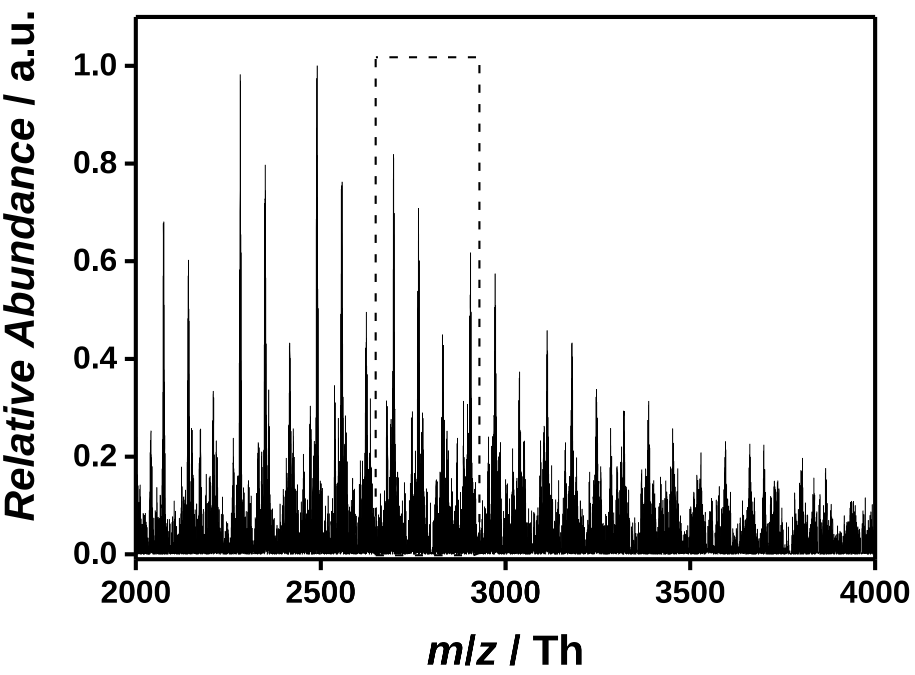


**Figure S51** Overview spectrum (negative mode) of p([TPVBP]Cl) (**8**) obtained *via* ESI-QToF MS utilizing $H_2O$/acetonitrile (1:1, *v*/*v*) as solvent.

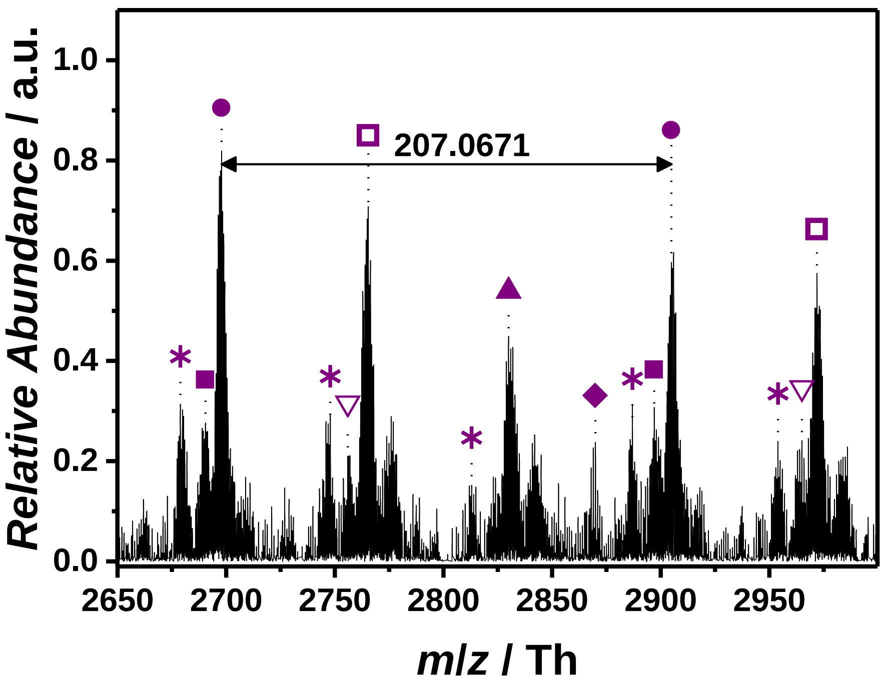


**Figure S52** Zoomed spectrum (negative mode) of p([TPVBP]Cl) (**8**) obtained *via* ESI-QToF MS depicting the repeating unit of $m/z$ = 207.0671 Th ($m/z$(theo) = 207.0658 Th) of the most abundant species (labelled with ●). Species labelled with ✳ derive from (multiple) loss(es) of gaseous HCl.

**Table S6** Peak assignment of the ESI-QToF spectrum of p([TPVBP]Cl) (**8**) from $m/z$ = 2697 Th to $m/z$ = 2697 Th showing the label, the experimental $m/z$ and theoretical $m/z$ values (determined by the most abundant isotope of the isotopic pattern), Δ$m/z$, the resolution (obtained by the MassLynx software), the number of repeating units $n$, and the structure determination. The remaining labels analogous to **Table S5**.

| Label | ***m/z*** **(exp) [Th]** | ***m/z*** **(theo) [Th]** | **Δ*m/z* [Th]** | **Resolution** | ***N*** | **Structure** |
|---|---|---|---|---|---|---|
| ■ | 2690.4163 | 2690.3763 | 0.0400 | 10804 | 14 | 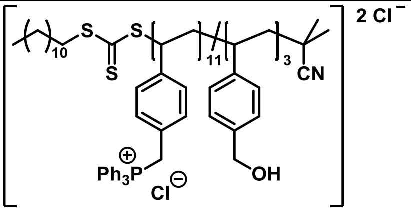 |
| ▽ | 2756.9531 | 2756.9160 | 0.0371 | 11862 | 15 | 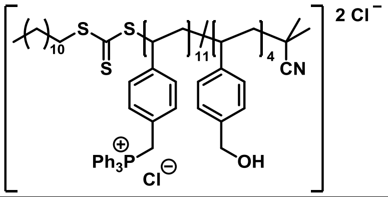 |

**Tandem MS experiment of p([TPVBP]Cl) (8)**

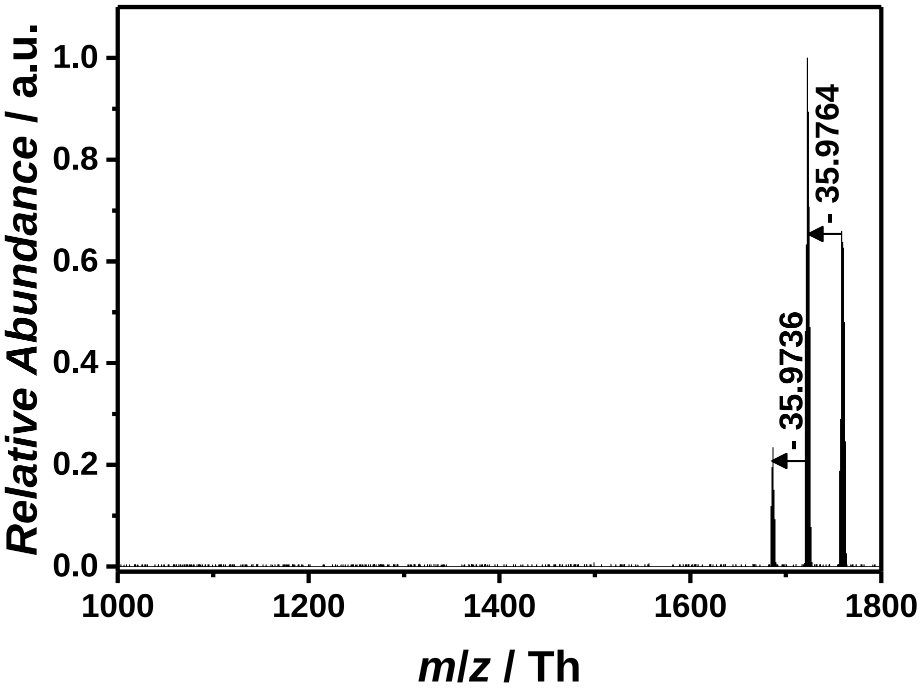


**Figure S53** Tandem MS experiment (negative mode) of a single charged species at 1758 Th with a higher-energy collision dissociation (HCD) of 24 eV. The identification of two single charged species depicting the loss of a gaseous HCl (*m/z*(theo) = 35.9767 Th).

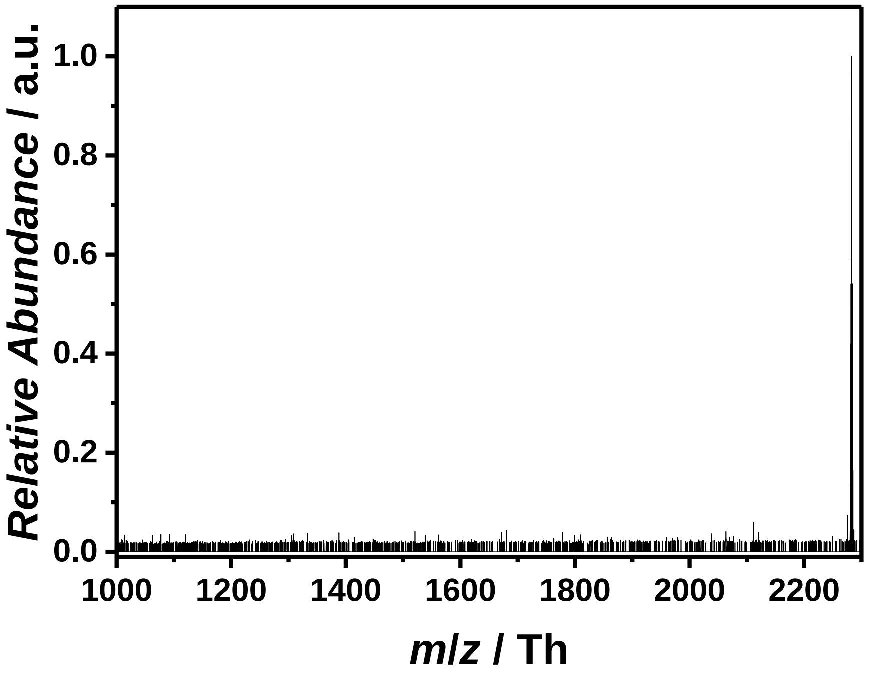


**Figure S54** Tandem MS experiment (negative mode) of a double charged species at 2282 Th under harsh conditions employing a higher-energy collision dissociation (HCD) of 10 eV and a collision induced dissociation (CID) energy of 40 eV. No apparent reverse Menshutkin fragmentation was observed postulating the steric hindrance of the $PPh_3$ core sheltering the vulnerable electrophilic benzylic moiety from being attacked by the chloride.

**Mass spectra of p([ATMEA]Cl) (10)**

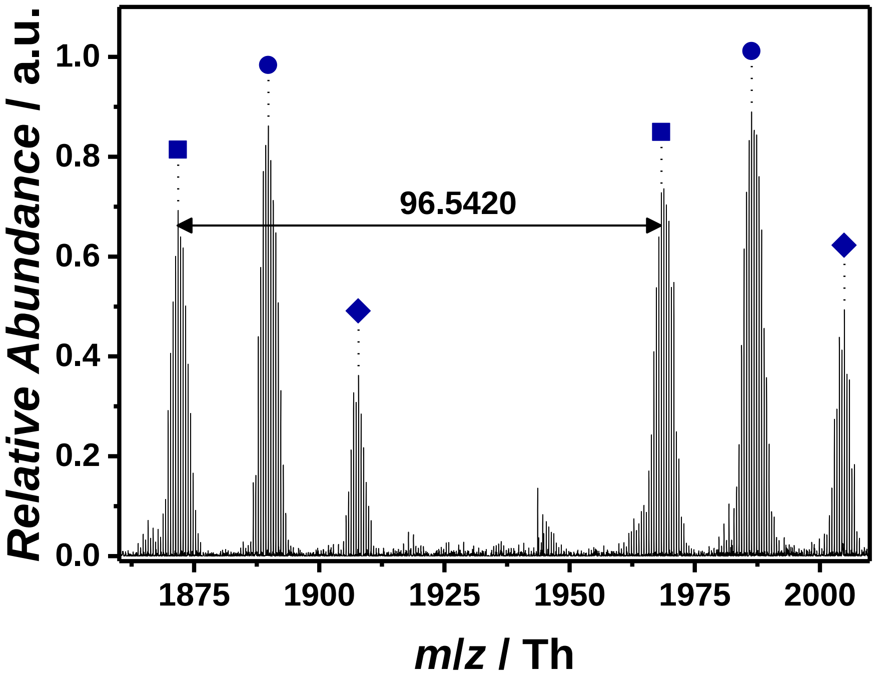


**Figure S55** Zoomed spectrum (negative mode) of p([ATMEA]Cl) (**10**) obtained *via* ESI-CID-Orbitrap MS doped with 1.0% (*v*/*v*) propylene carbonate depicting the repeating unit of *m*/*z* = 96.5420 Th (*m*/*z*(theo) = 96.5440 Th) of one species (labelled with ■).

**Table S7** Peak assignment of the ESI-CID-Orbitrap spectrum of p([ATMEA]Cl) (**10**) from *m*/*z* = 1775 Th to *m*/*z* = 1810 Th showing the label (in correspondence to the species in **Figure S55**), the experimental *m*/*z* and theoretical *m*/*z* values (determined by the most abundant isotope of the isotopic pattern), Δ*m*/*z*, the resolution (obtained by the Xcalibur software), the number of repeating units *n*, and the structure determination.

| Label | ***m*/*z*** **(exp) [Th]** | ***m*/*z*** **(theo) [Th]** | **Δ*m*/*z*** **[Th]** | **Resolution** | ***n*** | **Structure** |
|---|---|---|---|---|---|---|
| ■ | 1871.8086 | 1871.8082 | 0.0004 | 50800 | 18 | 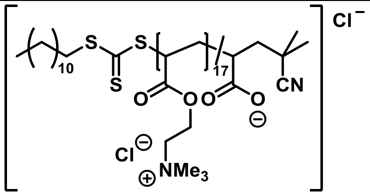 |
| ● | 1889.8308 | 1889.8307 | 0.0001 | 49900 | 19 | 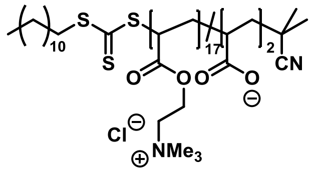 |
| ◆ | 1908.3517 | 1908.3530 | 0.0013 | 47300 | 20 | 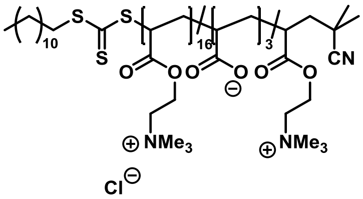 |

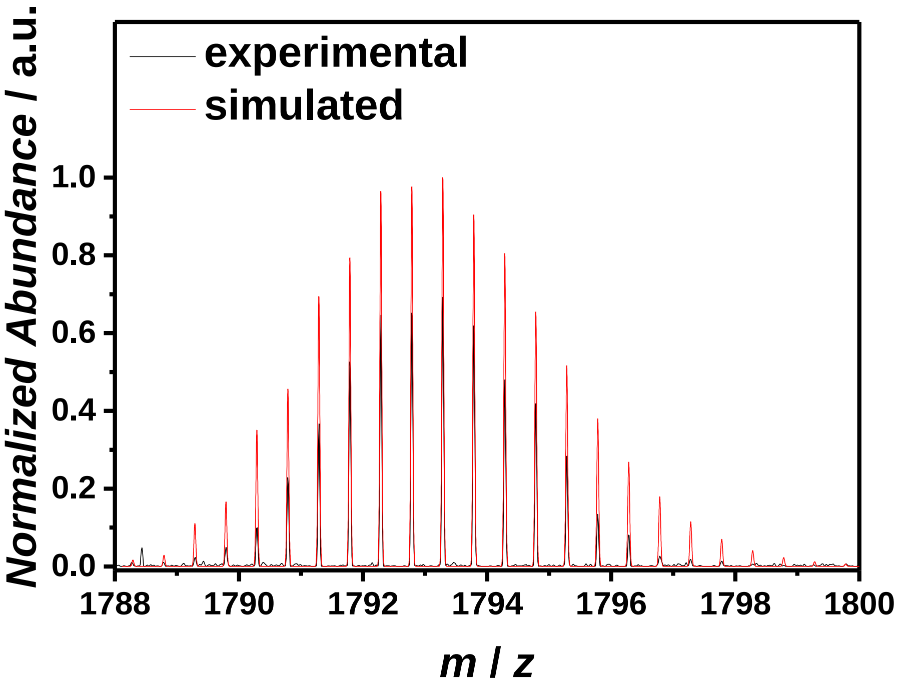


**Figure S56** Isotopic pattern of one selected peak at 1793 Th comparing the experiment (black line) and the simulation (red line) with a resolution of 50000.

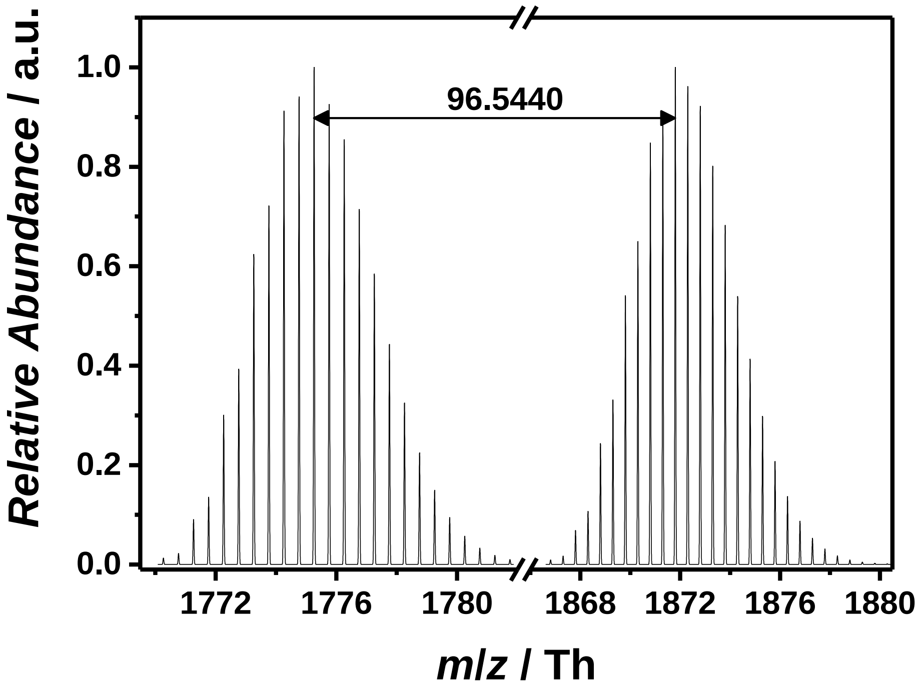


**Figure S57** Illustration of two simulated isotopic pattern representing the species at 2256 Th and 2631 Th. The difference between each highest peak corresponds to the theoretical value of the repeating unit.

**Tandem MS experiment of p([ATMEA]Cl) (10)**

A tandem MS experiment of p([ATMEA]Cl) (**10**) was not possible due to the low ion density. The collision-induced dissociation (CID) of 25 eV was sufficient to give both a spectrum of p([ATMEA]Cl) (**10**) and a cleavage of the vulnerable acrylate ester bond. The cleavage of 2-hydroxy-*N*,*N*,*N*-trimethylethan-1-aminium chloride can be considered as an indirect structural proof.

**Mass spectra of p([BVBIM]Cl) (11)**

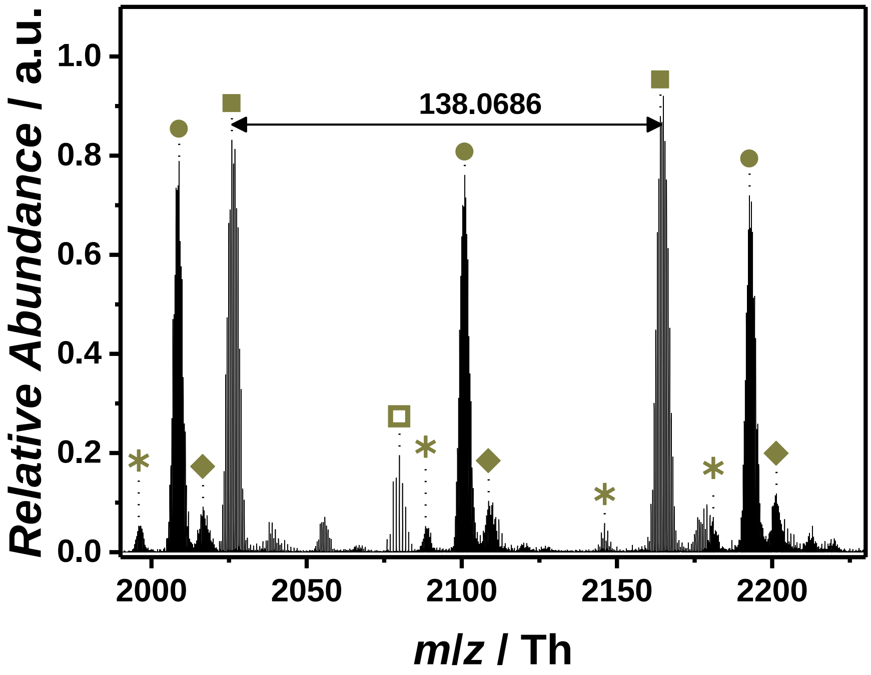


**Figure S58** Zoomed spectrum (negative mode) of p([BVBIM]Cl) (**11**) obtained *via* ESI-CID-Orbitrap MS depicting the repeating unit of $m/z$ = 138.0686 Th ($m/z$(theo) = 138.0702 Th) of the most abundant species (labelled with ■). Species labelled with ✳ derive from (multiple) loss(es) of gaseous HCl.

**Table S8** Peak assignment of the ESI-CID-Orbitrap spectrum of p([BVBIM]Cl) (**11**) from *m/z* = 2079 Th to *m/z* = 2165 Th showing the label (in correspondence to the species in **Figure S58**), the experimental *m/z* and theoretical *m/z* values (determined by the most abundant isotope of the isotopic pattern), Δ*m/z*, the resolution (obtained by the Xcalibur software), the number of repeating units *n*, and the structure determination. Due to the deprotonation process, no structure was determined for species labelled with ∗.

| Label | *m/z* (exp) [Th] | *m/z* (theo) [Th] | Δ*m/z* [Th] | Resolution | *n* | Structure |
|---|---|---|---|---|---|---|
| □ | 2079.9240 | 2079.9264 | 0.0024 | 46400 | 6 | 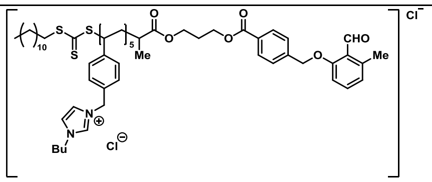 |
| ● | 2100.9830 | 2100.9840 | 0.0010 | 45400 | 20 | 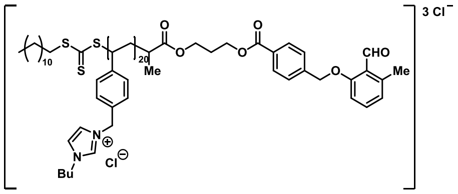 |
| ◆ | 2109.6604 | 2109.6703 | 0.0099 | 44700 | 21 | 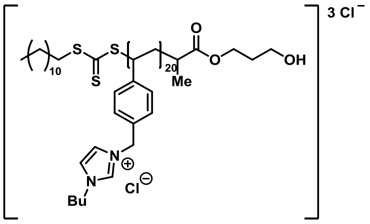 |
| ■ | 2165.0008 | 2165.0045 | 0.0037 | 43500 | 13 | 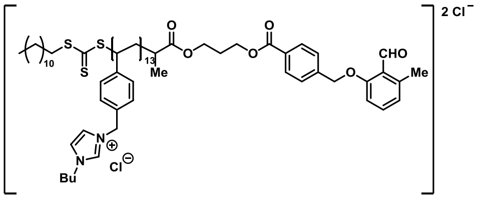 |

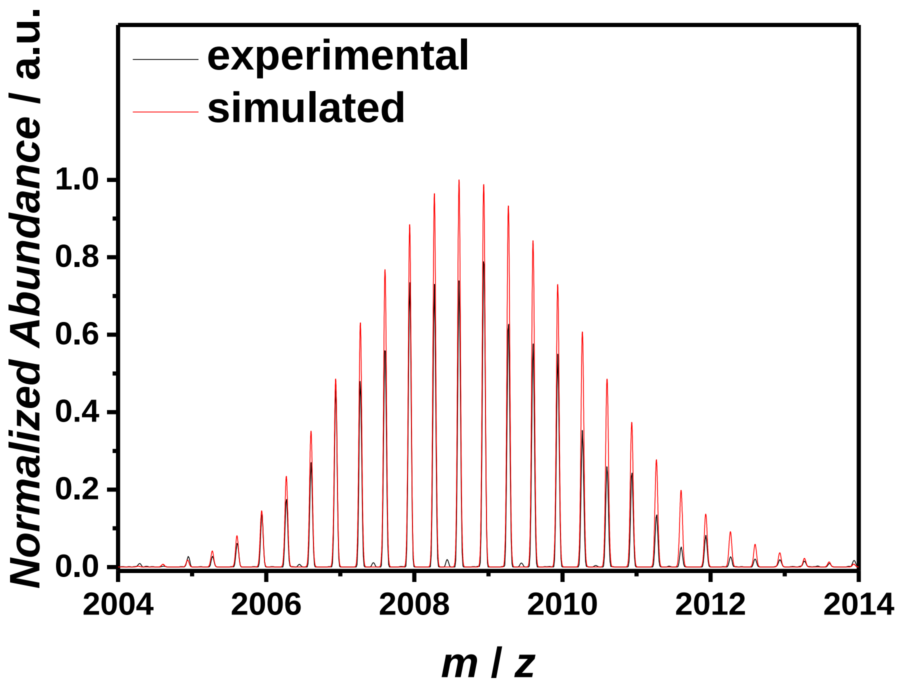


**Figure S59** Isotopic pattern of one selected peak at 2009 Th comparing the experiment (black line) and the simulation (red line) with a resolution of 48000.

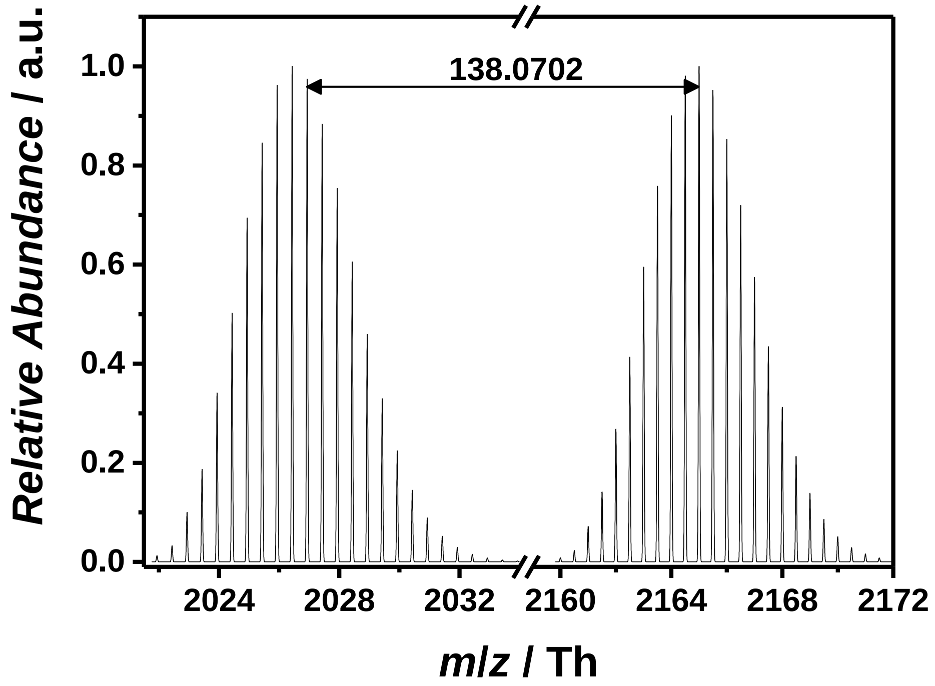


**Figure S60** Illustration of two simulated isotopic pattern representing the species deriving from disproportionation at 2026 Th and 2165 Th. The difference of the 98% intensity peak and the 100% intensity peak corresponds to the theoretical value of the repeating unit.

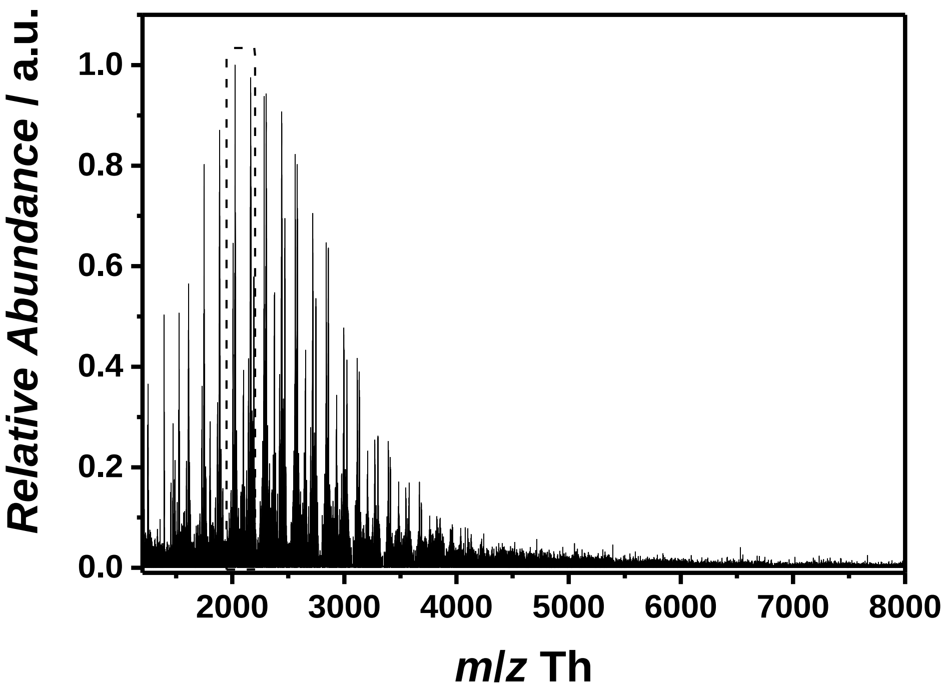


**Figure S61** Overview spectrum (negative mode) of p([BVBIM]Cl) (**11**) obtained *via* ESI-QToF MS utilizing $H_2O$/acetonitrile (1:1, *v*/*v*) as solvent.

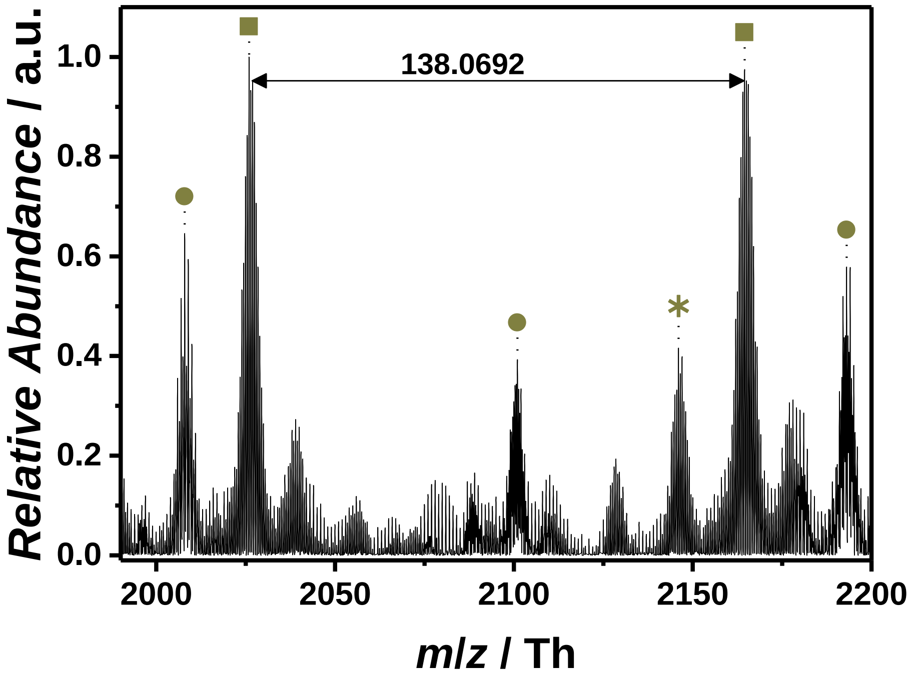


**Figure S62** Zoomed spectrum (negative mode) of p([BVBIM]Cl) (**11**) obtained *via* ESI-QToF MS depicting the repeating unit of *m*/*z* = 138.0692 Th (*m*/*z*(theo) = 138.0702 Th) of the most abundant species (labelled with ■). Species labelled with * derive from (multiple) loss(es) of gaseous HCl.

**Mass spectra of p([MVTr]I) (13)**

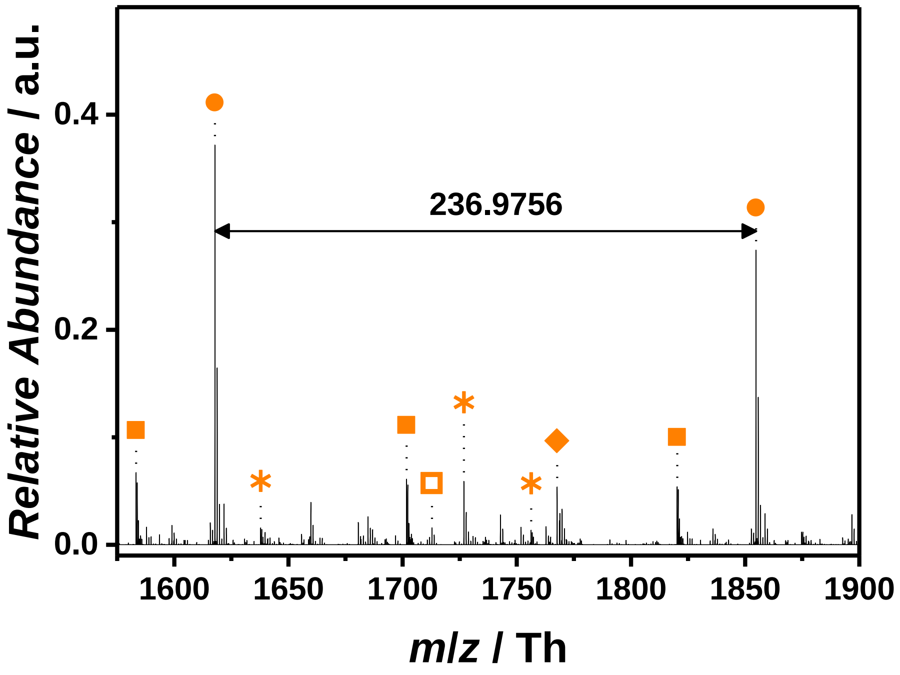


**Figure S63** Zoomed spectrum (negative mode) of p([MVTr]I) (**13**) obtained *via* ESI-CID-Orbitrap MS depicting the repeating unit of *m/z* = 236.9756 Th (*m/z*(theo) = 236.9763 Th) of the most abundant species (labelled with ●). Species labelled with ✳ derive from (multiple) loss(es) of gaseous HI.

**Table S9** Peak assignment of the ESI-CID-Orbitrap spectrum of p([MVTr]I) (**13**) from *m/z* = 1583 Th to *m/z* = 1767 Th showing the label (in correspondence to the species in **Figure S63**), the experimental *m/z* and theoretical *m/z* values (determined by the most abundant isotope of the isotopic pattern), Δ*m/z*, the resolution (obtained by the Xcalibur software), the number of repeating units *n*, and the structure determination (as representative candidate the saturated polymer chain was chosen). Due to the deprotonation process, no structure was determined for species labelled with ✳.

| Label | *m/z* (exp) [Th] | *m/z* (theo) [Th] | Δ*m/z* [Th] | Resolution | *n* | Structure |
|---|---|---|---|---|---|---|
| ■ | 1583.2922 | 1583.2917 | 0.0006 | 27700 | 12 | 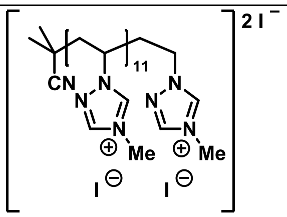 |
| ● | 1617.8189 | 1617.8206 | 0.0008 | 28200 | 6 | 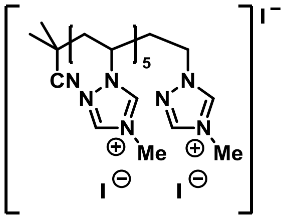 |

| | | | | | | |
|---|---|---|---|---|---|---|
| □ | 1712.8668 | 1712.8690 | 0.0021 | 24000 | 7 | 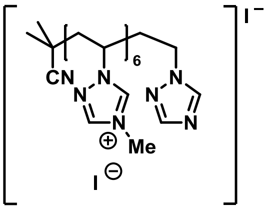 |
| ◆ | 1767.7148 | 1767.7148 | 0.0000 | 26300 | 6 | 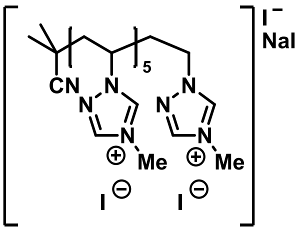 |

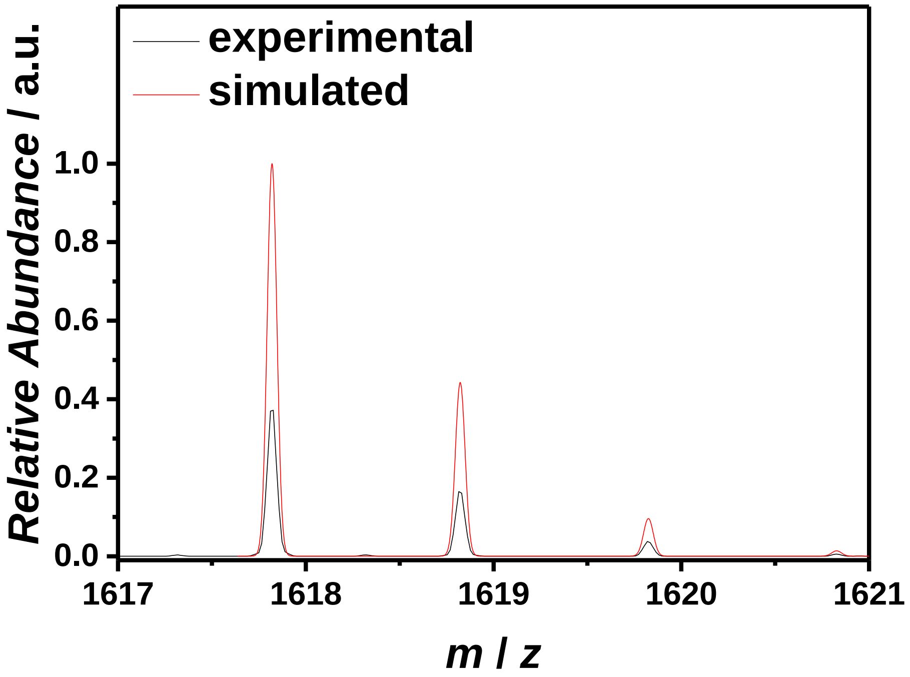


**Figure S64** Isotopic pattern of one selected peak at 1617 Th comparing the experiment (black line) and the simulation (red line) with a resolution of 28200.

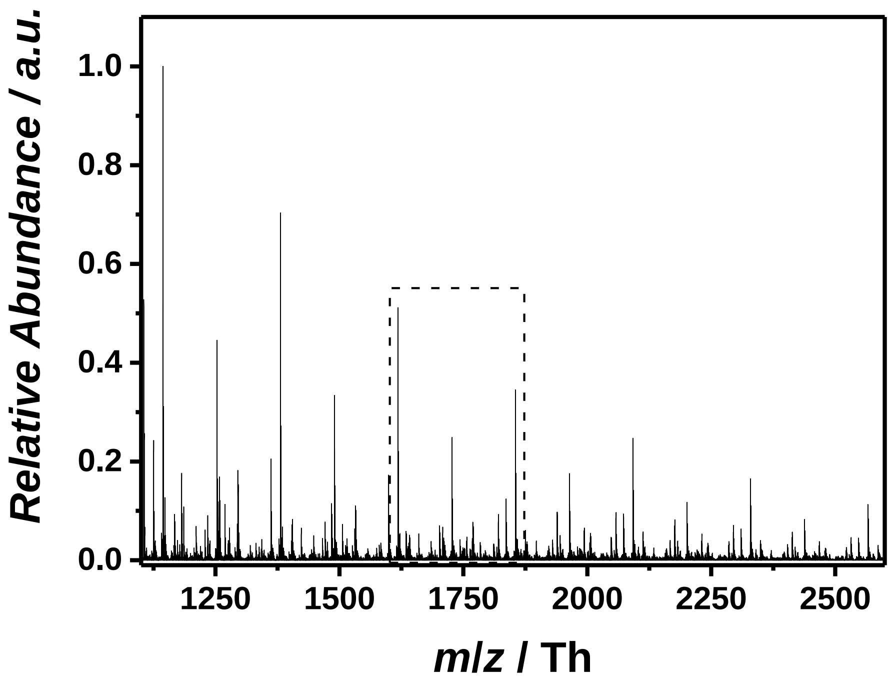


**Figure S65** Overview spectrum (negative mode) of p([MVTr]I) (**13**) obtained *via* ESI-QToF MS utilizing $H_2O$/acetonitrile (1:1, *v*/*v*) as solvent.

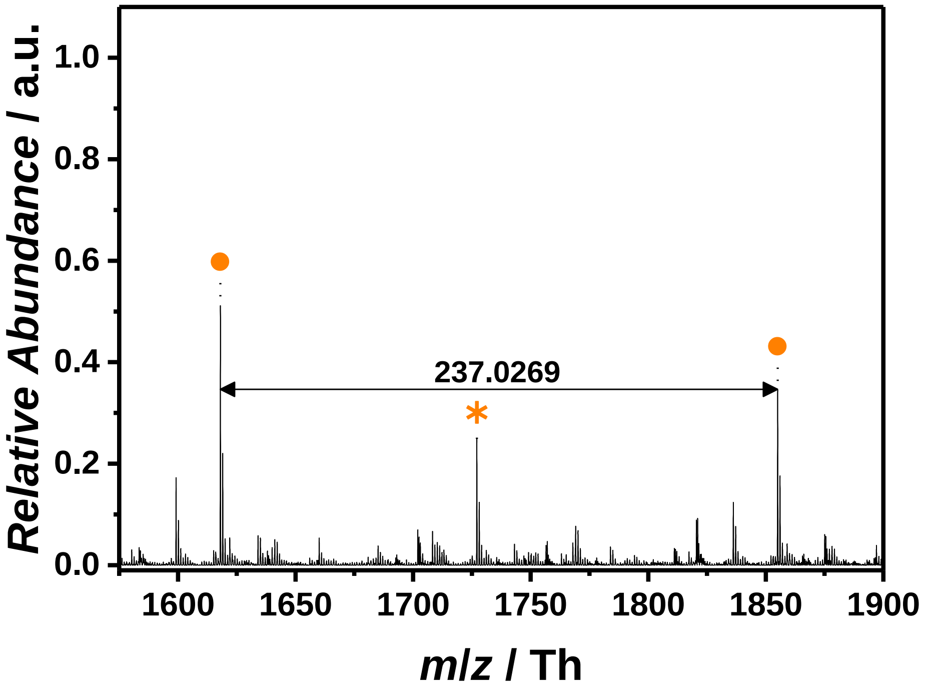


**Figure S66** Zoomed spectrum (negative mode) of p([MVTr]I) (**13**) obtained *via* ESI-QToF MS depicting the repeating unit of *m/z* = 237.0269 Th (*m/z*(theo) = 236.9763 Th) of the most abundant species (labelled with ●). Species labelled with * derive from (multiple) loss(es) of gaseous HI.

**Tandem MS experiment of p([MVTr]I) (13)**

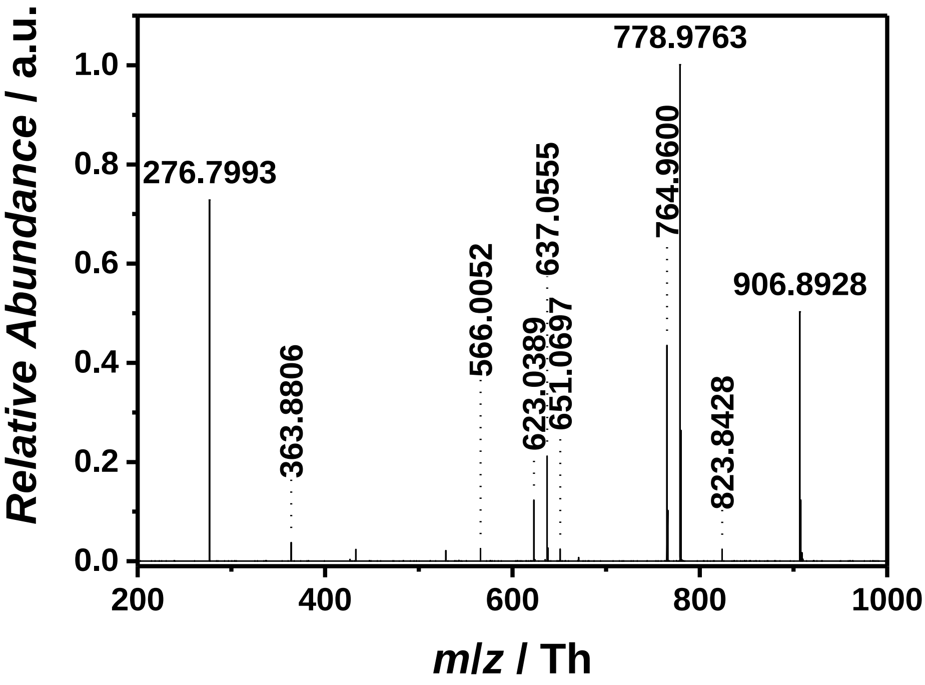


**Figure S67** Tandem MS experiment (negative mode) of a single charged species at 906 Th with a higher-energy collision dissociation (HCD) of 13 eV. For the complete fragmentation mechanism please refer to **Scheme S3**.

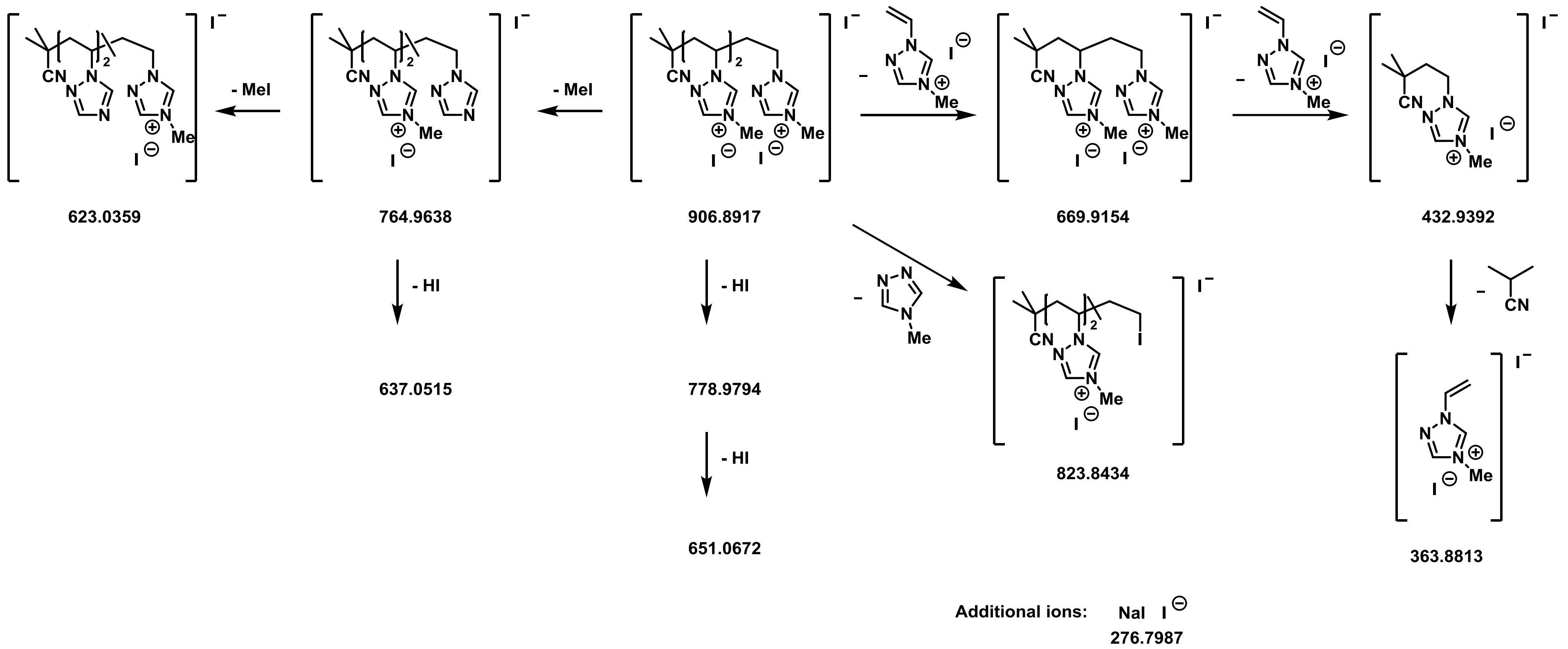


**Scheme S3** Proposed fragmentation of p([MVTr]I) (**13**) *via* three reaction pathways: a reverse Menshutkin mechanism including the stepwise nucleophilic attack of the iodide anion at the methyl moiety (loss of MeI); stepwise main chain depolymerization leading to the short-chained analogues of **13**; stepwise deprotonation *via* the release of gaseous HI.

**Mass spectra of p([BnVIM]Cl) (15)**

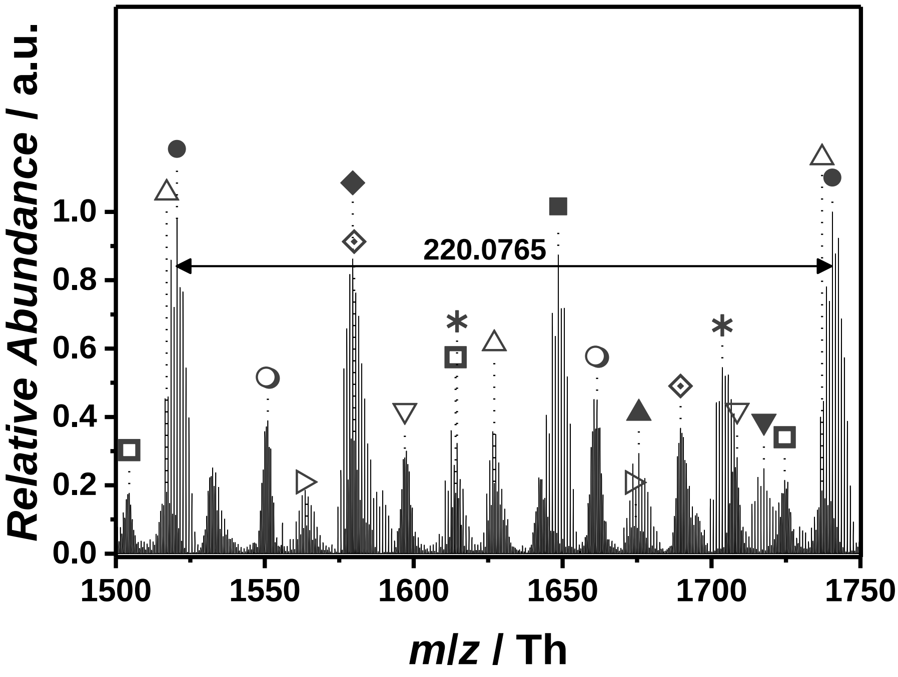


**Figure S68** Zoomed spectrum (negative mode) of p([BnVIM]Cl) (**15**) obtained *via* ESI-CID-Orbitrap MS depicting the repeating unit of *m/z* = 220.0765 Th (*m/z*(theo) = 220.0767 Th) of the most abundant species (labelled with ●). Species labelled with ✳ derive from (multiple) loss(es) of gaseous HCl.

**Table S10** Peak assignment of the ESI-CID-Orbitrap spectrum of p([BnVIM]Cl) (**15**) from *m/z* = 1504 Th to *m/z* = 1717 Th showing the label (in correspondence to the species in **Figure S68**), the experimental *m/z* and theoretical *m/z* values (determined by the most abundant isotope of the isotopic pattern), Δ*m/z*, the resolution (obtained by the Xcalibur software), the number of repeating units *n*, and the structure determination (as representative candidate the saturated polymer chain was chosen). Due to the deprotonation process, no structure was determined for species labelled with ✳.

| Label | *m/z* (exp) [Th] | *m/z* (theo) [Th] | Δ*m/z* [Th] | Resolution | *n* | Structure |
|---|---|---|---|---|---|---|
| □ | 1504.5024 | 1504.4947 | 0.0078 | 27800 | 13 | 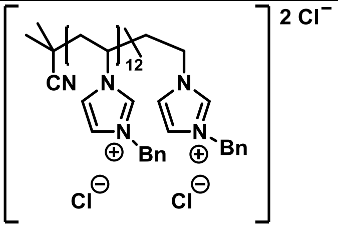 |
| ● | 1522.5273 | 1522.5378 | 0.0105 | 26100 | 7 | 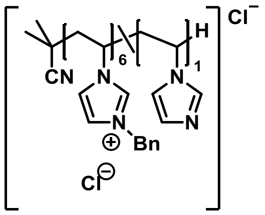 |

| | | | | | | |
|---|---|---|---|---|---|---|
| ◈ | 1579.0432 | 1579.0051 | 0.0382 | 28500 | 14 | 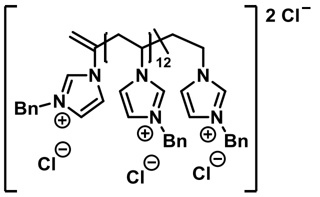 |
| ◆ | 1579.5655 | 1579.5034 | 0.0621 | 25300 | 7 | 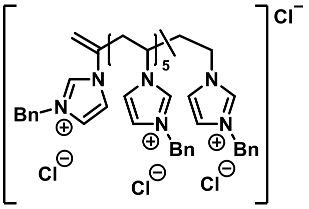 |
| ▷ | 1564.0478 | 1564.0189 | 0.0288 | 25800 | 15 | 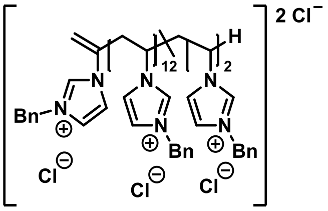 |
| △ | 1517.0268 | 1516.9923 | 0.0345 | 28500 | 14 | 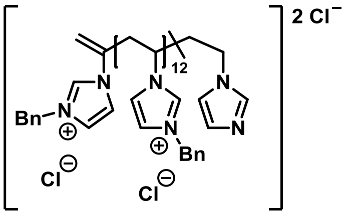 |
| ■ | 1648.5595 | 1648.5614 | 0.0019 | 25300 | 7 | 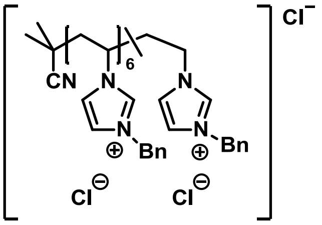 |
| ❍ | 1551.0228 | 1551.0215 | 0.0013 | 26900 | 14 | 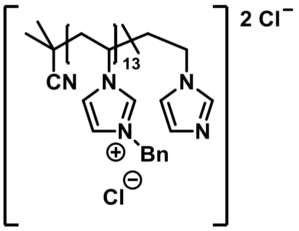 |
| ▽ | 1597.5482 | 1597.5488 | 0.0006 | 28800 | 15 | 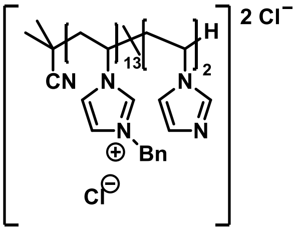 |
| ▼ | 1673.6112 | 1673.5567 | 0.0545 | 26300 | 7 | 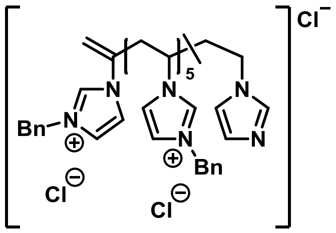 |

| | | | | | | |
|---|---|---|---|---|---|---|
| ▼ | 1717.6002 | 1717.6024 | 0.0022 | 27400 | 7 | 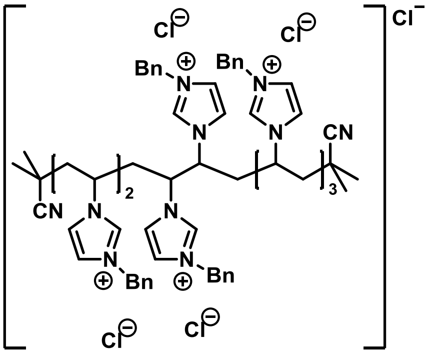 |

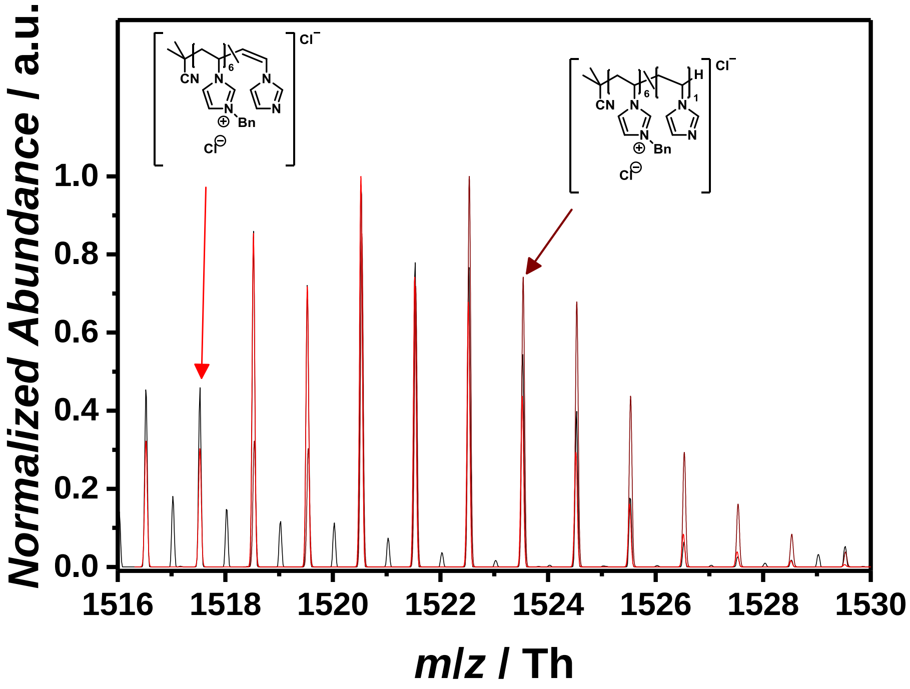


**Figure S69** Isotopic pattern of one selected peak at 1522 Th comparing the experiment (black line) and the simulation (red line) with a resolution of 26100. The isotopic pattern consists of both expected disproportion products.

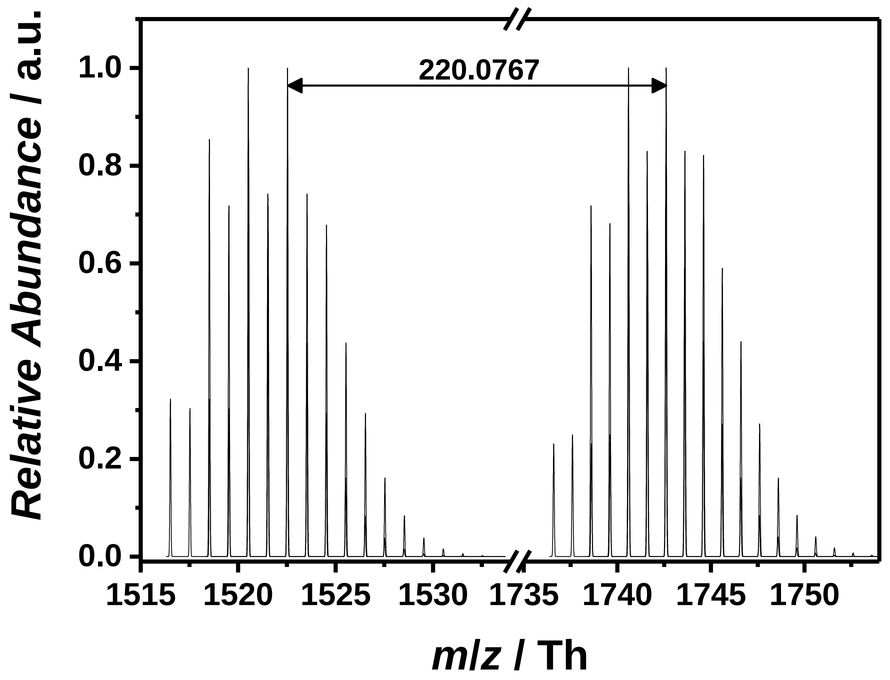


**Figure S70** Illustration of two simulated isotopic pattern representing the species deriving from disproportionation at 1520 Th and 1740 Th. The difference of each highest peaks corresponds to the theoretical value of the repeating unit.

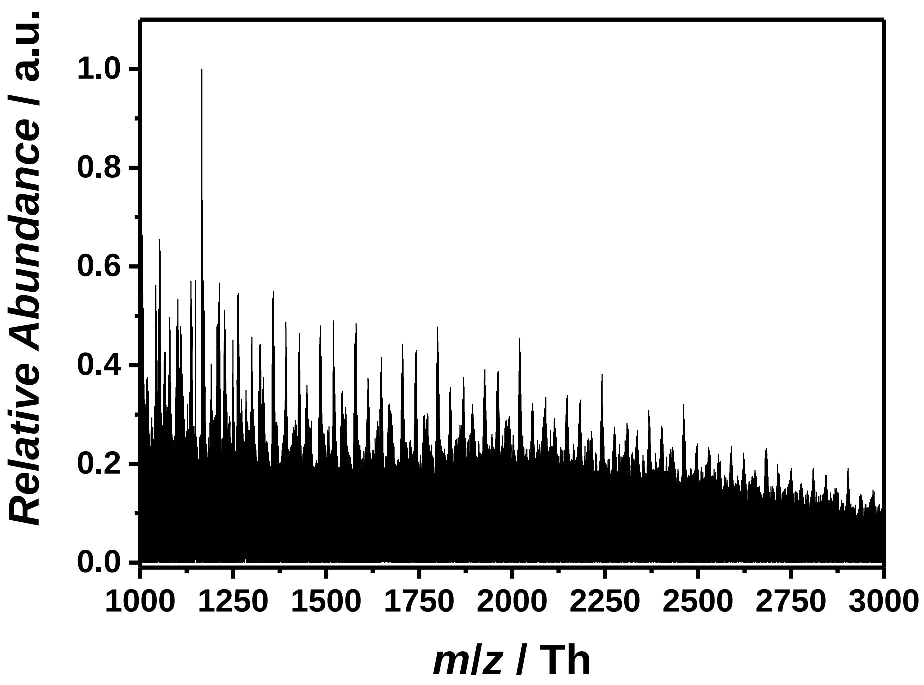


**Figure S71** Overview spectrum (negative mode) of p([BnVIM]Cl) (**15**) obtained *via* ESI-QToF MS utilizing $H_2O$/acetonitrile (1:1, *v*/*v*) as solvent. The low resolution in combination with various multiple charged species is due to the lower resolution of ESI-QToF.

## References

(1) Steinkoenig, J.; Bloesser, F. R.; Huber, B.; Welle, A.; Trouillet, V.; Weidner, S.; Barner, L.; Roesky, P.; Yuan, J.; Goldmann, A. S.; Barner-Kowollik, C. *Polym. Chem.* **2015**, 451–461.

(2) Kaupp, M.; Tischer, T.; Hirschbiel, A. F.; Vogt, A. P.; Geckle, U.; Trouillet, V.; Hofe, T.; Stenzel, M. H.; Barner-Kowollik, C. *Macromolecules* **2013**, *46*, 6858–6872.
